# Identification of a new regulation pathway of EGFR and E-cadherin dynamics

**DOI:** 10.1038/s41598-021-02042-3

**Published:** 2021-11-22

**Authors:** Veronique Proux-Gillardeaux, Tamara Advedissian, Charlotte Perin, Jean-Christophe Gelly, Mireille Viguier, Frederique Deshayes

**Affiliations:** 1 CNRS, Institut Jacques Monod, Université de Paris, F-75013 Paris, France; 2 Membrane Traffic and Cell Division Laboratory, Institut Pasteur, UMR3691, CNRS, 75015 Paris, France; 3 Université de Paris, UMR_S1134, BIGR, Inserm, 75006 Paris, France; 4 Institut National de Transfusion Sanguine, 75015 Paris, France

**Keywords:** Biochemistry, Cell biology

## Abstract

E-cadherin and EGFR are known to be closely associated hence regulating differentiation and proliferation notably in epithelia. We have previously shown that galectin-7 binds to E-cadherin and favors its retention at the plasma membrane. In this study, we shed in light that galectin-7 establishes a physical link between E-cadherin and EGFR. Indeed, our results demonstrate that galectin-7 also binds to EGFR, but unlike the binding to E-cadherin this binding is sugar dependent. The establishment of E-cadherin/EGFR complex and the binding of galectin-7 to EGFR thus lead to a regulation of its signaling and intracellular trafficking allowing cell proliferation and migration control. In vivo observations further support these results since an epidermal thickening is observed in galectin-7 deficient mice. This study therefore reveals that galectin-7 controls epidermal homeostasis through the regulation of E-cadherin/EGFR balance.

## Introduction

Galectin-7 is a member of the galectin family encompassing soluble lectins with a large variety of ligands and functions. In contrast to certain galectins which are widely expressed in various tissues galectin-7 expression is restricted to pluristratified epithelia such as the epidermis^1,2^. Furthermore this lectin is observed both in the extracellular compartment and in the cytosol, in mitochondria and even in the nucleus^3^. Galectin-7 is involved in multiple functions^3^ such as keratinocyte proliferation and differentiation^4^, wound healing^5,6^, cell migration and control of cell adhesion through direct interaction with E-cadherin^7–9^. Galectin-7 has been reported in several cancers as a marker but also as an actor in tumor progression^10,11^.

E-cadherin, a 120 kDa molecule, is one of the major constituents of adherent junctions. At the epithelial cell surface, trans-membrane E-cadherin associates with E-cadherin molecules from adjacent cell ensuring intercellular cohesion and communication. Different proteins have been found to regulate E-cadherin stability at the plasma membrane including β-catenin, p120-catenin, galectin-7 or tyrosine kinase receptors. Indeed, many studies suggest that Epidermal Growth Factor Receptor (EGFR) is involved in molecular complexes with E-cadherin and catenins^12,13^. These studies demonstrate that while EGFR activation can disrupt E-cadherin function, E-cadherin can reversely antagonize EGFR activity^14,15^. Moreover, these results indicate a dynamic relationship between EGFR and E-cadherin that regulates the function of both molecules.

EGFR is a cell-surface tyrosine kinase receptor that plays a fundamental role regulating cellular metabolism, growth and differentiation by initiating a complex signal transduction cascade^16^. After EGF binding EGFR is phosphorylated, ubiquitinated and internalized. The major EGFR downstream signaling pathways include the mitogen-activated protein kinase (MAPK), the phosphoinositide 3-kinase (PI3K)/Akt, the phospholipase C (PLC), the Janus kinase (JAK), and the signal transducer and activator of transcription (STAT) proteins^17^. Deregulated EGFR signaling and trafficking have been associated with numerous types of cancer^18^. EGFR extracellular domain consists of four subdomains following the signal peptide. These domains are heavily decorated with N-glycans and are involved in cancers notably as a result of aberrant or excessive glycosylation^19,20^, inappropriate *N*-glycosylation often resulting in EGFR dysfunction^21,22^. These glycosylations are major regulators of growth factor binding to EGFR^23^ and EGFR is a target of therapy including its inhibition by small molecules interfering with its intracellular kinase domain or by antibodies directed against its extracellular region^24^. Interestingly, regarding carbohydrates, galectin-7 displays preferential binding to internal or terminal LacNAc repeats carried by N-glycan^25,26^, highly represented in EGFR extracellular domain.

In this study, we discover that EGFR, galectin-7 and E-cadherin form a complex in vitro and propose an original in silico structural modeling for these interactions. Interestingly, we also unveil that galectin-7 is a direct partner of EGFR via interactions through carbohydrate domains and decipher the relationship between EGFR and galectin-7 and the physiological consequences for homeostasis. By focusing on downstream signaling and subsequent EGFR endocytosis we demonstrate that galectin-7 is involved in the regulation of EGFR phosphorylation and trafficking.

## Results

### Galectin-7 directly links E-cadherin to EGFR

**Figure 1:**
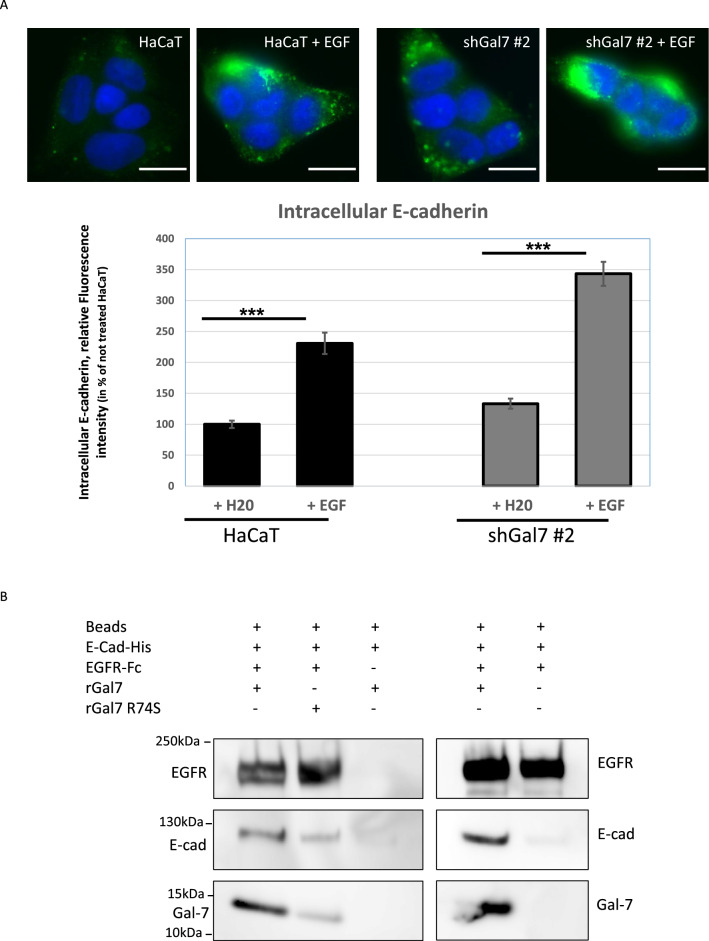
Galectin-7 interacts with both E-cadherin and EGFR and modulates E-cadherin endocytosis. (**A**) Modulation of E-cadherin internalization by galectin-7 and EGF. Representative images of HaCaT and ShGal7 #2 after E-cadherin antibody uptake for 30 min. Cells were previously treated or not with 100 ng/mL of EGF. Histograms represent corresponding quantifications in percentage reported to HaCaT not treated cells. Scale bars stand for standard deviation. (**B**) In vitro binding assays were performed using recombinant wild-type human galectin-7 (rGal7); CRD mutated human galectin-7 (R74S), Extracellular domain of human E-cadherin fused to a His tag (E-cad-his) and extracellular domain of human EGFR fused to human IgG1 Fc fragment (EGFR-Fc). In each conditions, EGFR-Fc was pulled down using protein G sepharose coated beads. E-cadherin precipitated with EGFR-Fc only in presence of galectin-7 (n = 3). Cropped images are from samples run on the same gels. Full-length blots are displayed in supplementary Fig. S1B.

We previously documented that galectin-7 binds to E-cadherin and regulates its dynamics at the plasma membrane^7^. Moreover overexpression of EGFR correlates with perturbation of the E-cadherin/catenin complex suggesting an underlying functional interaction between growth-regulatory factors and E-cadherin with consequences on the balance between proliferation and differentiation as illustrated by the results of Wilding et al.^27^. Hence, we decided to study a potential interaction between galectin-7, EGFR and E-cadherin using HaCaT cell line, an immortalized keratinocyte human cell line in which we have ensured that E-cadherin and EGFR co-precipitate (Fig. S1A). We generated HaCaT stable clones with a highly reduced galectin-7 expression by shRNAs gene silencing as previously described (ShGal7)^7^. We performed an E-cadherin internalization assay in presence or absence of EGF and measured the internal intensity of fluorescence corresponding to E-cadherin. As previously described^7^, at basal level E-cadherin is internalized with better efficiency in absence of galectin-7. In presence of 100 ng/ml EGF we observed that E-cadherin is 2.3 times more efficiently internalized in HaCaT cells when compared to cells without EGF (Fig. 1A). E-cadherin endocytosis is more intense in cells depleted of galectin-7. Thus galectin-7 has effects similar to those of an EGF antagonist on E-cadherin endocytosis and does negatively regulate the E cadherin endocytosis in absence as in presence of EGF. These results prompted us to consider that galectin-7 could form a tripartite complex between E-cadherin and EGFR as EGFR was pinpointed as a potential partner of galectin-7 in our preliminary proteomic study.

We thus realized in vitro binding assays with purified proteins. Recombinant human galectin-7 (rGal7) was incubated with a recombinant chimeric E-cadherin containing the E-cadherin ectodomain (Asp155-Ile707) fused to a C-terminal 6-Histidine tag (E-cad-His) and with the EGFR ectodomain (Met1-Ser645) fused to Fc fragment (Human IgG1-Fc (Pro100-Lys330) (EGFR-Fc). Experiments were conducted with protein G sepharose in such a way that only EGFR-Fc can bind to the beads. Strikingly, galectin-7 and E-cadherin were precipitated by EGFR extracellular domain (Figs. 1B, S1B). However, although the binding between E-cadherin and galectin-7 is sugar independent^7^, mutated form of galectin-7 with a R74S substitution in the carbohydrate recognition domain (CRD) faintly precipitated in vitro with recombinant EGFR-Fc and E-cadherin. These results strongly support a direct interaction between these three proteins reinforcing the concept of a tripartite complex. We thus checked the direct inding of galectin-7 and EGFR in vivo.

### Galectin-7 directly interacts with EGFR in vivo and in vitro through glycosylation of its extracellular domain

**Figure 2:**
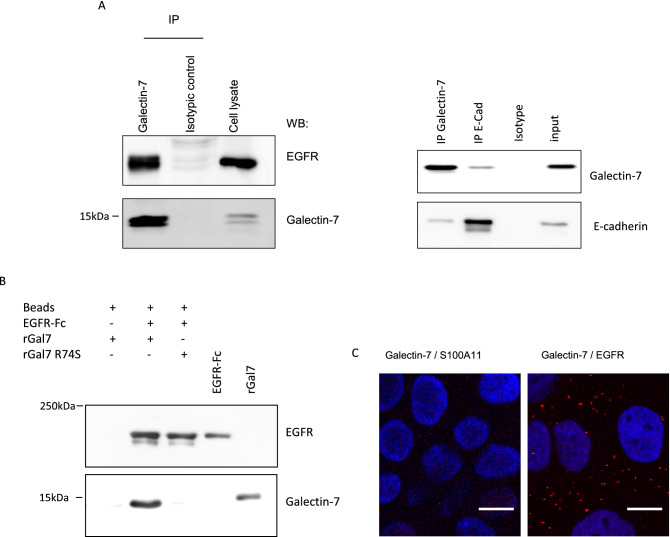
Galectin-7 interacts and colocalizes with EGFR. (**A**) Co-immunoprecipitation experiments indicate that galectin-7 is a partner of EGFR and E-cadherin. Images shown are representative of images taken from distinct western blots. Full-length blots are displayed in supplementary Fig. S2A.Galectin-7 directly interacts with extracellular domain of E-cadherin independently of glycosylation motifs. (**B**) In *vitro* binding assays were performed using recombinant wild type human galectin-7 (rGal7); CRD mutated human galectin-7 (R74S) and extracellular domain of human EGFR fused to human IgG1 Fc fragment (EGFR-Fc). WT galectin-7 (rGal7) precipitated with EGFR-Fc unlike mutated galectin-7 (R74S) Full-length blots are displayed in supplementary Fig. S2B. (**C**) Confocal images of Proximity Ligation Assays confirming that galectin-7 is in close proximity with EGFR in cellular context. Galectin-7—S100A11 pairs were used as negative controls. At least 3 independent experiments were conducted.

We therefore performed immunoprecipitation of galectin-7 followed by western blots revealing that galectin-7 co-precipitates EGFR in addition to E-cadherin in HaCaT cells (Figs. 2A, S2A). To better define the relationship between EGFR and galectin-7, we performed in vitro pull-down experiments with purified proteins. As it can be observed on Figs. 2B and S2B, galectin-7 has been retained by EGFR-FC, demonstrating a direct interaction between these two proteins. As EGFR is known to be heavily glycosylated and because of the above results, we thus explored the importance of these glycosylation motifs for galectin-7 interaction by using galectin-7 R74S mutant. As shown on Figs. 2B and S2B, this mutated lectin-deficient form of galectin-7 is not co-precipitated with EGFR-FC in vitro. Hence this defective binding with the CRD-mutant ascertains a glycosylation-dependent interaction between galectin-7 and the EGFR extracellular domain.

In order to further characterize this interaction in living cells, we performed a proximity ligation assay (PLA) allowing the visualization of interactions between two proteins in close proximity (less than 40 nm) with the appearance of red fluorescent dots at the location of their interaction. As observed in Fig. 2C, galectin-7 and EGFR do interact under these conditions. In order to better characterize the interaction inside this tripartite complex, we decided to study this interaction in silico.

### In silico assessment and modeling of Galectin-7, E-cadherin complex and insights for EGFR interaction

To further decipher and assess this interaction, we have conducted in silico modeling between E-cadherin, EGFR and galectin-7 based on previous experiments^7^.

**Figure 3:**
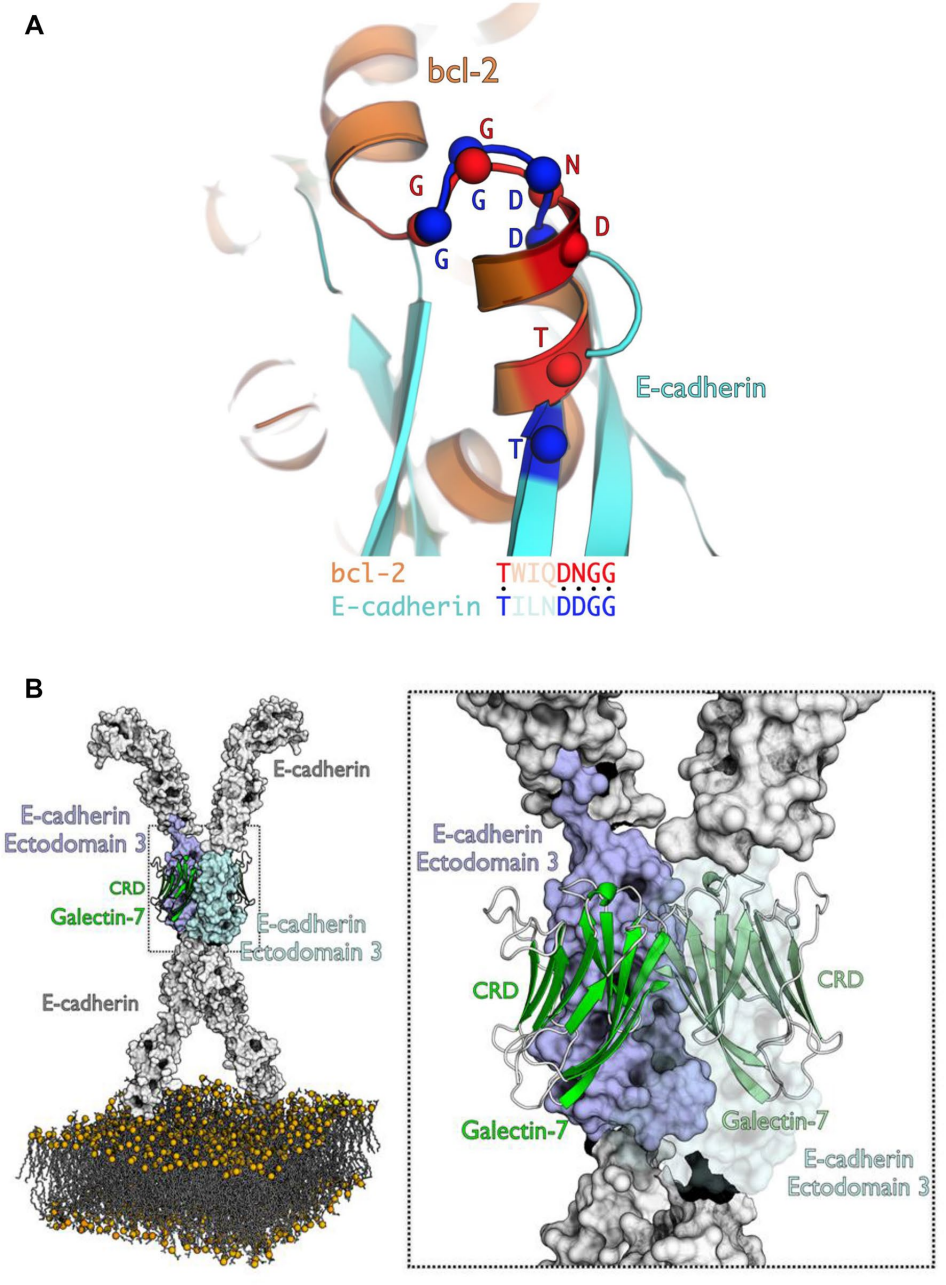

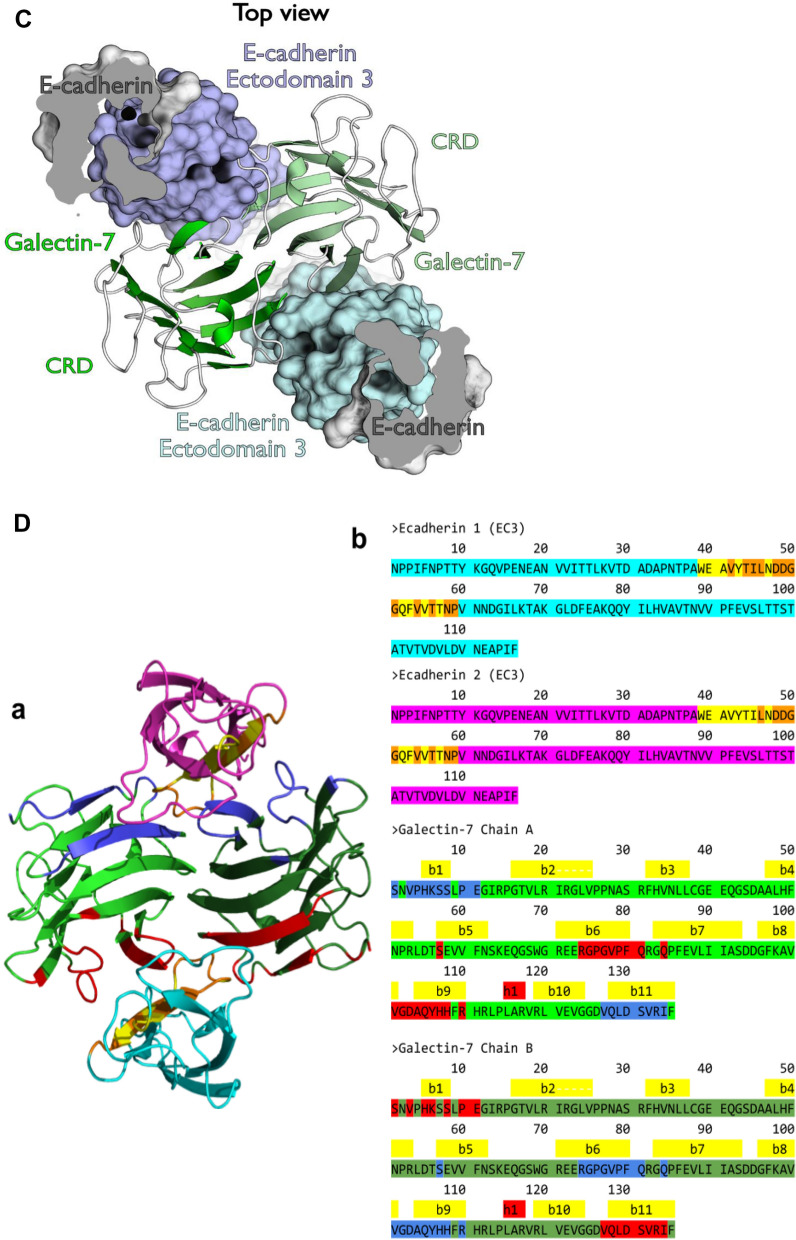

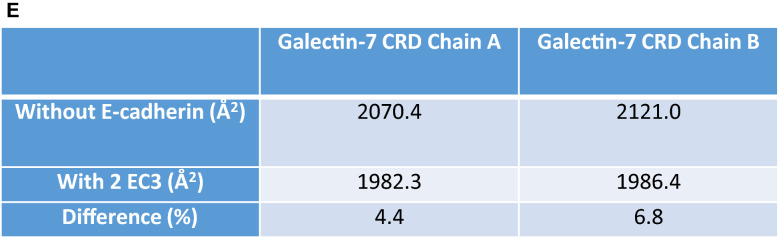
Galectin-7 interacts with both EGFR and E-cadherin. (**A**) Structural alignment of sequence motifs found in E-cadherin ectodomain 3 and Bcl-2. The threonine residues, as well as the DXGG motif can be structurally aligned with high precision. Cα of aligned residues are represented as spheres. (**B**) In silico model of interaction of two E-cadherin molecules on both sides of galectin-7 homodimer placed on the plasma membrane. Galectin-7 chains are presented in green shades, E-cadherin ectodomains in light grey. Ectodomains 3 are colored in pale violet and blue. A zoom on the binding region can be seen on the right. On the zoom, pale blue E-cadherin 3 ectodomain is displayed in transparency mode to better observe the interaction interface. (**C**) Top view of two E-cadherin molecules interacting with galectin-7. The same color scheme of Fig. 3 has been used. The two N-ter E-cadherin ectodomains are cut for clarity. (**D**) (**a**) First docking poses on the galectin-7 dimer for the first and second ectodomain 3 (EC3) of E-cadherin, in cyan and magenta, respectively. Chains of galectin-7 dimer are represented in green shades. Sequence motif identified in E-cadherin in colored in yellow. In E-cadherin, interface frequent residues are colored in orange. Interface frequent residues made by galectin-7 with the first E-cadherin molecule are in red; those with the second molecule are in blue. (**b**) Sequences of the two EC3 molecules and the galectin-7 dimer are displayed. Sequences and contacts are colored as described above. (**E**) Relative solvent accessibility in of CRD domains of galectin-7 (defined by residues S1-P10, S30-P76 and L120-V127) with and without two E-cadherin molecules according to Naccess.

Because galectin-7 interacts with EGFR through its CRD domain, interaction between galectin-7 and E-cadherin should take place at distance of the CRD domain. Bcl2 being the only already described protein interacting with galectin-7 independently of its CRD domain, we investigated if the glycosylation-independent interaction between E-cadherin and galectin-7 might echo the Bcl-2/galectin-7 interaction. Interestingly, even if Bcl-2 and E-cadherin do not share global similarity, they share a common motif of 47 residues with 26% identity at the level of E-cadherin extracellular domain 4 (EC4) which was identified by local alignment on E-cadherin extracellular sequence. Similarly, when aligning E-cadherin domain 3 (EC3) with Bcl-2 using the same protocol, another motif of 25 residues can be identified which shares 30% identity between each other (fig. S3A,B). Furthermore, a series of surface residues of E-cadherin, important for interaction between galectin-7 and E-cadherin, can be successfully structurally superposed on Bcl-2, confirming a similar interaction mode (Fig. 3A). These observations suggest that galectin-7 interaction with E-cadherin might be mediated by the latter domains 3 or 4. Docking simulations with either domain 3 or domain 4 allow to identify most probable interface residues. While recurring residues of domain 4 do not constitute a patch on the motif found by local sequence alignment, 50% of domain 3 motif residues are part of the interface in at least 1000 docking poses even though this domain is smaller than EC4. These residues, when mutated in silico, affect E-cadherin binding mode (data not shown), confirming their fundamental role in the interaction. Interestingly, the most impacting mutations are those affecting several residues at the same time, particularly those impacting the DDGG motif of Ecad, equivalent to the DNGG found in Bcl-2 (Fig. 3A). Moreover, the Bcl-2 motif identified in E-cadherin domain 4 is buried and thus not accessible to bulky residues of Bcl-2 structure (PDB ID: 2XA0). On the contrary, the motif found in domain 3 lies on the surface of the protein. Thus, while E-cadherin and Bcl-2 interaction with galectin-7 are similar these findings suggest that E-cadherin domain 3 is the most probable domain able to interact with galectin-7 which we confirmed by docking simulations. Furthermore, model of ectodomain 3 has good energy values all along the sequence and the motif of interest forms a β hairpin offering a great surface for an interaction.

Molecular docking experiments have led to an interesting pose that supports galectin-7/E-cadherin interaction. The best pose obtained is supported both in terms of energy (total docking score = 3812) and in terms of statistical distribution of score values for the best first thousand poses obtained (Z-score = 5.20). This interaction model also corresponds to a region where the density of interaction in terms of pose is the highest (Fig. S3B) suggesting that the physico-chemical characteristics of the regions involved in the interaction are favorable and converge towards the optimal pose obtained. Moreover, the most interacting residues of E-cadherin in the top 1000 docking poses include the ones composing the sequence motif identified previously. In addition, these residues were also at the interface in the best pose (Fig. S3C). All these results greatly support our molecular complex model.

We observed that E-cadherin extracellular domain 3 interacts with galectin-7 at the level of its dimerization interface (Fig. 3B–D). This region is found twice in galectin-7 and adopts a symmetrical arrangement which allows the binding of two E-cadherin molecules on both sides of galectin-7 (Fig. 3B–D). The binding of the two E-cadherin molecules is made in a symmetrical manner when docking first pose is considered. This pose is the most relevant, with a docking score highly superior to others (Fig. S3D). Thus both CRD domains of each galectin-7 are free to interact with sugars. The computation of solvent accessibility of galectin-7 CRD with and without the two E-cadherin molecules docked confirms that there are no significant differences (Fig. 3E). It confirms the glycosylation-independent interaction mode of galectin-7 to E-cadherin. Finally, it also supports the capacity of galectin-7 to bind E-cadherin independently of its CRD and EGFR through sugars at the same time.

EGFR is known to adopt particularly complex and flexible conformations, and can be observed as monomers and as dimers which have particular impacts for EGFR functions^28^. Interestingly, such interaction between E-cadherin and EGFR through galectin-7 would modify EGFR degree of freedom and therefore impact its dynamics and functions as it has been previously demonstrated in molecular dynamic simulation *inter alia*^28^*.* Also, even if it was not resolved in experimental structures, EGFR conformation is modulated by the binding of more than 10 long N-glycans. These types of sugars have very diverse lengths and molecular weights. Here, without experimental information about these sugars, a precise atomistic model cannot be proposed. However, galectin-7/E-cadherin interaction region is near 100 Å away from the membrane, a distance compatible with the apex part of EGFR. Considering that N-glycans glycosylating human EGFR can have very long chains, one can imagine a long-range interaction (< 40 nm as indicated by PLA) mediated by sugar chains.

### Galectin-7 depletion favors EGFR phosphorylation and impact its downstream signaling

**Figure 4:**
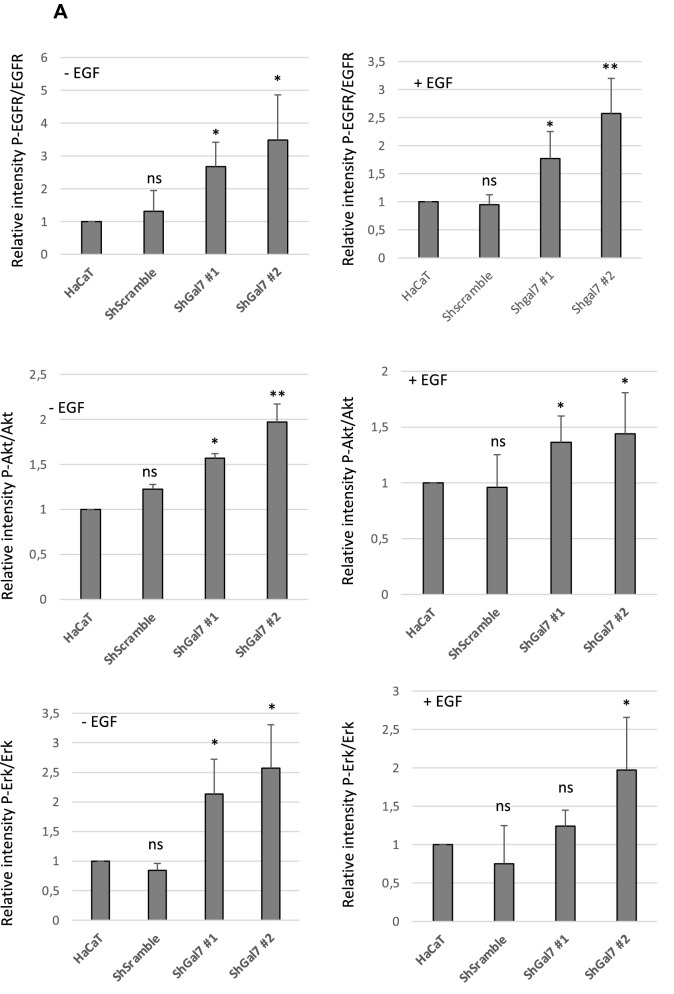

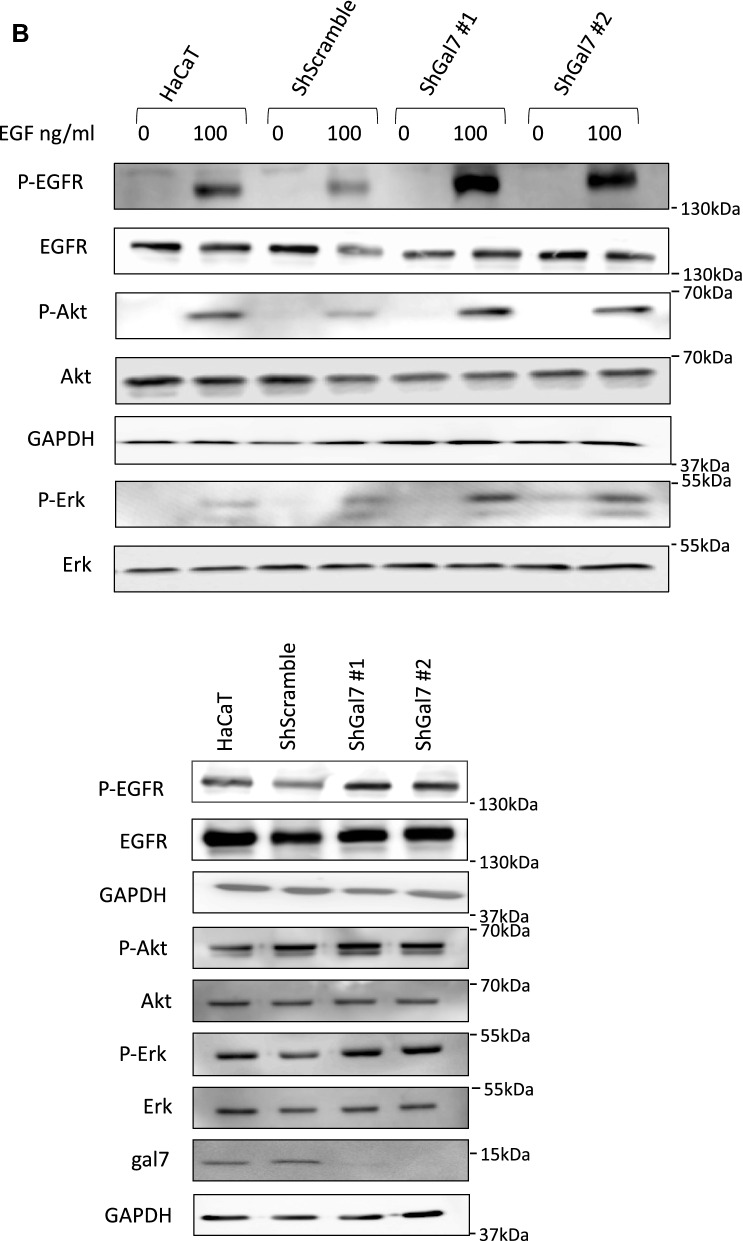

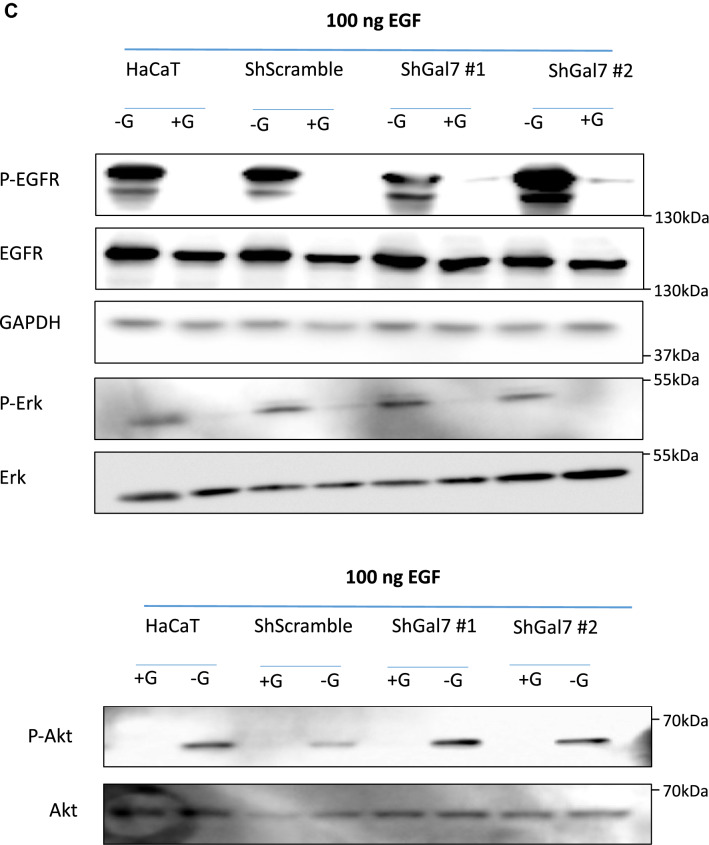
Aberrant EGFR phosphorylation by galectin-7 deficient cells is the cause for alteration of downstream pathways. (**A**) Quantification of the blots of Figure (**B**) are representative of at least 3 independant experiments. Mean ± SEM : * *p* < 0.1 **p < 005 ***p < 0001 (**B**) Cells were treated or not with EGF (100 ng/mL) for 15 mn before being lysed. Immunoblots were probed for phospho EGFR (Y1068), total EGFR, phospho-Erk (p-ERK) total Erk, phopho-Akt, total Akt and GAPDH. Untreated cells were also blotted separately (lower panel) to better quantify the signal. Cropped images are from samples run on the same gels. Full-length blots are displayed in supplementary Fig. S4C. (**C**) Cells were incubated with EGF and with 15 µM of gefitinib for 15 mn before being lysed. Immunoblots were probed for phosphor-EGFR (Y1068), total EGFR, phospho-Erk (p-Erk) total Erk, phopho-Akt, total Akt and GAPDH. Cropped images are from samples run on the same gels. Full-length blots are displayed in supplementary Fig. S4D. Scale bars stand for standard deviation.

As we previously described the functional effect of galectin-7 binding to E-cadherin^7^, we examined this effect on EGFR. We thus studied EGFR phosphorylation level in both control and ShGal7 clones. After an overnight starvation to free cells from growth factors, we treated cells with 100 ng/ml of EGF or diluent during 15 min and cell extracts were submitted to Western blot analysis. Remarkably, in absence of exogenously added EGF, EGFR displays a threefold basal activation levels in ShGal7 cell lines in comparison to HaCaT cells (Figs. 4A,B lower panel, S4D). This is not due to a higher amount of EGFR present at the plasma membrane as assessed by a cell surface biotinylation assay (data not shown). Thus galectin-7 plays a major role in the regulation of EGFR phosphorylation basal level. EGFR possesses multiple downstream targets such as Src, Akt or ERK. Hence, we compared the activation of these different pathways in presence or absence of galectin-7. We did not observe any difference on Src pathway (Fig. S4A) but we observed an increased phosphorylation on ERK and Akt in cells deficient for galectin-7 (figs. 4A,B, S4D). Thus galectin-7 restrains basal level of signaling pathways depending on EGFR phosphorylation.

When adding 100 ng of EGF for 15 min, a strong increase of EGFR phosphorylation was observed both in presence and in absence of galectin-7. For instance, activation of EGFR phosphorylation upon addition of EGF showed a 37 fold increase in HaCaT cells (Fig. S4B). In absence of galectin-7 EGFR phosphorylation as well as Akt and Erk pathways showed the same tendency in the different conditions as in absence of EGF reinforcing the previous data showing that galectin-7 impairs EGFR phosphorylation and its downstream pathways. However these increases seem to be limited in presence of EGF which could be due to an activation plateau. In these experimental conditions we also detected an increase of STAT3 pathway (Fig. S4C) but Src pathway was still not activated (Fig. S4A). In order to reinforce these observations we repeated these experiments in presence or in absence of gefitinib, a specific inhibitor of EGFR tyrosine kinase activity hence ensuring that indeed they do depend on EGFR activity. As shown in Figs. 4C and S4A, S4C, S4E all the described pathways except Src are activated in a stronger manner in the absence of galectin-7.

These results hence points out that the interaction between EGFR and galectin-7 restrains EGFR constitutive and ligand-inductible phosphorylation and thus its downstream pathways.

As phosphorylation level has multiple consequences on receptors, we examined these different parameters.

### Galectin-7 depletion favors EGFR ubiquitination but not degradation

**Figure 5:**
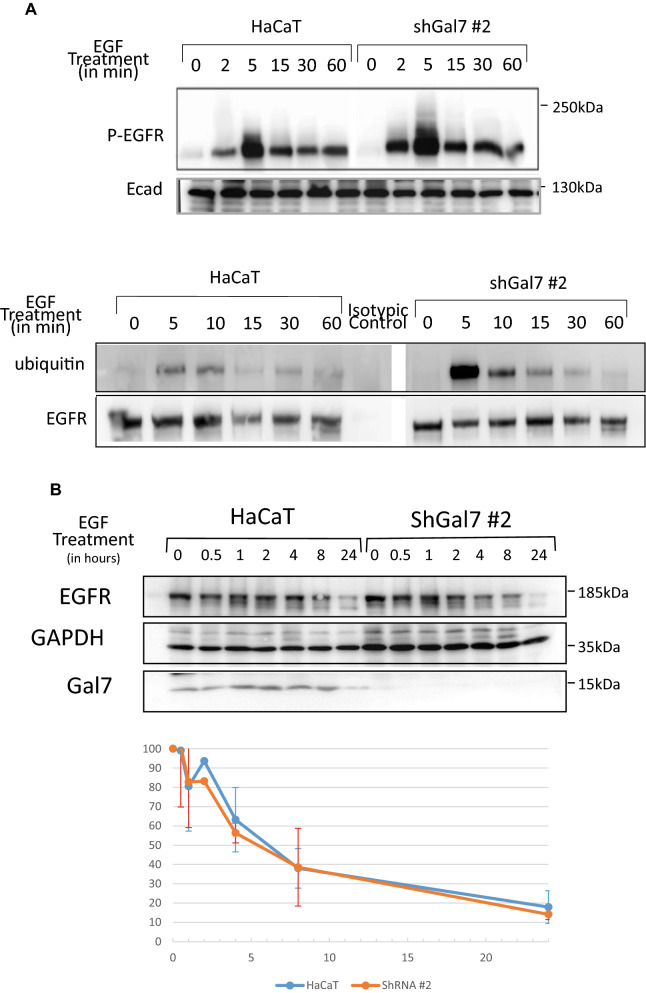

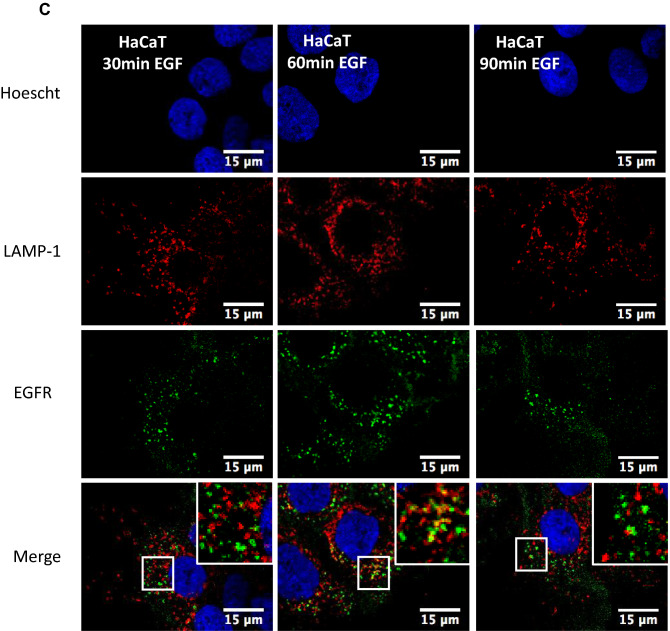

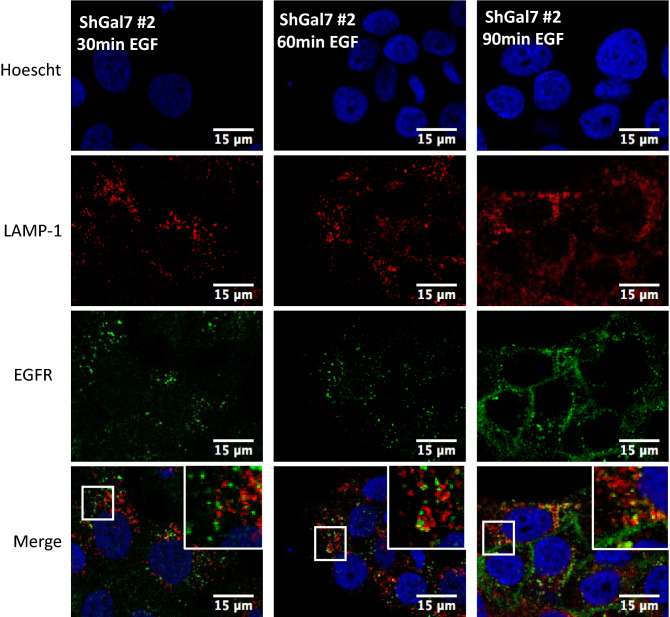
Upregulation of EGFR phosphorylation and ubiquitination in absence of galectin-7 after EGF treatment. (**A**) Time course of EGFR activation by EGF in HaCaT or shGal-7 cells treated with 100 ng/mL EGF. Whole cell extracts were prepared at the indicated times and analyzed by immunoblotting for P-EGFR and E-cadherin. Immunoprecipitation of total EGFR from whole cell extracts of HaCaT or shGal-7 cells obtained after treatment with 100 ng/mL EGF for the indicated times. Immunoblots were probed for ubiquitin and total EGFR. Cropped images are from samples run on the same gels. Full-length blots are displayed in supplementary Fig. S5A. Scale bars stand for standard deviation. (**B**) EGFR Protein stability after EGF treatment. For EGFR stability experiments, HaCaT cells were plated in 6 wells plates, starved overnight then treated with EGF 100 ng/mL and Cycloheximide at 25 µg/mL. After the indicated times, cells were lysed and total EGFR levels were detected by immunoblotting. Cropped images are from samples run on the same gels. Full-length blots are displayed in supplementary Fig. S5B. (**C**) Representative immunofluorescence of EGFR (green) and LAMP-1 (red) in HaCaT cells and in ShGal7 #2 after 30, 60 and 90 min of treatment with 100 ng/mL of EGF. Nucleus are stained in blue (Hoescht) Scale bar = 15 µM.

EGF treatment induces EGFR phosphorylation but also ubiquitination. Indeed signaling receptors are tightly regulated by this posttranslational modification, ubiquitination contributing to receptor endocytosis, sorting, and downregulation^29^. We thus wondered if galectin-7 could influence EGFR ubiquitination. We therefore performed immunoprecipitation experiments on cell lysates from HaCaT and ShGal-7 after EGF treatment during the indicated times, revealing that, as for phosphorylation (Figs. 5A, S5A), total EGFR ubiquitination was more intense in cells deprived for galectin-7 especially after 5 min of stimulation (Figs. 5A, S5A). This led us to suppose that galectin-7 would negatively regulate EGFR ubiquitination. In fact ubiquitination after ligand binding plays a fundamental role both in EGFR endocytosis and intracellular sorting. Indeed, EGFR ubiquitination starts at the plasma membrane and continues along the endocytic pathway. Ubiquitination is also critical at later steps targeting EGFR for degradation through trafficking to lysosomes^30^. Hence, we explored if galectin-7 influences EGFR degradation.

To determine EGFR stability, we checked the level of EGFR at different times of EGF treatment in presence of cycloheximide. Cycloheximide inhibits translation thus the EGFR neo-synthesis that could otherwise mask degradation. We didn’t observe any significant differences in EGFR degradation in our assays between cells depleted or not in galectin-7 (Figs. 5B, S5B). These results have been confirmed by immunofluorescence through co-staining of EGFR and LAMP-1 at 30 mn, 1 h or 1hr30min (Fig. 5C) since it can be observed that in absence of galectin-7 EGFR is not massively targeted to lysosomal compartments. Thus galectin7 doesn’t seem to be a major regulator of EGFR degradation.

### Galectin-7 impacts EGFR trafficking

**Figure 6:**
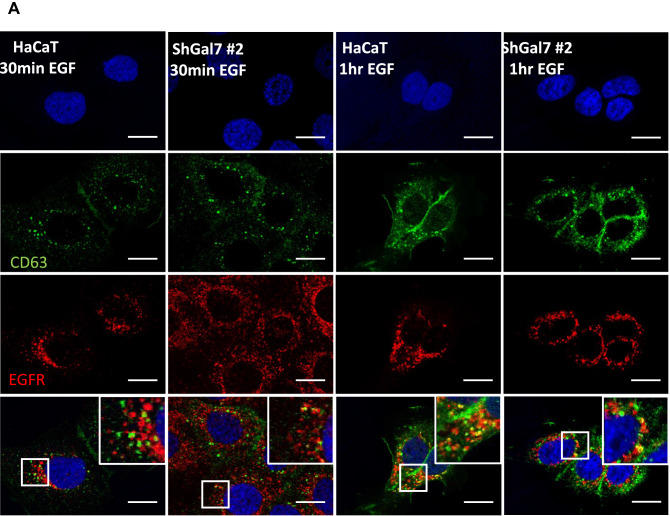

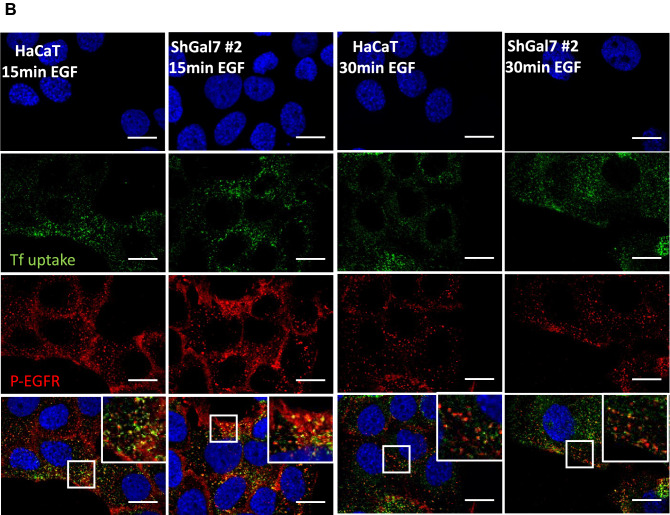

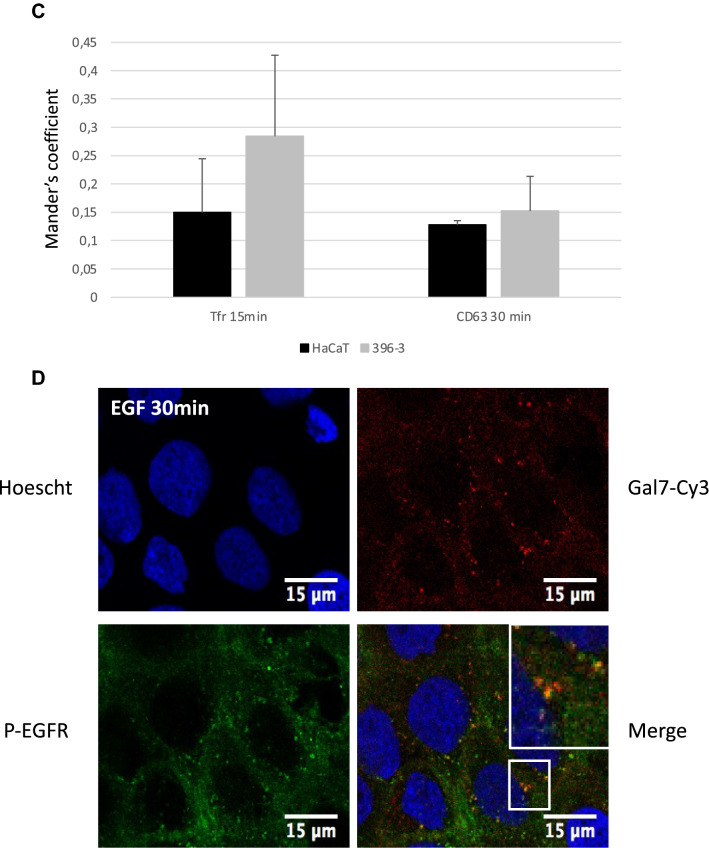
Galectin-7 in endocytosed with EGFR and modulates EGFR endocytosis. (**A**) Representative immunofluorescence of CD63 (green) and EGFR (Red) after 30 min and 1 h of treatment with EGF. Nucleus are stained in blue (Hoescht). (**B**) Representative immunofluorescence of transferrin (green) and P-EGFR (red) in HaCaT cells and in ShGal7 #2 cells at 15 and 30 min after treatment with 100 ng/mL of EGF. Nucleus are stained in blue (Hoescht) Scale bar = 15 µM (**C**) The Mander’s coefficient has been calculated from signal quantifications performed on ImageJ software (Version 2.3.0/1.53f.—http://imagej.net/Contributors) from different samples (n = 3). It corresponds to the ratio between the amount of colocalization signal on the amount of signal of vesicular compartments (respectively Tfr and CD63). (**D**) Representative immunofluorescence of galectin-7 (red) and P-EGFR (green) in HaCaT cells after 30 mn of EGF treatment at 100 ng/mL. Nucleus are stained in blue (Hoescht) Scale bar = 15 µM.

The previous results led us to hypothesize that ubiquitination modification would influence EGFR endocytosis. In order to decipher the consequences of galectin-7 depletion we studied EGFR intracellular trafficking by co-staining with markers of several intracellular compartments. To this purpose, pulse-chase experiments were conducted stimulating cells for 20 min with 100 ng ml^−1^ EGF and after removing unbound ligand chased for 15 min to 1 h. Immunofluorescence staining followed by colocalization analysis establishes the endocytic trafficking of EGF thought the degradative pathway from early (TfR positive) to late (CD63 positive) compartments. At late stages EGFR partially colocalizes with CD63 in both HaCaT cell line and ShGal7 cells indicating that EGFR is able to reach the late endosomal compartments (Fig. 6A). As ubiquitination is also implicated in the early steps of endocytosis and because EGFR degradation is not impacted in cells depleted of galectin-7, we investigated if EGFR could be more efficiently recycled to the plasma membrane. We incubated cells with fluorescent transferrin which uptake allowed us to explore early and recycling endosomes. Our results show a tendency to a higher co-localization in cells lacking galectin-7 suggesting that a high amount of the endocytosed P-EGFR in mutant cells is recycled back to the plasma membrane (Fig. 6B,C). Thus during EGF stimulation, galectin-7 restrains P-EGFR endocytosis and its recycling to the plasma membrane.

Interestingly, as described above in proximity ligation assay, we observed an interaction between galectin-7 and EGFR in cell cytoplasm. To confirm these results we used recombinant galectin-7 coupled to Cy3 (rgal7-Cy3) and performed co-staining with P-EGFR. Strikingly, recombinant galectin-7 was repeatedly observed in endocytosed P-EGFR-containing vesicles (Fig. 6D), indicating that galectin-7 was probably co-endocytosed with P-EGFR. On the contrary, colocalization assays of rgal7 and LAMP-1 gave no signal (data not shown), letting us consider that galectin-7 could travel with P-EGFR only during the early steps of endocytosis. All these results made us consider that galectine-7 exerts a negative control on EGFR endocytosis and recycling.

### Galectin-7 impacts cell migration in absence of EGF

**Figure 7:**
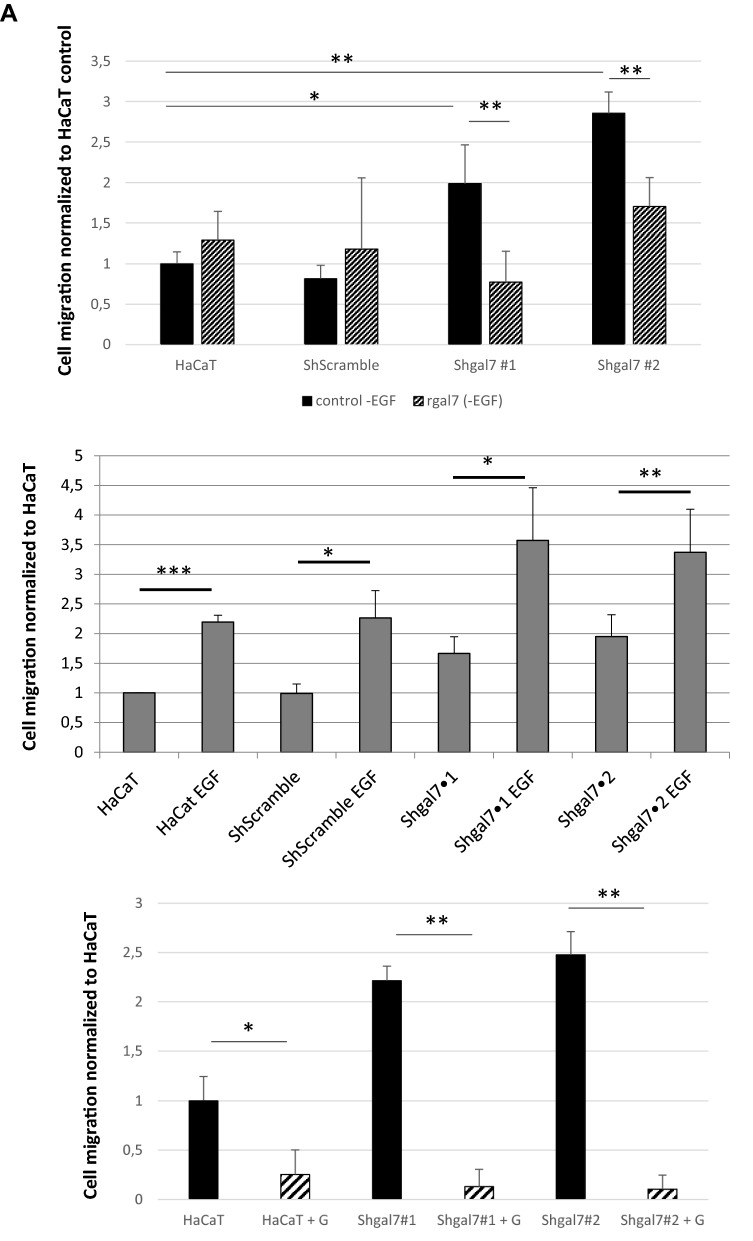

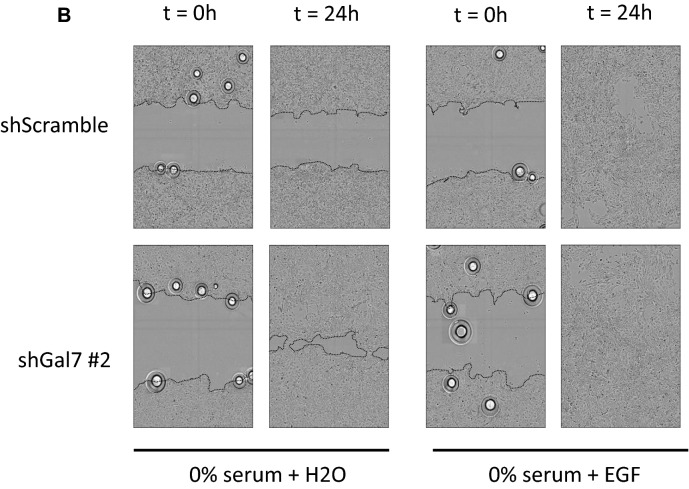
Galectin-7 downregulation enhances cell migration of HaCaT keratinocytes. (**A**) Percentage of wound closure normalized to HaCaT WT cells in insert removal wound healing experiments. Rescue experiments have been conducted with 0.5 mM of recombinant galectin-7. Experiments with gefitinib were conducted with 15 µM of gefitinib. Mean ± s.e.m. are represented (n = 4). (**B**) Images have been extracted from videos of cell migration. Magnification 20 × .

As galectin-7 restrains EGFR phosphorylation and signaling pathway even in absence of added EGF we wondered if galectin-7 could also alter cell motility and cell proliferation. So we set up wound healing assay using insert removal technics^7^ to investigate the migratory potential of galectin-7 depleted clones in cell starvation conditions. Sixteen hours after insert removal, the ShGal7 clones exhibited a significant increase in wound healing capacity of respectively 60% (ShGal7 #1) and 90% (ShGal7 #2) compared to control HaCaT cells under starvation condition (Fig. 7A,B). Interestingly when adding soluble recombinant galectin-7 to ShGal7 clones we can observe that cell migration is similar to control cells (Fig. 7A). Hence this rescue reinforces the hypothesis that galectin-7 also exercises a negative control over cell migration capacity in these conditions. On the contrary, addition of EGF, increases cell migration capacity under all conditions and this capacity is abolished in the presence of gefitinib (Fig. 7A middle and lower panel). These results confirm the role of galectin-7 in restraining unwanted EGFR activation in the absence of EGF.

### Galectin-7 depletion impairs epidermis differentiation

**Figure 8:**
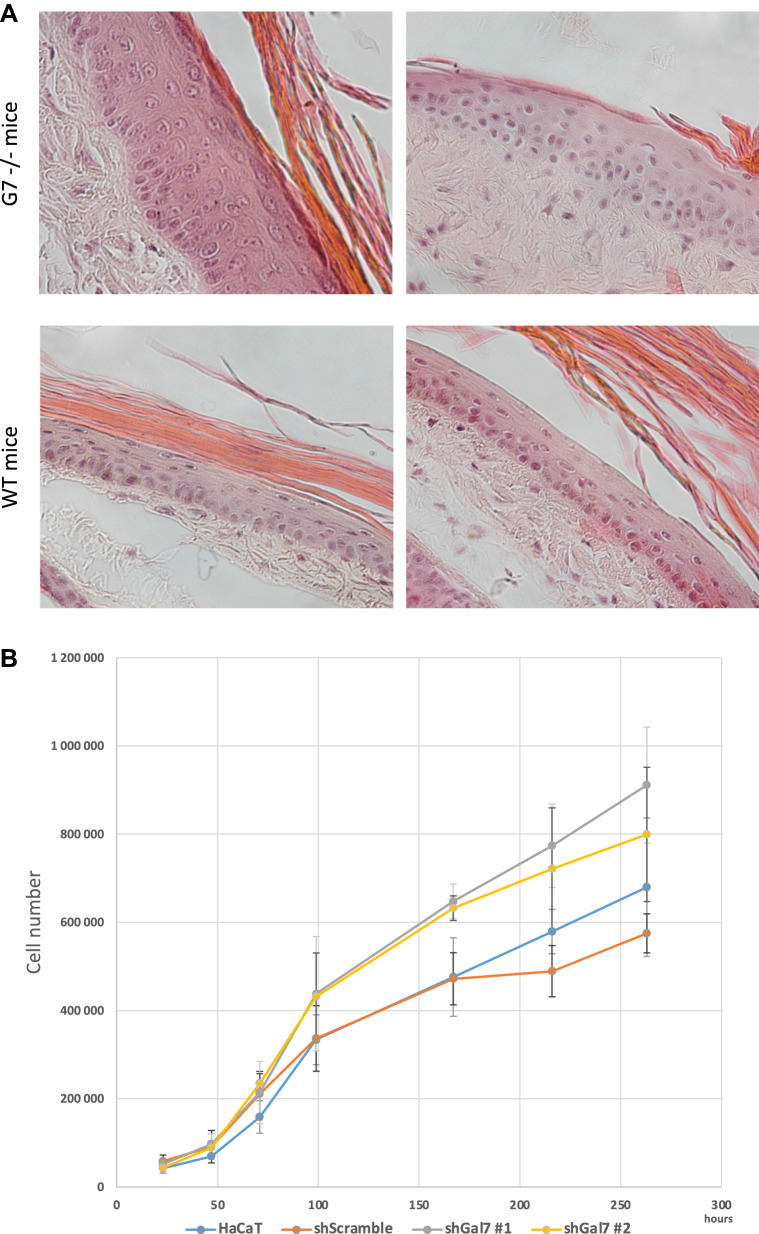

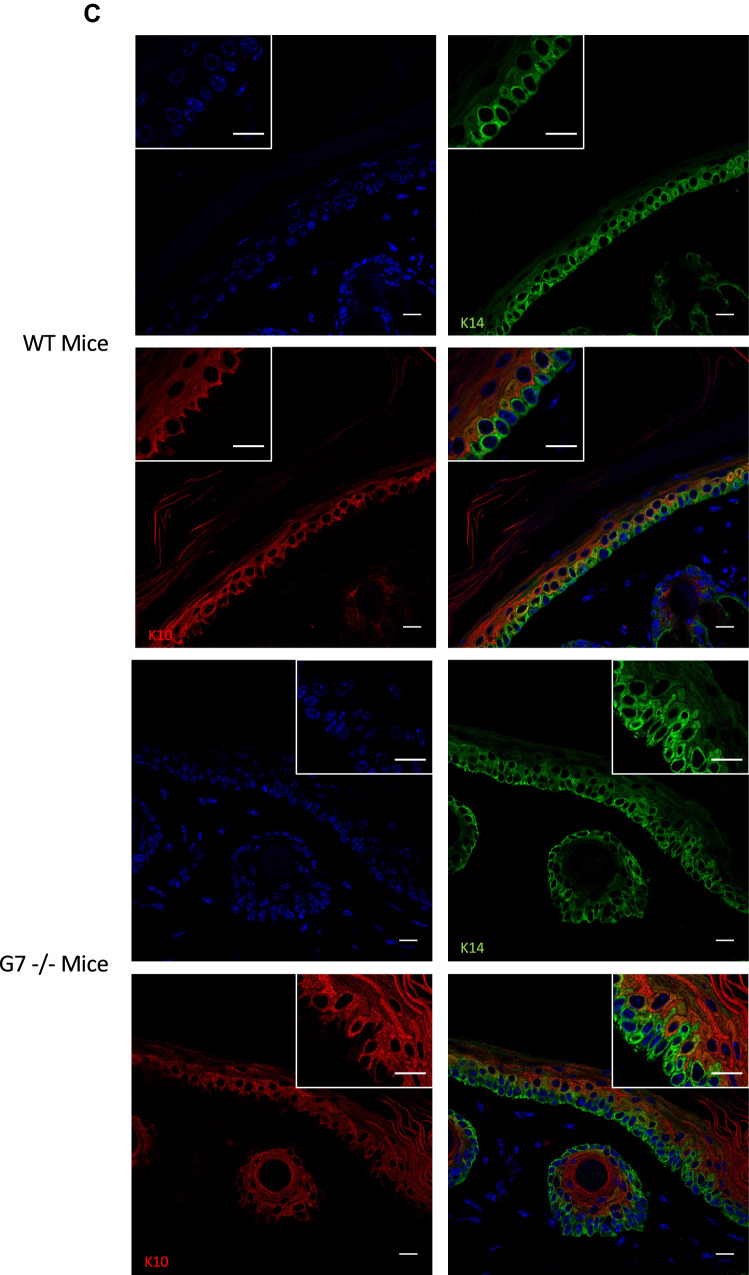

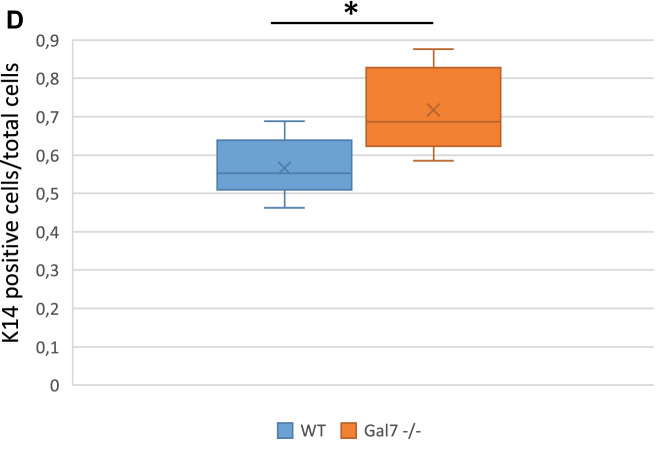
Absence of galectin-7 impairs skin differentiation. (**A**) Representative staining of wild type and Gal7−/− mice tail with Hematoxylin/Eosin. A thickening of the epidermis is well observable in Gal7−/− mice. Magnification 40 × . (**B**) Curves are the results of total cell proliferation assays of HaCaT and shGal7 #2 cell lines counted during 11 days to calculate the mean ± standard deviation (SD). Results are mean of three independent experiments performed in duplicate. (**C**) Representative immunostaining of keratin 14 (green) and keratin 10 (red) in WT and Gal7−/− mice mice tail epidermis showing localization of these two proteins. Scale bar = 15 µm. (**D**) Quantifications of the ratio of K14 positive/total cells performed of immunostaining of keratin 14 and keratin 10. Quantification were performed on ImageJ software (Version 2.3.0/1.53f.—http://imagej.net/Contributors) from different samples from different 2 months old female mice (WT : n = 6 and gal7−/−` : n = 4).

To decipher the impact of galectin-7 depletion in vivo we used galectin-7 null mice generated in our laboratory^8^. Refined observations of tail skin of both wild-type and gal7^−/−^mice revealed the thickening of the epidermis with an accumulation of round cells at the basal layer in absence of galectin-7 (Fig. 8A). To evaluate if this observation could be due to an excess of cell proliferation we performed cell proliferation assay seeding the different cell lines at the same confluence and evaluated cell population size regularly during more than 10 days. A statistical difference is already detectable after 1 week in culture with a higher number of cells counted in both ShGal7 clones compared to the HaCaT cell lines. We can observe on Fig. 8B that HaCaT cells need about an additional 48 h to multiply their population by 3. Thus galectin-7 inhibits growth factor dependent cell proliferation. We therefore studied the distribution of keratin 14 (K14) and keratin 10 (K10) which are respectively markers of basal undifferentiated and differentiated keratinocytes of the epidermis upper layers^2^. Mice deficient for galectin-7 instead of having a single layer of basal cells as observed in wild-type mice exhibit two or even more layers of K14 expressing cells (Fig. 8C) as quantified in Fig. 8D. In addition, a large number of cells with a double K10/K14 labeling can be observed in comparison to the control. To assess these results, we performed RT-qPCR in our cultured cell models unravelling that galectin-7 depletion induces a strong reduction of K10 mRNA expression, while no modification of K14 can be detected (Fig. S6A). Consistently, addition of soluble galectin-7 on HaCaT cells led to the increase of K10 transcription (Fig. S6B).

EGFR is known to be a regulator between cell proliferation and cell differentiation. Indeed, it negatively regulates cell differentiation hence decreasing K10 expression. Relying on our observations in mice we studied the effect of the addition of EGF at 100 ng/ml for 16 h on HaCaT cells and studied the level of K10 transcripts. As expected^4^ EGF induces a significant decrease of K10 transcription in HaCaT cells, having a profile similar to the one observed in absence of galectin-7. Only a non-significant tendency is observed in the cells without galectin-7 probably due to their already weak level of expression (Fig. S6B lower panel). These observations led us to conclude that galectin-7 is involved in keratinocytes differentiation and appears to have an antagonist effect compare to EGFR signaling.

## Discussion

In this study we first pointed out a new tripartite complex between EGFR, galectin-7 and E-cadherin. We also highlighted that galectin-7 not only bridges these two molecules but also regulates both E-cadherin dynamics in response to EGF and EGFR signaling and trafficking. Interestingly, we found that the presence of galectin-7 is essential to maintain minimal levels of EGFR activation in the absence of its ligand.

We and others have previously published that a direct binding of galectin-7 can modulate the function or localization of its bound partners^31^ notably E-cadherin though a sugar-independent binding of its extracellular domain^7^. In the present study, we show that galectin-7 also directly binds to EGFR through its extracellular domain but in a carbohydrate-dependent manner. Herein, we propose an in silico model of galectin-7 making a bond between EGFR and E-cadherin. This robust model leads us to hypothesize that galectin-7 would limit the degree of EGFR freedom. EGFR being otherwise a very flexible molecule galectin-7 probably thus renders its endocytosis less efficient and possibly modifies its interaction with ligands. We also reveal here for the first time that in keratinocytes galectin-7 can bind simultaneously to both membrane proteins, EGFR and E-cadherin, sustaining their cross-regulation at molecular level.

Galectin-7 is known to play a critical role functioning as a regulator of keratinocyte proliferation and migration, as well as maintaining and restoring epidermal homeostasis. Herein we address a new role for galectin-7 in EGFR regulation and function. Indeed, we unravel that impairment of the interaction between EGFR and galectin-7 strongly increases EGFR phosphorylation and its main downstream pathways Akt, Erk and STAT3 inducing their over-activation. Interestingly our results reveal similarities with those obtained by Amaddi et al. on flotillin. However membrane microdomain-associated flotillin proteins knockdown (flotillin-1/reggie-2) results in reduced EGF-induced phosphorylation of EGFR and in reduced activation of the downstream MAPK and Akt signaling^32^. Hence galectin-7 and flotillin could have antagonistic role in the regulation of EGFR signaling pathways.

Signal transducing molecules have been shown to affect membrane trafficking^33^ with important consequences for biological cell outputs. Exposure to stress leads to the removal of the receptor from the cell surface, and this has been proposed to potentiate cell death. Conversely, stress-activated receptor might also be temporary or reversibly removed from the membrane, thereby promoting cell survival and/or proliferation^17^. This cell response to stress must be strongly considered in the case of the regulation by galectin-7 as the expression of galectins has been repeatedly related to stress situation^3,25^. Here we observe that galectin-7 depletion favors EGFR phosphorylation and ubiquitination. These early activation motifs on membrane-based EGFR have been implicated in both endocytosis and degradation during EGF treatment. Nevertheless our results reveal that EGFR doesn’t seem to be more degraded in cells deprived of galectin-7. Thus galectin-7 appears to be implicated in EGFR retention at the plasma membrane. In our experiments, loss of galectin-7 resulted in enhanced EGFR activation and recycling but also as a consequence in increased cell proliferation and migration. Such derailed endocytosis and recycling of cell-surface proteins, including EGFR/RTK, along with disturbed downstream signaling has been implicated in multiple cancers^34^ and is in line with our results on cell proliferation and migration.

Interestingly, it has been published that N-linked glycosylation can define the individual properties of extracellular and membrane-associated proteins and that modifications of these glycosylations can alter constitutive cell proliferation and trigger epithelial-to-mesenchymal transition^19^. The authors suggest that the allosteric organization of EGFR Tyrosine Kinase is dependent on extracellular N-glycosylation events and that EGFR functions would be linked to the N-glycosylation status of EGFR. Hence aberrant glycosylation would favor the constitutive activation of EGFR, conducting to proliferation and invasiveness. Thus the key role of glycosylation motifs in the control of endocytic pathways of decorated membrane protein has been recently reviewed with a special emphasis on the control of this phenomenon by galectins^35^. This role can be strengthened at the light of our results. Indeed, our model supports the hypothesis that pathologic glycosylation of EGFR extracellular domain impairing galectin-7 binding would disrupt its control on proliferation and migration. These effects on migration can be related to previous results revealing that galectin-7 appeared to promote cell migration^7,8^. It is important to note, however, that the difference between these results and the present results may be due to the presence or absence of serum or even to the fact that the observations were made in vivo where a range of growth factors including EGF are present.

In vitro studies show that activation of EGFR plays an important role in re-epithelialization by increasing keratinocyte proliferation and cell migration in acute wounds^36^. Furthermore, it has been recently shown that E-cadherin is a master regulator of junctional and cytoskeletal tissue polarity in stratifying epithelia regulating the suprabasal localization and activation status of the EGFR essential to facilitate the development of a functional epidermal barrier^37^. To fulfill our study, in vivo observations in galectin7−/− mice compared to wild-type mice pointed out defects in epidermis differentiation in absence of galectin-7, hence revealing a thickening of the epidermis accompanied by an accumulation of K14 positive cells. In accordance with these data from the literature and our own results, we hypothesized that galectin-7 would control this differentiating step by regulating E-cadherin and EGFR functions thus preventing excessive proliferation, migration and therefore tumorigenic development. It has also been shown that an excess of galectin-7 has an impact on the epidermis, being associated to its thickening^8^. Hence, galectin would act as a regulator whose precise dosage is required at the tissue level.

Galectin-7 down-regulation in stratified epithelia has been reported to be associated with the development of several cutaneous manifestations and disorders and even to esophageal dysfunction in systemic sclerosis patient^38^. As EGFR and E-cadherin are closely related and regulated, we predicted a mechanism in which galectin-7 would be a major actor of epidermis differentiation through the cross-regulation of EGFR and E-cadherin. These results provide unique mechanistic insights into how EGFR and E-cadherin would be interacting which is essential for the understanding of epithelial homeostasis and the development of anti-cancer strategies.

## Experimental procedures

### Cell culture

The HaCaT cell line (Human adult low Calcium high Temperature) were grown in Dulbecco’s Modified Eagle Media (DMEM, Invitrogen) supplemented with 2 mM essential amino acids (Invitrogen), 10 units.ml^−1^ penicillin, 10 μg.ml^−1^ streptomycin (Invitrogen) and 10% foetal bovine serum (FBS) in a 5% CO2 atmosphere at 37 °C. Two independent clones with reduced expression of galectin-7 were generated by stable expression of shRNAs as previously described^7^.

### Animals

Mice were kept on a C57Bl/6 background and housed in a specific pathogen-free animal facility. All experiments were performed on 2 months-old female mice. Animals were handled respecting the French regulations for animal care and wellness, and the Animal Experimentation Ethical Committee Buffon (CEEA-40) approved all mice work. We confirm that the authors complied with the ARRIVE guidelines.

### Histology and immunostaining

Tissue processing and immunostaining were performed as previously  described^7^. The primary antibodies used are described in supplementary materials.

Nuclei were stained with Hoechst33342 (H3570, Invitrogen) and confocal acquisition was performed using a Leica SP5 microscope. Quantifications were realized with the ImageJ software (Version 2.3.0/1.53f.—http://imagej.net/Contributors).

### Immunofluorescence

Cells were washed with PBS and fixed for 20 min in paraformaldehyde (PFA) 4% at room temperature before being permeabilized for 20 min in PBS—0.025% saponin and then blocked for 30 min in PBS—0.025% saponin—1% BSA (Bovine Serum Albumin, Sigma-Aldrich). Cells were incubated overnight at 4 °C in PBS—0.025% saponin, 1% BSA containing the primary antibody. The following day, cells were incubated at room temperature in PBS—0.025% saponin—1% BSA containing the secondary antibody coupled to a fluorochrome for 1 h protected from light. The nuclei were stained with 10 μg.ml − 1 Hoechst33342 (H357C, Invitrogen). Coverslips were mounted on slides with Fluoromouont-G (0100–01, Southern Biotech) and visualized using an SP5 confocal scanning Tandem RS (Leica) and analyzed by ImageJ software (Version 2.3.0/1.53f.—http://imagej.net/Contributors). In order to quantify the colocalization rate, the Mander coefficient was used with the ImageJ software (Version 2.3.0/1.53f.—http://imagej.net/Contributors). The threshold values were all adjusted to the same value and then the area of the different images corresponding to the three signals were calculated. At least 3 independent experiments have been conducted. Antibodies are detailed in supplementary materials.

### Immuno- and co-immunoprecipitations

Experiments were performed as previously described^9^. Whole cell extracts were prepared from confluent cells grown on 10 cm tissue culture dishes. For the detection of EGFR ubiquitination, total EGFR Immunoprecipitation was performed with 600 μg of proteins from whole cell extracts of HaCaT or shGal-7 cells pretreated with 100 ng.ml − 1 EGF for the indicated times. The negative control was carried out from a lysate of untreated HaCaT cells. Ubiquitin was revealed with an antibody from Santa Cruz (sc8017, dilution 1/1000).

### In vitro binding assay

0.3 M of purified proteins (recombinant human E-cadherin 8505 and recombinant human EGFR 344-ER R&D system) were mixed and incubated in 100 μl of lysis buffer (25 mM TrisHCl pH 7.5, 100 mM CaCl2, 1 mM EDTA, 1 mM EGTA, 0.5% NP40, 1% TritonX-100, and a protease inhibitor cocktail (11836145001, Roche)) overnight at 4 °C under agitation. Then, 60 μl of protein-A-Sepharose (P9424, Sigma) was added and samples were incubated 3 h at 4 °C under agitation. Samples were washed twice with PBS—0.5% NP40 and twice with PBS before resuspension in Laemmli buffer.

### Western blot

Proteins (30–50 μg of total cell lysates) were separated in SDS-PAGE gels and transferred to PVDF membranes (Amersham Hybond-P, GE Healthcare). Membranes were then blocked in PBS-T (PBS—0.1% Tween 20) supplemented with 5% non-fat milk for 1 h at RT and incubated with the primary antibody overnight at 4 °C. Immunoblots were visualized using a horseradish peroxidase-conjugated secondary antibody followed by enhanced chemiluminescence detection with an ImageQuant LAS 4000 developer (GE Healthcare).

### Proximity ligation assay

The assay was performed with the Duolink in Situ Red Starter kit from Sigma-Aldrich according to the manufacturer’s instructions.

### In vitro wound healing assay

Cells were plated in 12-well plates on both sides of a plexiglass insert that was removed once the cells had reached confluence (T0) and cell culture medium was replaced by serum-free medium. Cells in the entire well were imaged at T0 and T16 (T = 16 h) and the wound closure was calculated by the difference of the covered area by the cell monolayer between T0 and T16. Images were taken with a Leica MZFLIII system through an Axiocam HRc from Zeiss. Results are mean of three independent experiments performed in triplicate.

### Antibody uptake experiments

Cells were incubated in fresh growth medium containing the anti-E cadherin antibody on ice or at 37 °C for different periods of time. Surface-bound antibodies were removed by 1 wash cold PBS then 3 × 5 min acid washes (0.5 M acetic acid, 0.5 M NaCl in PBS) under agitation on ice. Cells were washed with ice-cold PBS +  + (PBS + 1 mM CaCl2 + 0.5 mM MgCl2), then fixed in 4% paraformaldehyde for 20 min at room temperature and processed for immunofluorescence. The images for quantification were taken with a DMRA2 Leica Microscope. For quantification, the images were background subtracted and cellular regions were identified to measure the total fluorescence intensity using the ImageJ software (Version 2.3.0/1.53f.—http://imagej.net/Contributors). Fluorescence intensity measured from cells incubated with E-cadherin antibody 1 h on ice were averaged and subtracted from the values measured for the corresponding clones. For each condition tested, three independent experiments were performed and approximately 15 cell groups were analyzed per experiment.

### Cell proliferation assay

Cells were seeded at a density of 5.10^4^ cells per well of 24-well plates and cultured for 2 weeks renewing the medium 3 times a week. Two wells of plated cells per each condition were trypsinized each day the first week and every other day the second week. Total cell numbers were counted in a Malassez cell and expressed as the mean ± standard deviation (SD). Cell number was graphed to obtain the growth curves. Results are mean of three independent experiments performed in duplicate.

### E-cadherin modeling

We modeled the five extracellular domains of human E-cadherin using the mouse structure as template (PDB ID: 3Q2V). Alignment between human and mouse sequences were performed using ORION^39,40^. The percentages of sequence identity and coverage obtained are of 60.8% and 82.3% respectively. The ORION alignment has then been used by MODELLER^41^, resulting in a high-quality model with a DOPE Z-score = − 4.27. Z-scores represent the number of standard deviations from the mean of a distribution (generally random) of a given value. The highest the absolute value, the better. Using the same protocol and the same template, we modeled the third domain of E-cadherin extracellular region which has a sequence identity of 76.7% with the template with a coverage of 100%. The resulting model has a DOPE Z-score of − 2.5.

### In silico study of E-cadherin/Galectin-7 interaction

The study of interaction between human E-cadherin model and galectin-7 crystal structure (PDB ID: 1BKZ) was performed through docking simulations using MEGADOCK 4)^42^. 54,000 poses were generated with 3 predictions per each rotation and default scoring function, resulting in more than 162,000 docking experiments.

To model the interaction of galectin-7 with 2 E-cadherin molecules, the first E-cadherin was blocked to avoid E-cadherin/E-cadherin interactions. Galectin-7 is a highly symmetrical homodimer except for the seven first residues of the N-ter extremity which are in open conformation in chain B and close conformation in chain A. Because these residues are important for E-cadherin/galectin-7 interaction and are inherently flexible, we replaced chain A by a duplication of chain B. Expect for this small region both chains are very close to each other (global RMSD of 0.3 Å). Each E-cadherin molecule forms the same contacts with galectin-7, resulting in a set of consensus residues that might be responsible for the interaction.

### Modeling of full E-cadherin/Galectin-7 complex

Construction of the full E-cadherin-galectin-7 complex has been built using the galectin-7 docked with two copies of E-cadherin ectodomain 3. E-cadherin model of the five ectodomains (PDB: 3QV2) have then been aligned on both copies of ectodomain 3, resulting in a galectin-7 molecule interacting with two complete E-cadherin. Finally, the full complex has been manually positioned to membrane model containing phosphatidylcholines and phosphatidylserines. Lipids lipid content follow the protocol of Arkhipov et al.^28^: 30% POPS and 70% POPC in intracellular side and 100% POPC in extracellular side.

### Statistics

The mean and standard deviation were graphed for each sample and* P*- values reported as follow * * P* ≤ 0.05, ** * P* ≤ 0.01, *** * P* ≤ 0.001.

Statistical significance was determined using ANOVA tests when no other precision is indicated in the legend of the figure. The standard error from at least 3 independent biological replicates is plotted as shown with reported p-values comparing highlighted samples. In all cases a p-value of less than 0.05 is considered to be significant. Statistical analysis was performed using a combination of AnaStats and Excel softwares.

## Supplementary Information

Supplementary Information 1.

Supplementary Information 2.
