# Supplementary material for: Identification of a new regulation pathway of EGFR and E-cadherin dynamics
Source: Sci Rep. 2021 Nov 22;11:22705. doi: 10.1038/s41598-021-02042-3 (PMC8609017; doi:10.1038/s41598-021-02042-3)
Supplement: Supplementary file 1 — Supplementary Information 1. [file 41598_2021_2042_MOESM1_ESM.pdf]

Identification of a new regulation pathway of EGFR and E-cadherin dynamics

Veronique Proux-Gillardeaux[1], Tamara Advedissian[2], Charlotte Perin[3,4], Jean-Christophe Gelly[3,4], Mireille Viguier[1] and Frederique Deshayes[1*]

Figure S1A

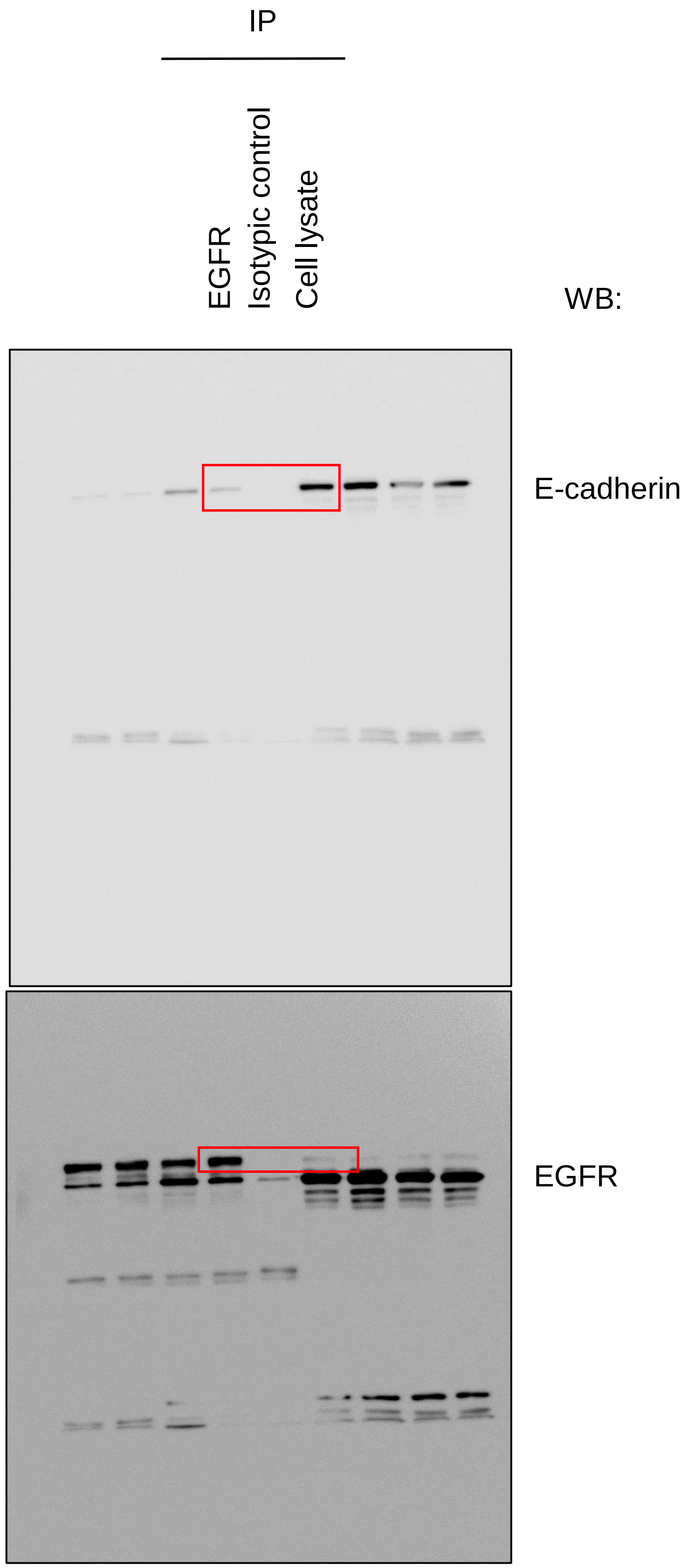

Figure S1B

Beads	+	+	+
E-Cad-His	+	+	+
EGFR-Fc	+	+	-
rGal7	+	-	+
rGal7R74S	-	+	-

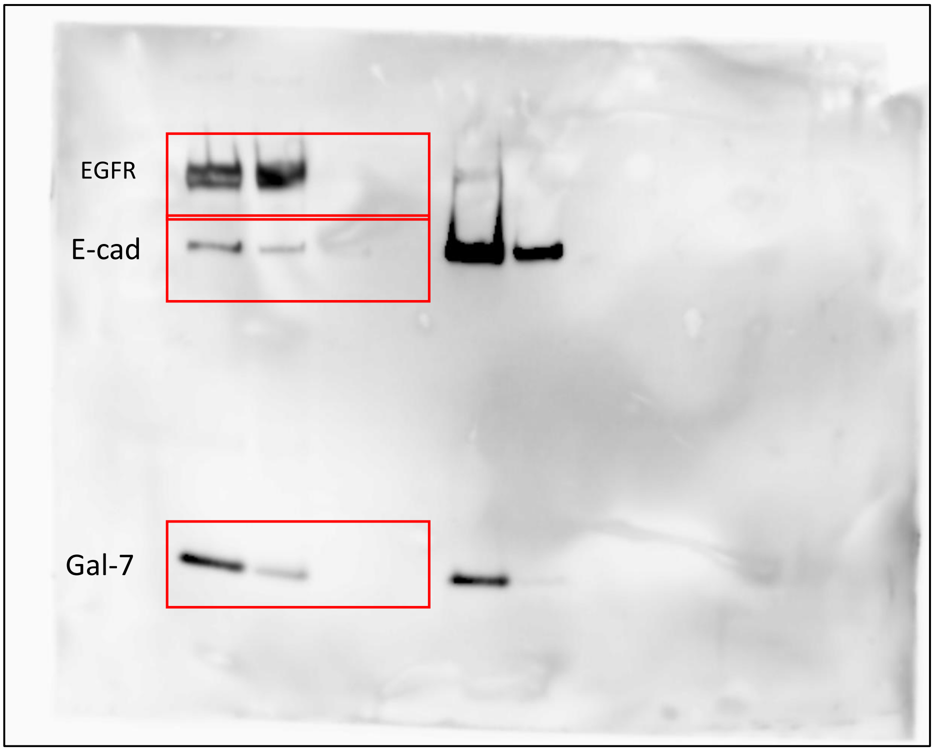

Figure S1B

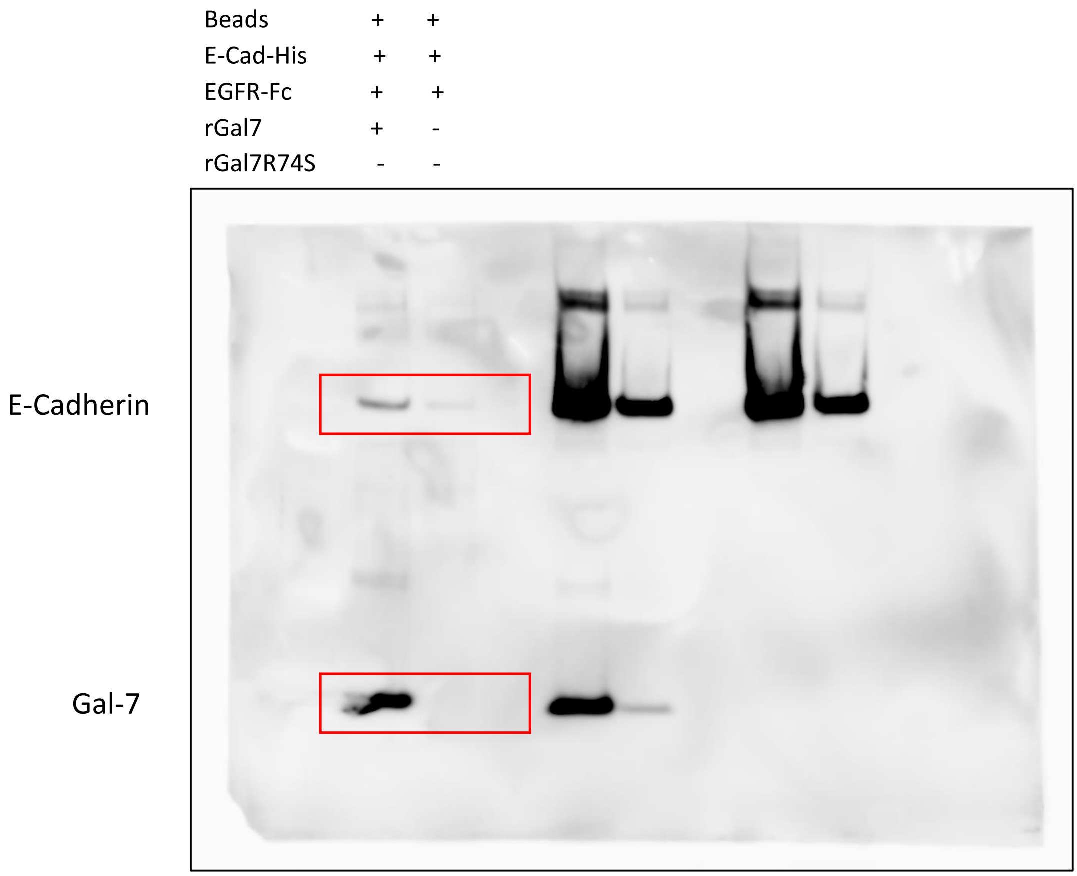

Figure S1B

Beads	+	+
E-Cad-His	+	+
EGFR-Fc	+	+
rGal7	+	-
rGal7R74S	-	-

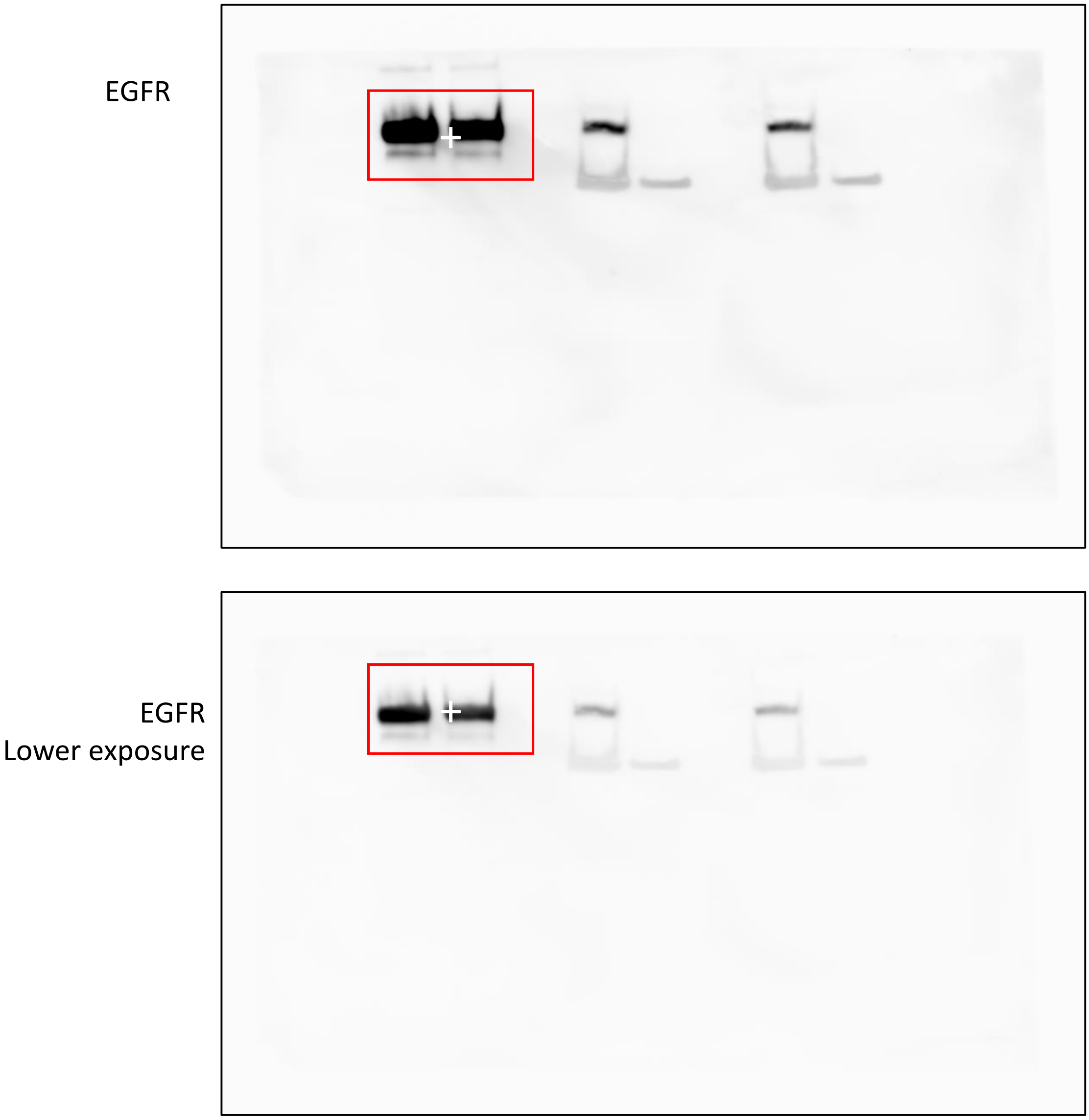

Figure S1: A/E-cadherin and EGFR interact in HaCaT cells. Co-immunoprecipitation experiments were realized with cell extract of a confluent cell monolayer and indicate that E-cadherin and EGFR interact in HaCaT cells. IP were done either with EGFR, Galectin-7 or control IgG respectively. Image shown are representative of three independent experiments. B/Full-length blots presented in Fig. 1B.

Figure S2A

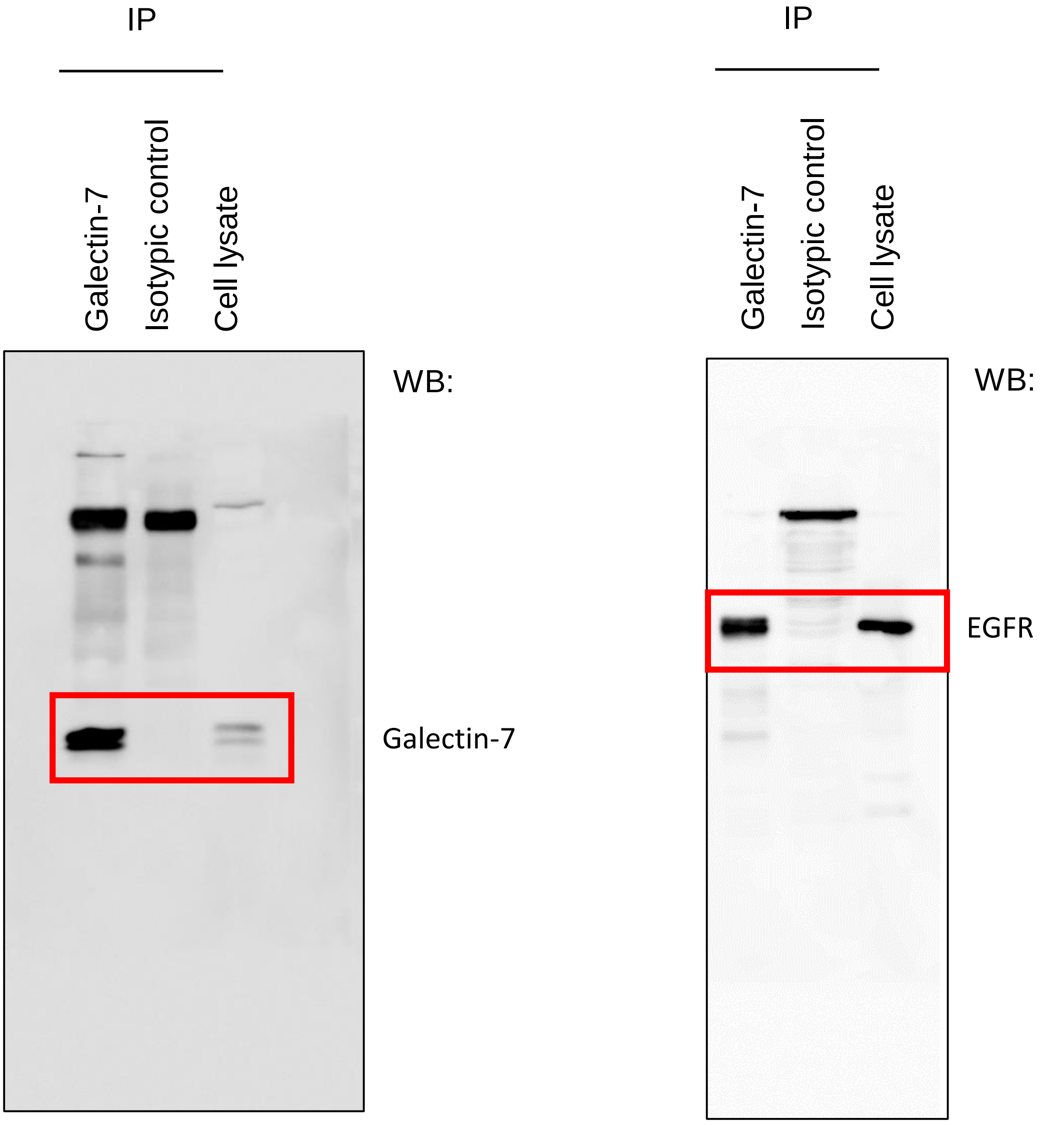
IP
Galectin-7
Isotypic control
Cell lysate
WB:
Galectin-7
IP
Galectin-7
Isotypic control
Cell lysate
WB:
EGFR

Figure S2A

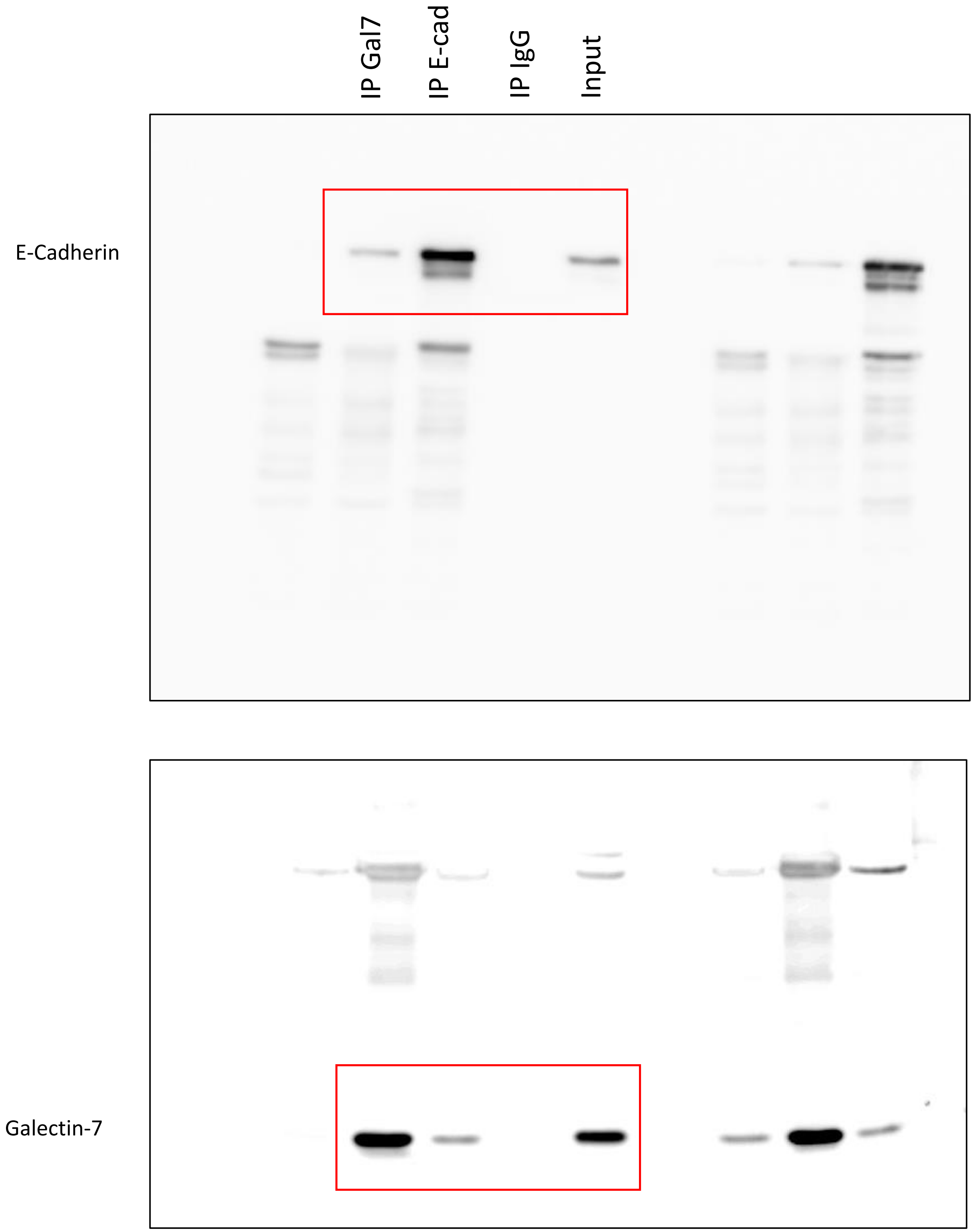

Figure S2B

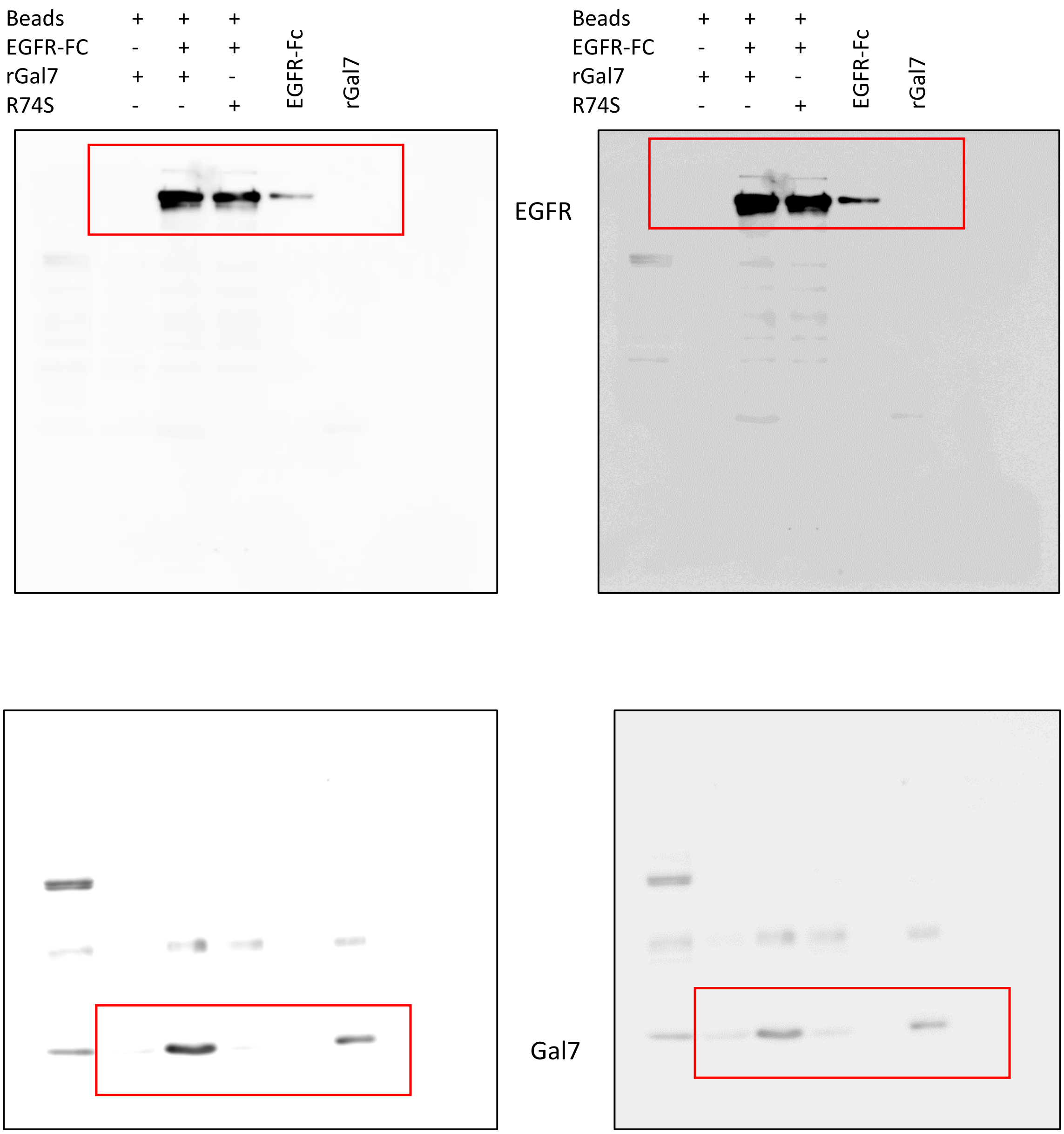

Figure S2: A/Full-length blots presented in Fig. 2A. B/ Full-length blots presented in Fig. 2B.

Figure S3A

a

```
EC3      NTPAWEAVY------TILNDDGG--QFVVTTNP
         |...|...|      |.:.|:||  .||....|
Bcl-2    NIALWMTEYLNRHLHTWIQDNGGWDAFVELYGP
```

b

```
EC4      ITSYTAQ-EPDTFMEQKITYRIWRDTANWLEINP--DTGAISTRAELDRE
         :|.:||: ...|.:|:     ::||..||..|..  :.|.:.....::||
Bcl-2    LTPFTARGRFATVVEE-----LFRDGVNWGRIVAFFEFGGVMCVESVNRE
```

Figure S3B

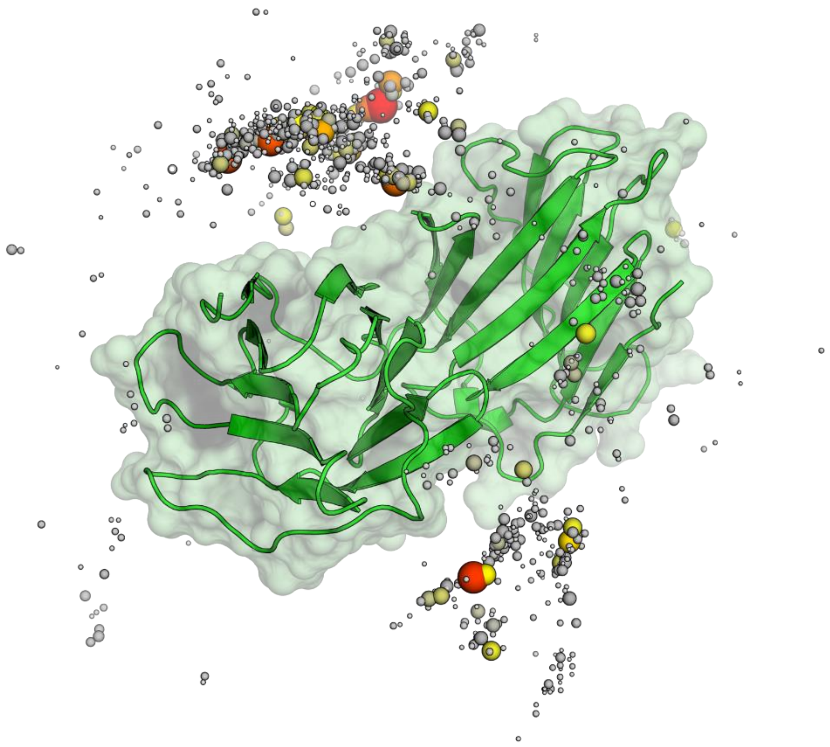

Figure S3C

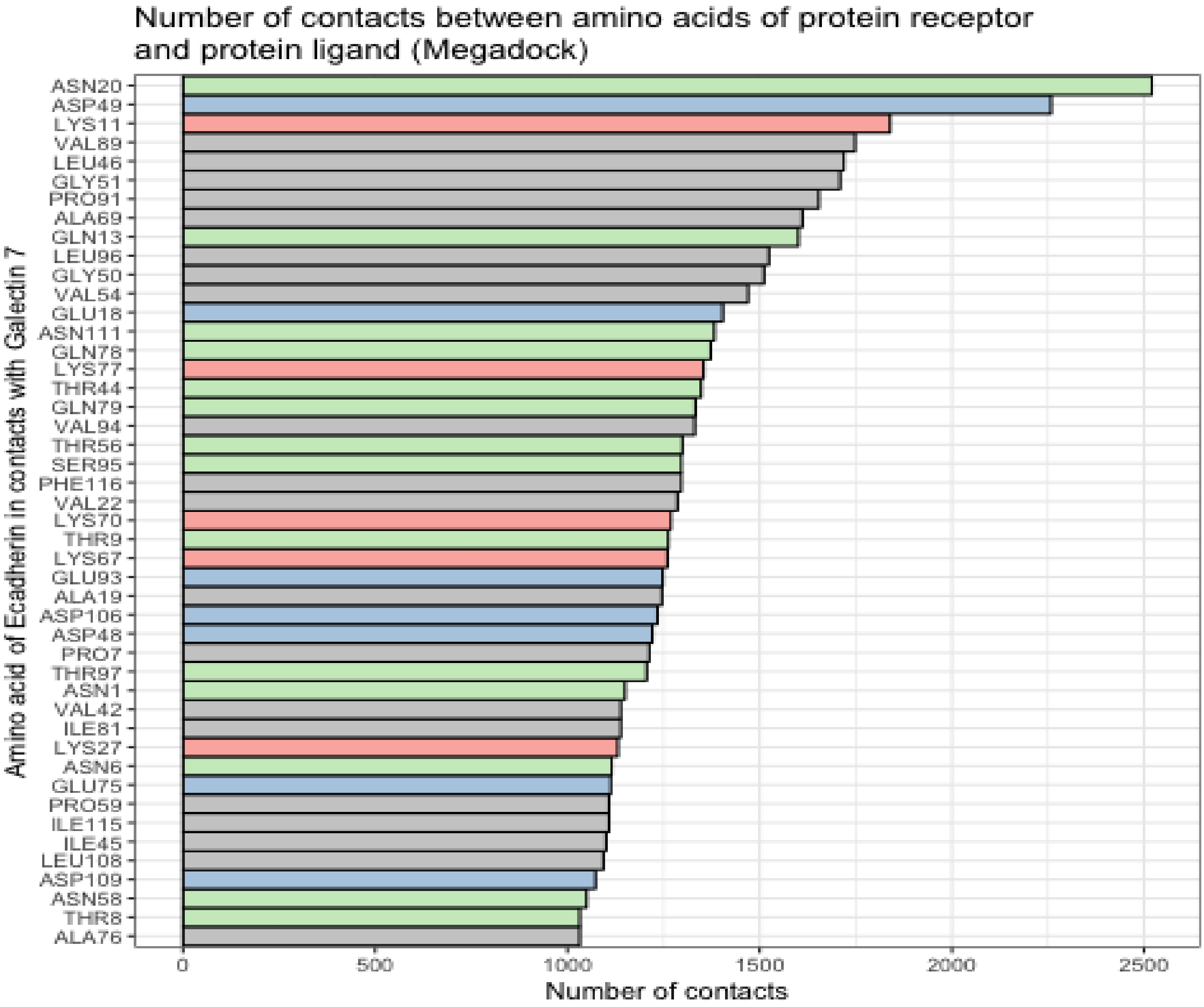

Number of contacts between amino acids of protein receptor and protein ligand (Megadock)
ASN20
ASP49
LYS11
VAL89
LEU46
GLY51
PRO91
ALA69
GLN13
LEU96
GLY50
VAL54
GLU18
ASN111
GLN78
LYS77
THR44
GLN79
VAL94
THR56
SER95
PHE116
VAL22
LYS70
THR9
LYS67
GLU93
ALA19
ASP106
ASP48
PRO7
THR97
ASN1
VAL42
ILE81
LYS27
ASN6
GLU75
PRO59
ILE115
ILE45
LEU108
ASP109
ASN58
THR8
ALA76
Amino acid of Ecadherin in contacts with Galectin 7
0
500
1000
1500
2000
2500
Number of contacts

Figure S3D

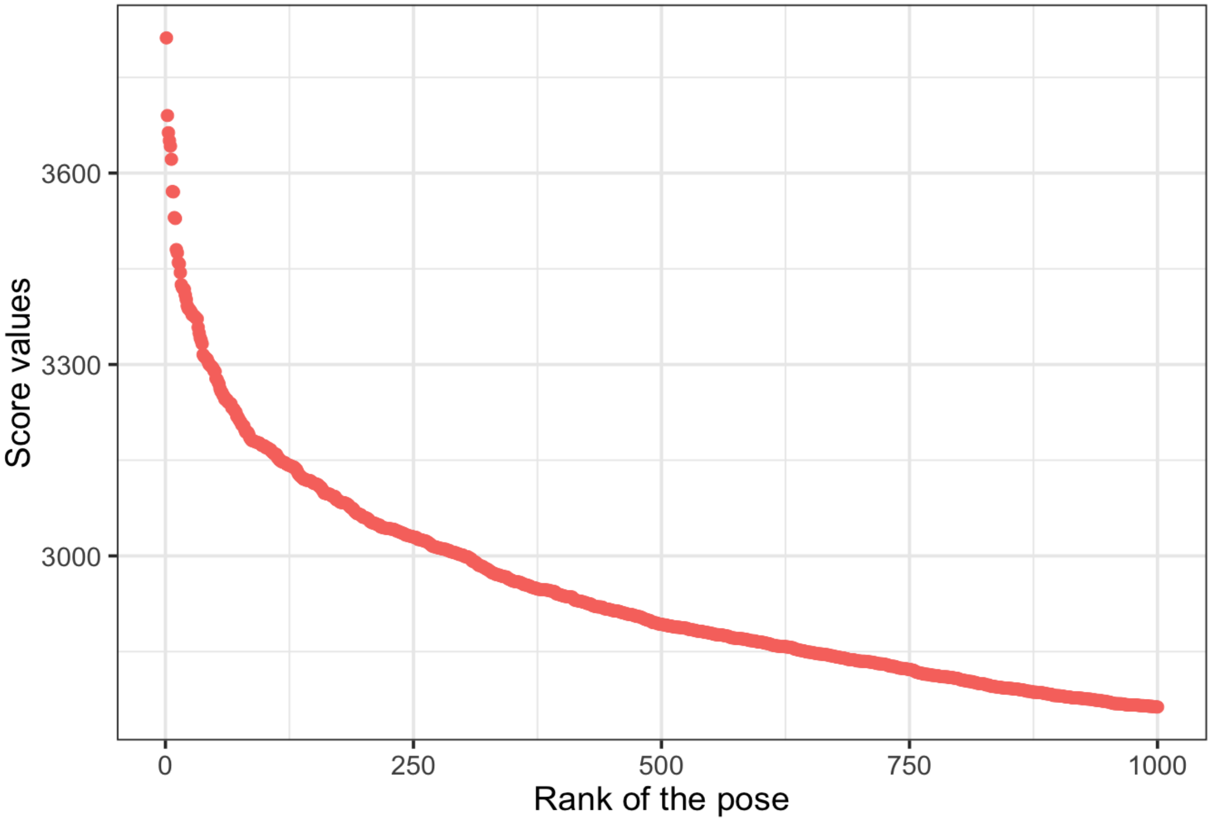

Figure S3: A/ Local Smith & Waterman alignments between Bcl-2 and E-cadherin a) ectodomain 3 (EC3) et b) ectodomain 4 (EC4). B/ Centers of gravity (as spheres) of E-cadherin top 1.000 docking poses around galectin-7 (in cartoon). Diameter and color of the spheres are proportional to docking energies. C/ Number of times an E-cadherin ectodomain 3 residue is found at the interface with galectin-7. D/ Scores computed by MEGADOCK default scoring function for the top 1.000 poses.

figure S4A

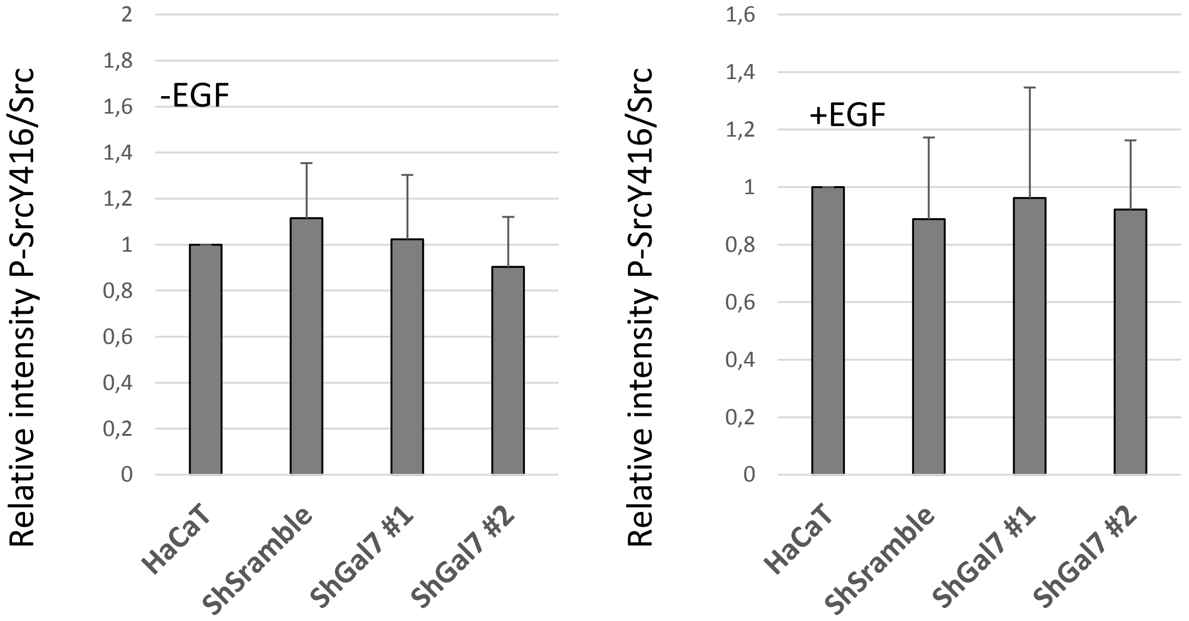

-EGF
Relative intensity P-SrcY416/Src
2
1,8
1,6
1,4
1,2
1
0,8
0,6
0,4
0,2
0
HaCaT
ShSramble
ShGal7 #1
ShGal7 #2
+EGF
Relative intensity P-SrcY416/Src
1,6
1,4
1,2
1
0,8
0,6
0,4
0,2
0
HaCaT
ShSramble
ShGal7 #1
ShGal7 #2

figure S4A

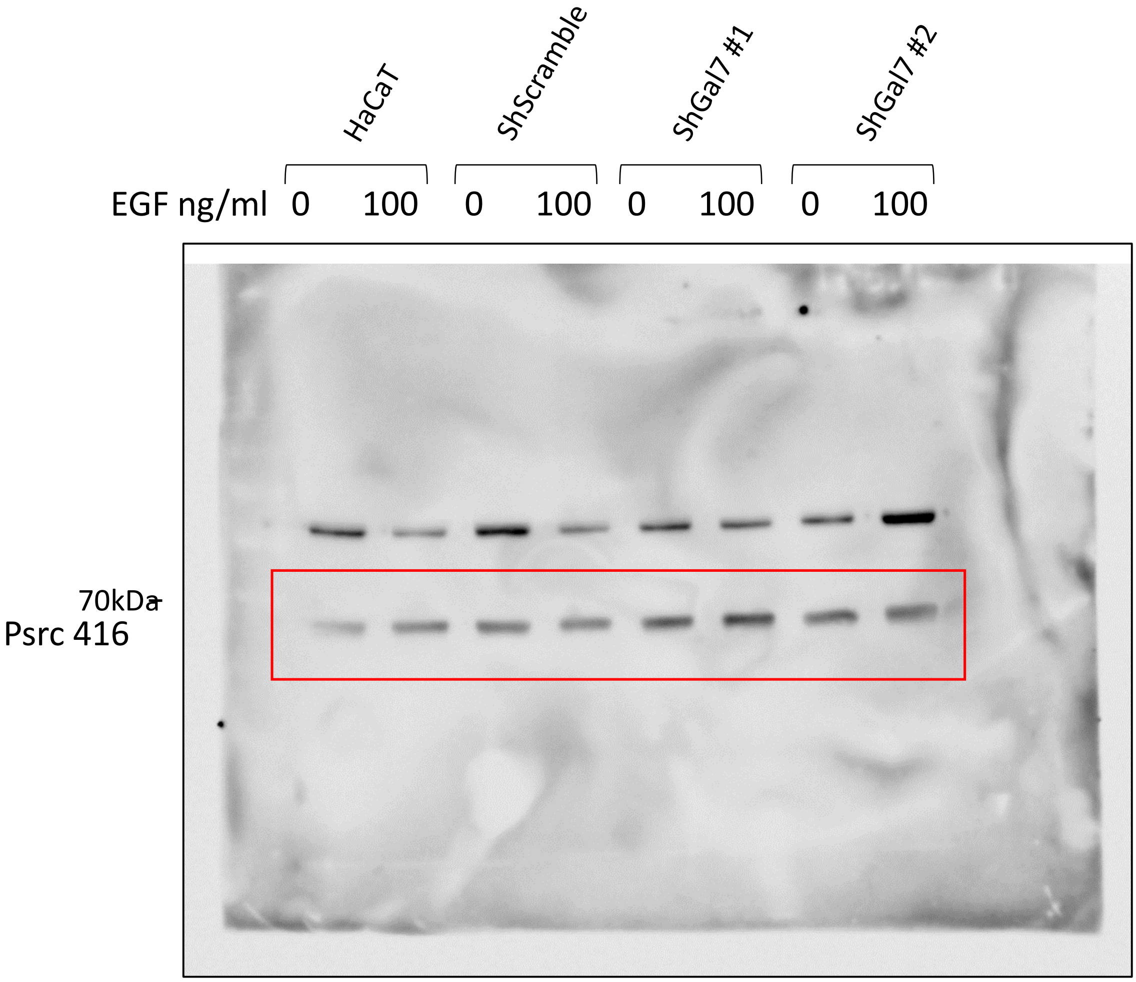

HaCaT
ShScramble
ShGal7 #1
ShGal7 #2
EGF ng/ml
0
100
0
100
0
100
0
100
70kDa
Psrc 416

figure S4A

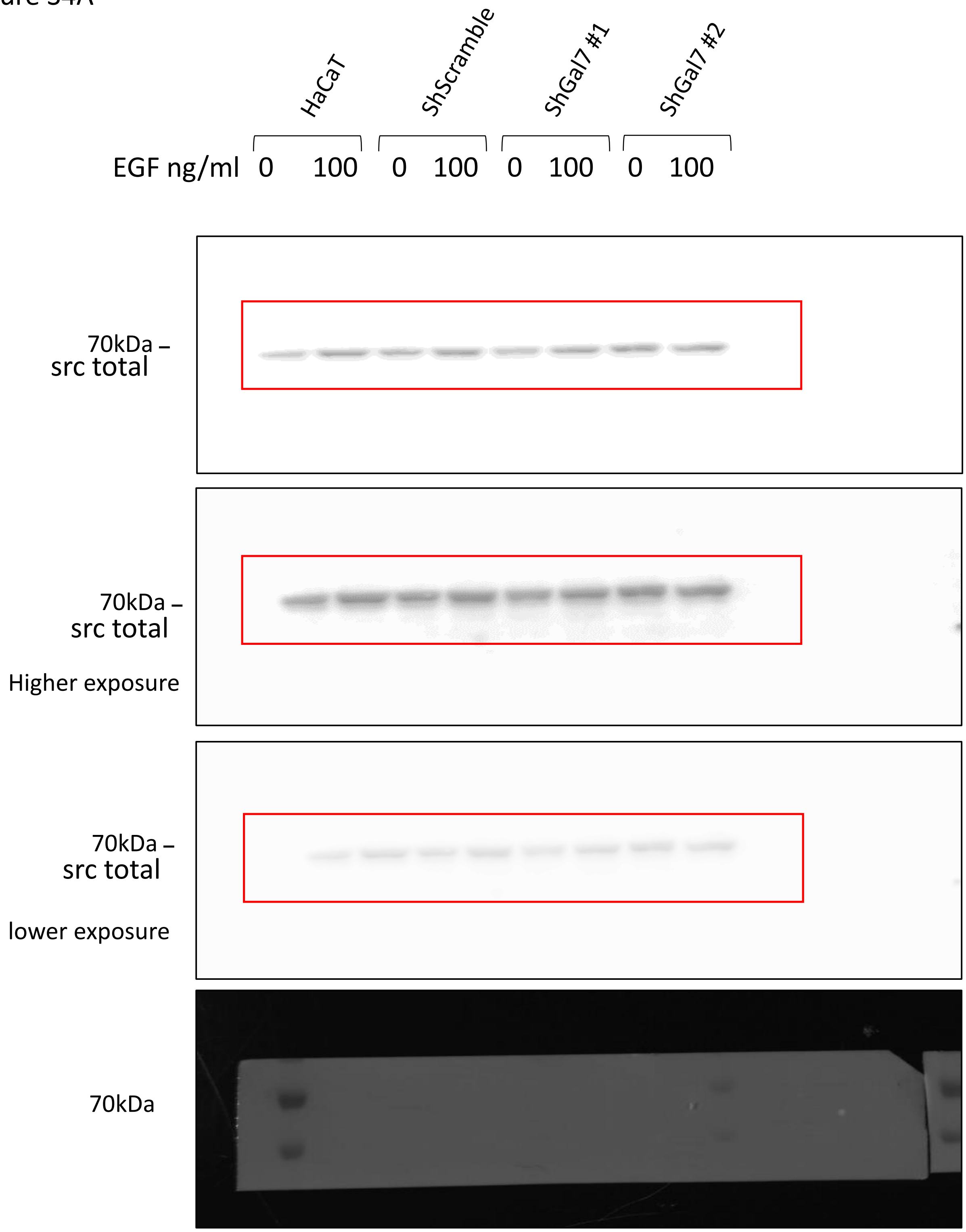

visible light membrane with molecular weight

figure S4A

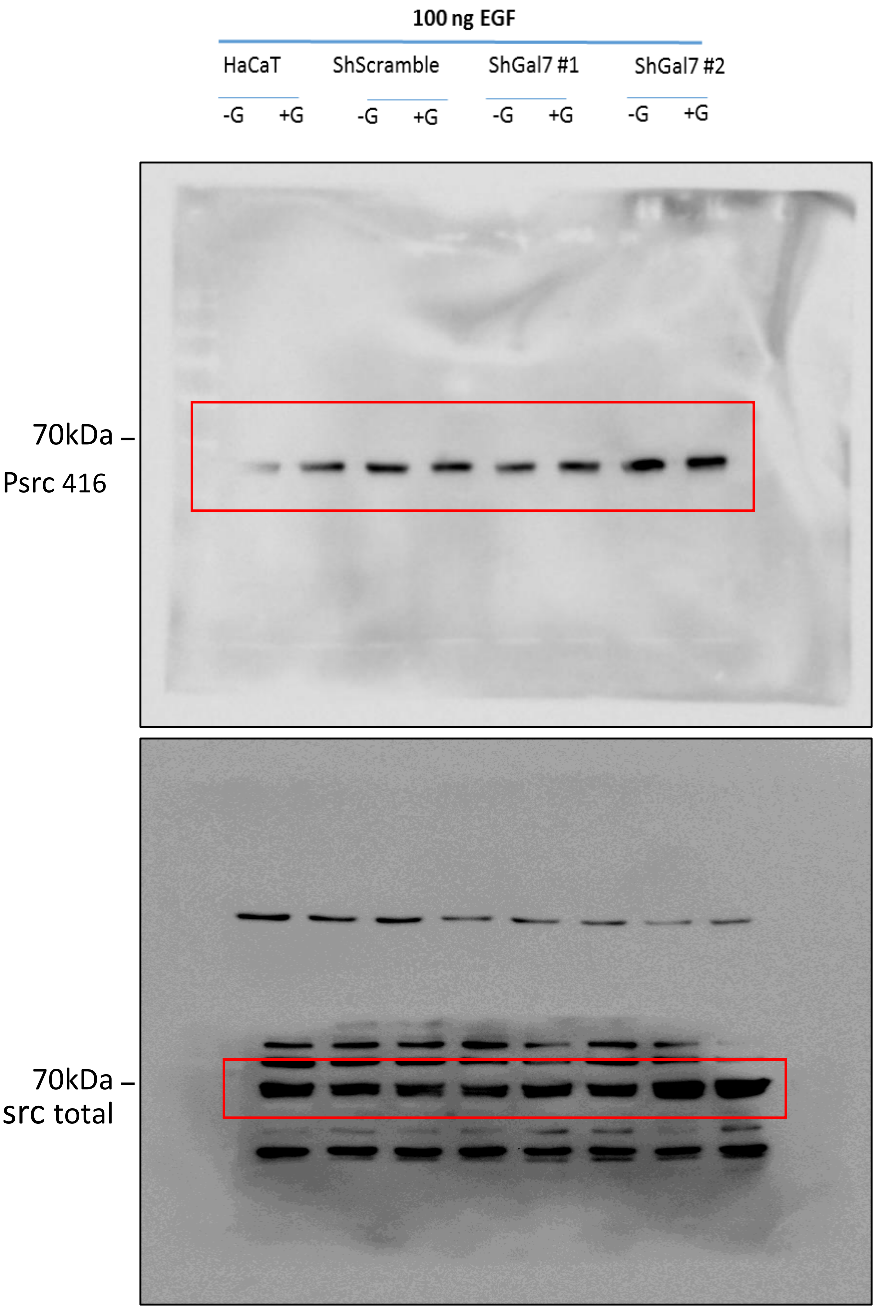
100 ng EGF
HaCaT
ShScramble
ShGal7 #1
ShGal7 #2
-G +G -G +G -G +G -G +G
70kDa –
Psrc 416
70kDa –
src total

figure S4A

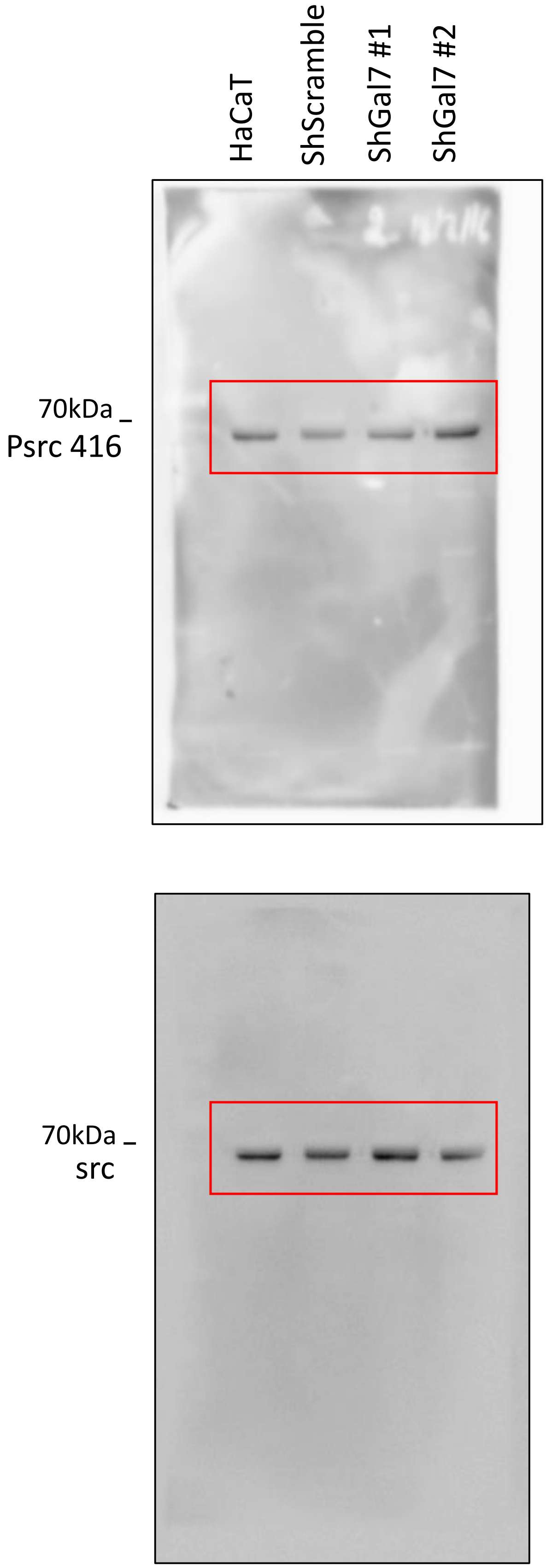

figure S4B

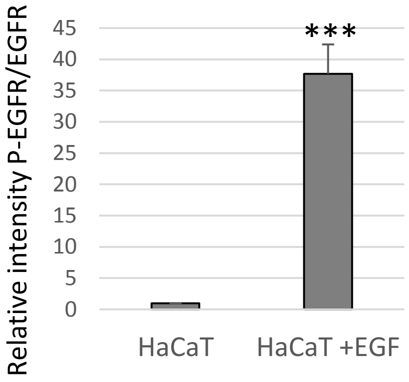

figure S4C

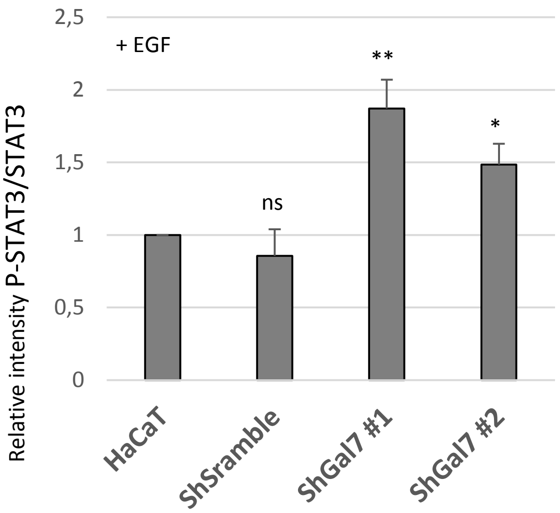

figure S4C

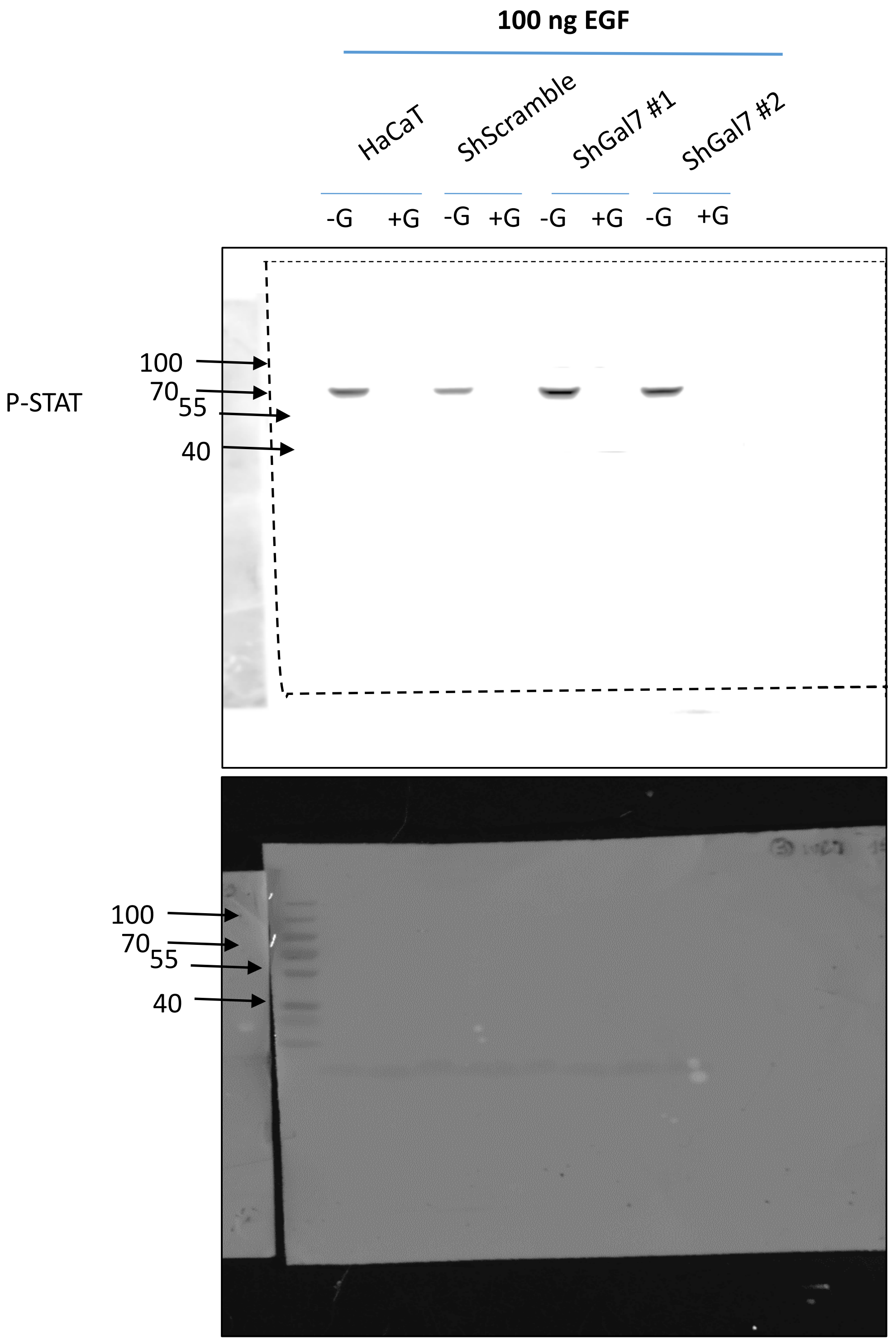

visible light membrane with molecular weight

figure S4C

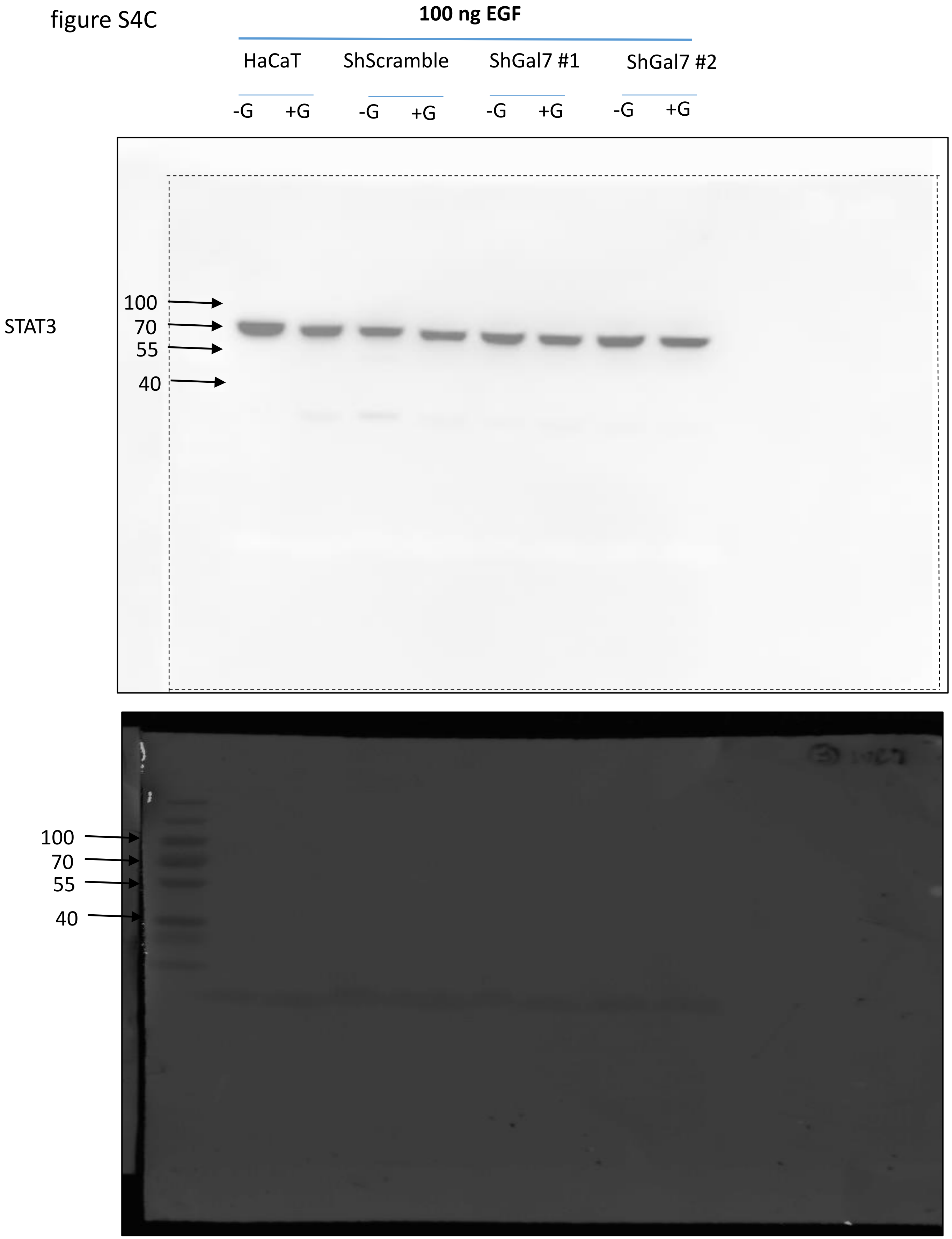

visible light membrane with molecular weight

figure S4C

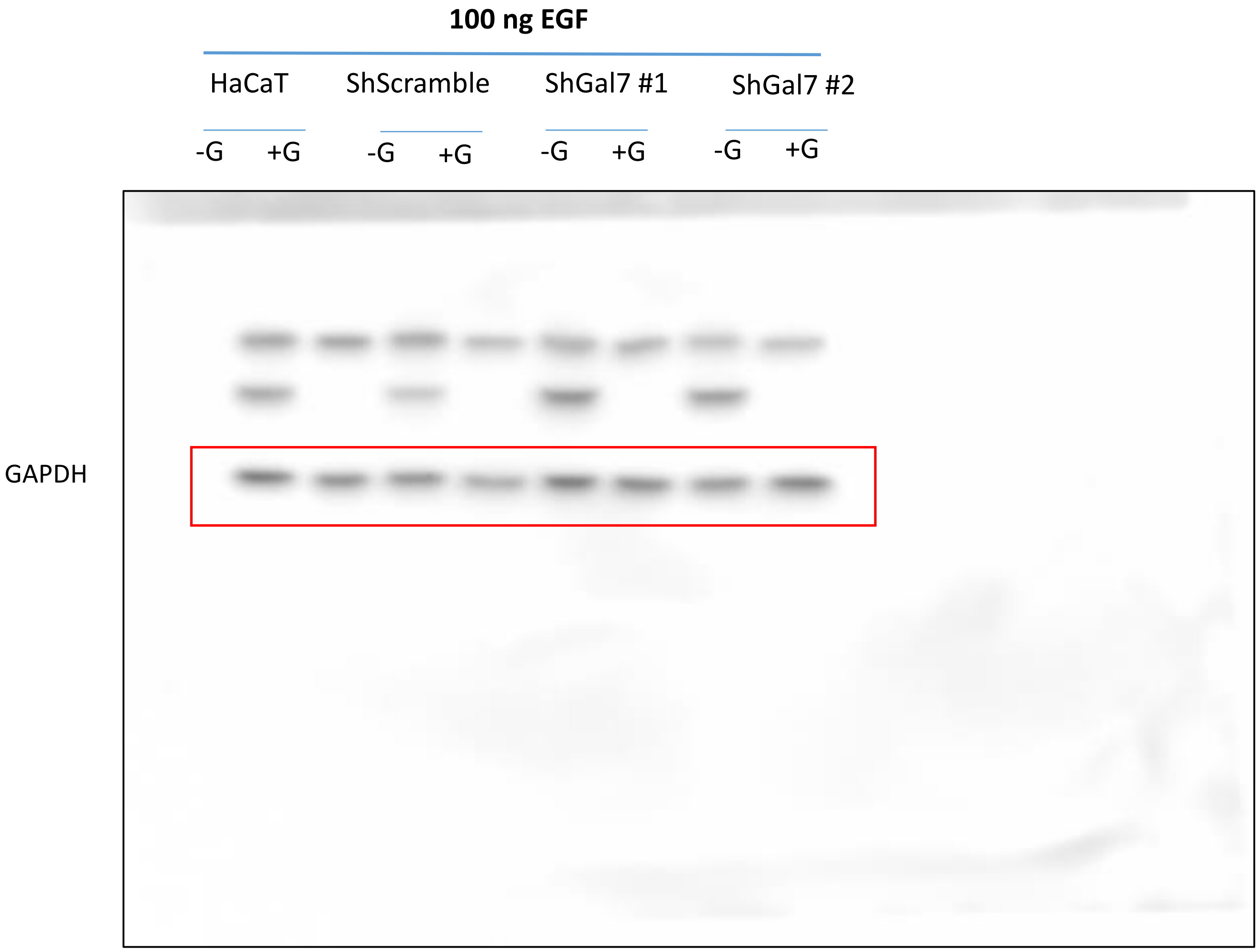

100 ng EGF
HaCaT
ShScramble
ShGal7 #1
ShGal7 #2
-G
+G
-G
+G
-G
+G
-G
+G
GAPDH

figure S4C

HaCaT ShScramble ShGal7 #1 ShGal7 #2

EGF ng/ml 0 100 0 100 0 100 0 100

P-stat3

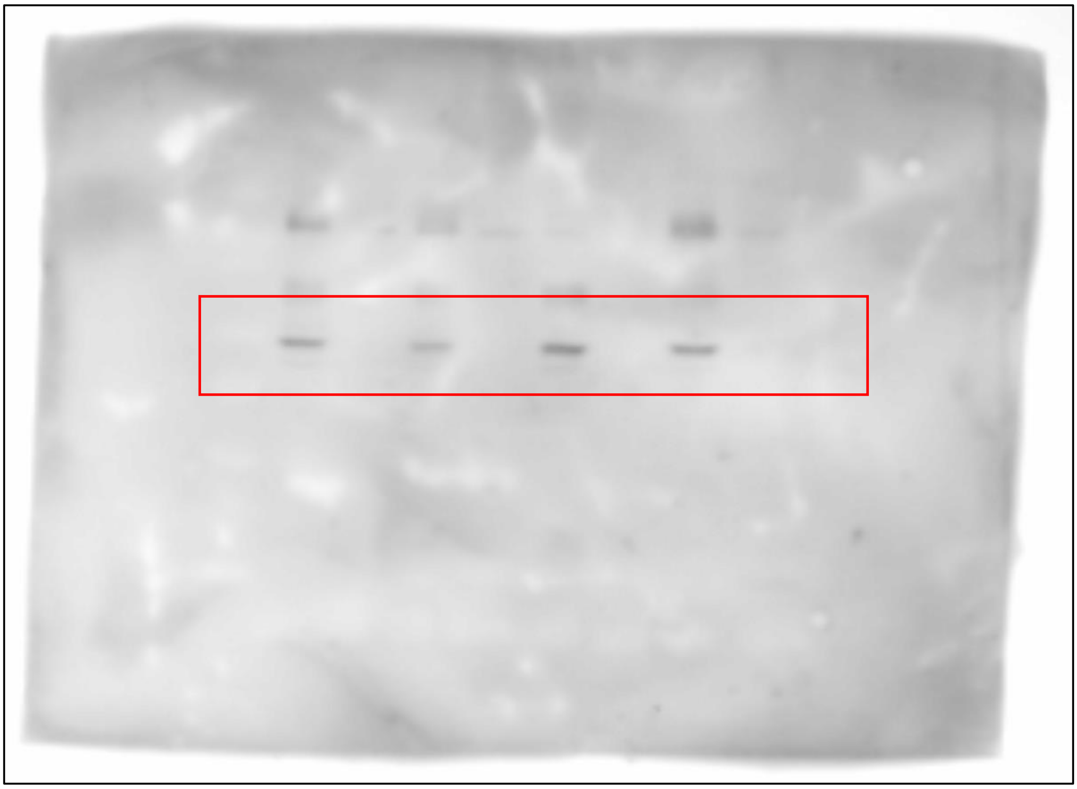

P-stat3
Lower exposure

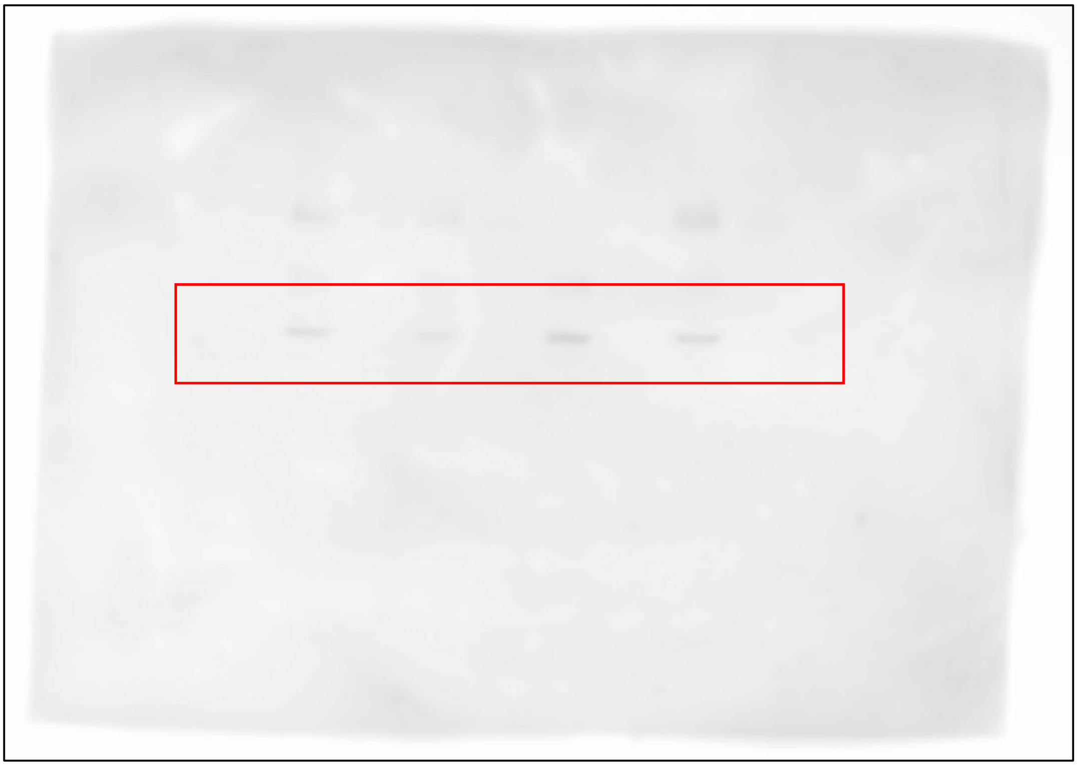

figure S4C

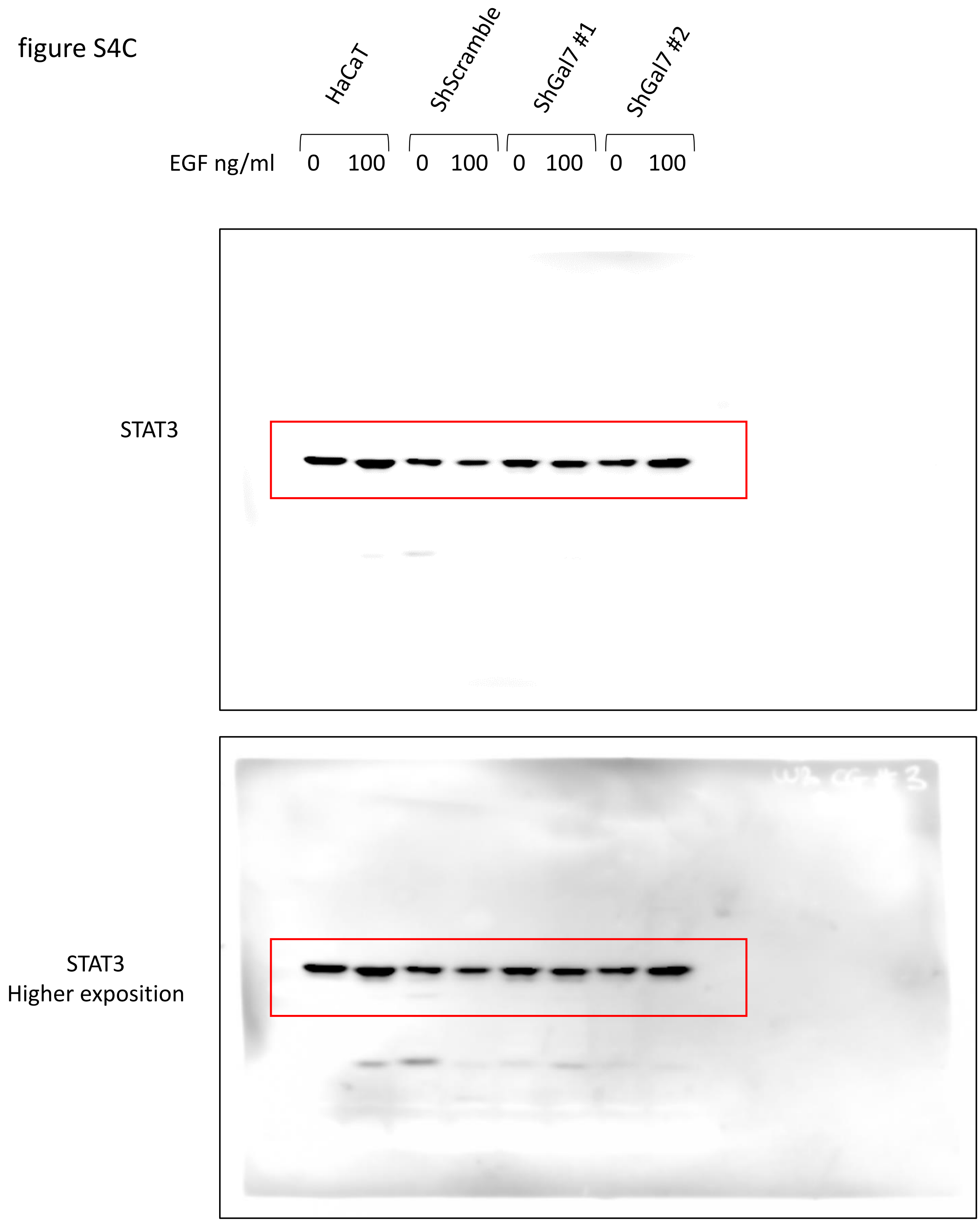
HaCaT
ShScramble
ShGal7 #1
ShGal7 #2
EGF ng/ml
0
100
0
100
0
100
0
100
STAT3
STAT3
Higher exposition

Figure S4D

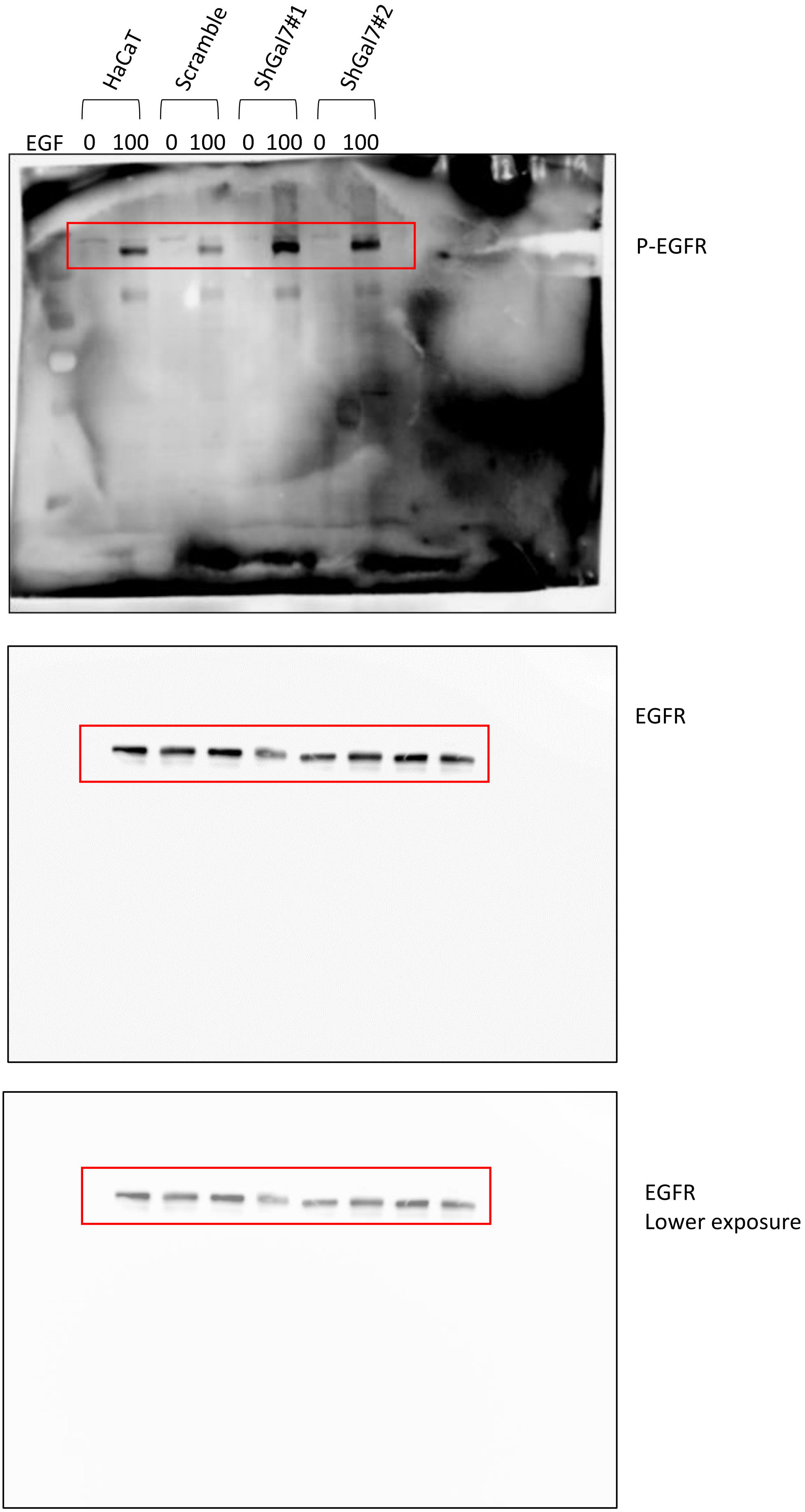
HaCaT
Scramble
ShGal7#1
ShGal7#2
EGF 0 100 0 100 0 100 0 100
P-EGFR
EGFR
EGFR
Lower exposure

Figure S4D

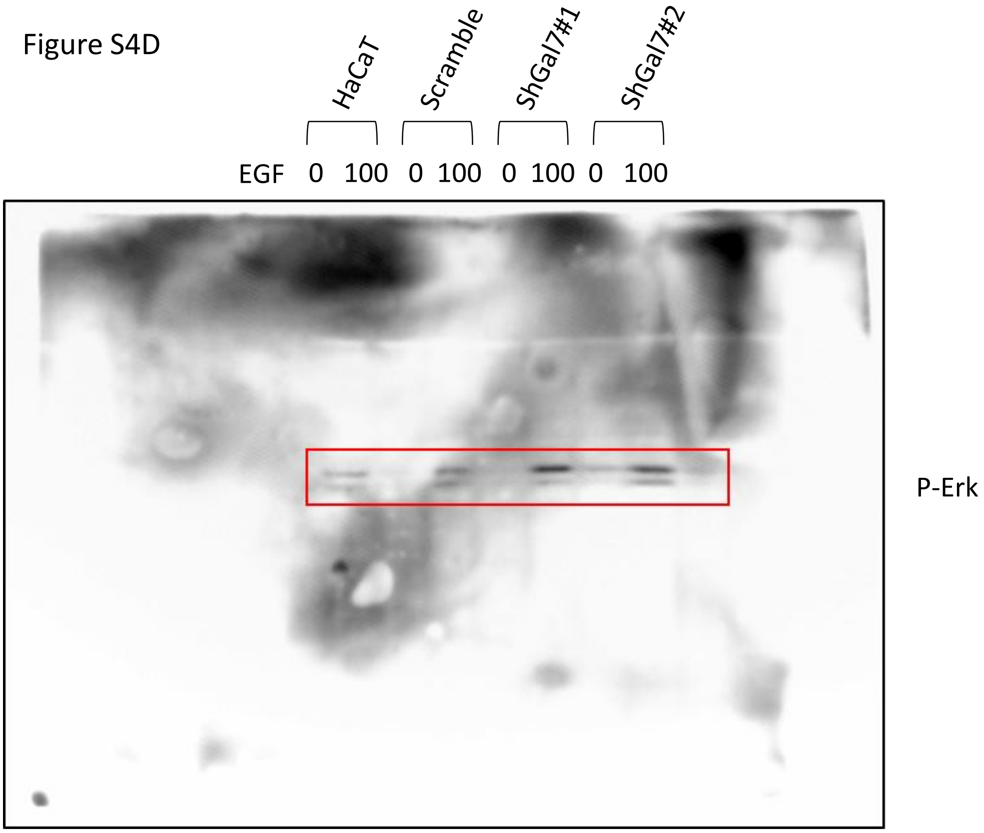
HaCaT
Scramble
ShGal7#1
ShGal7#2
EGF 0 100 0 100 0 100 0 100
P-Erk

Figure S4D

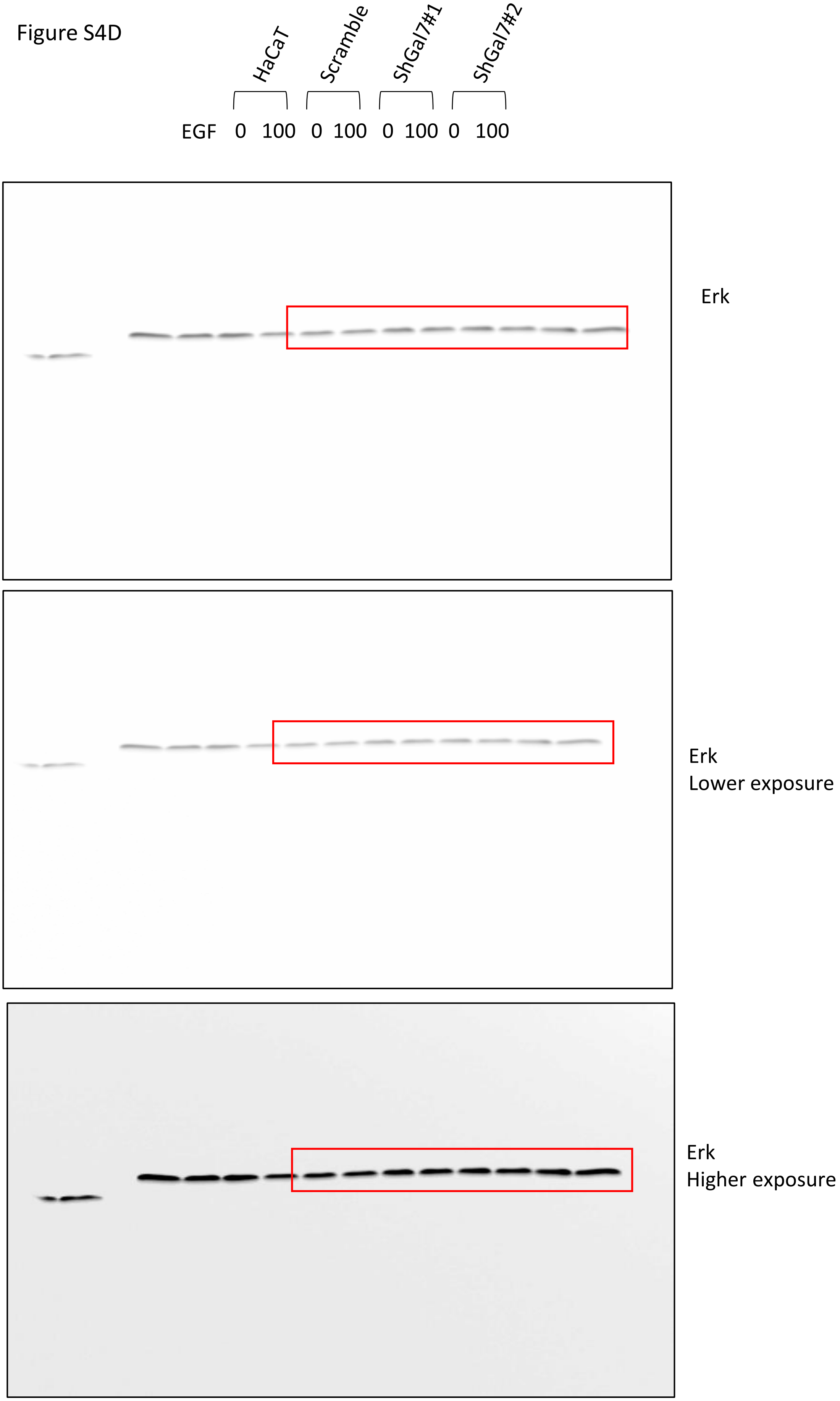
HaCaT
Scramble
ShGal7#1
ShGal7#2
EGF 0 100 0 100 0 100 0 100
Erk
Erk
Lower exposure
Erk
Higher exposure

Figure S4D

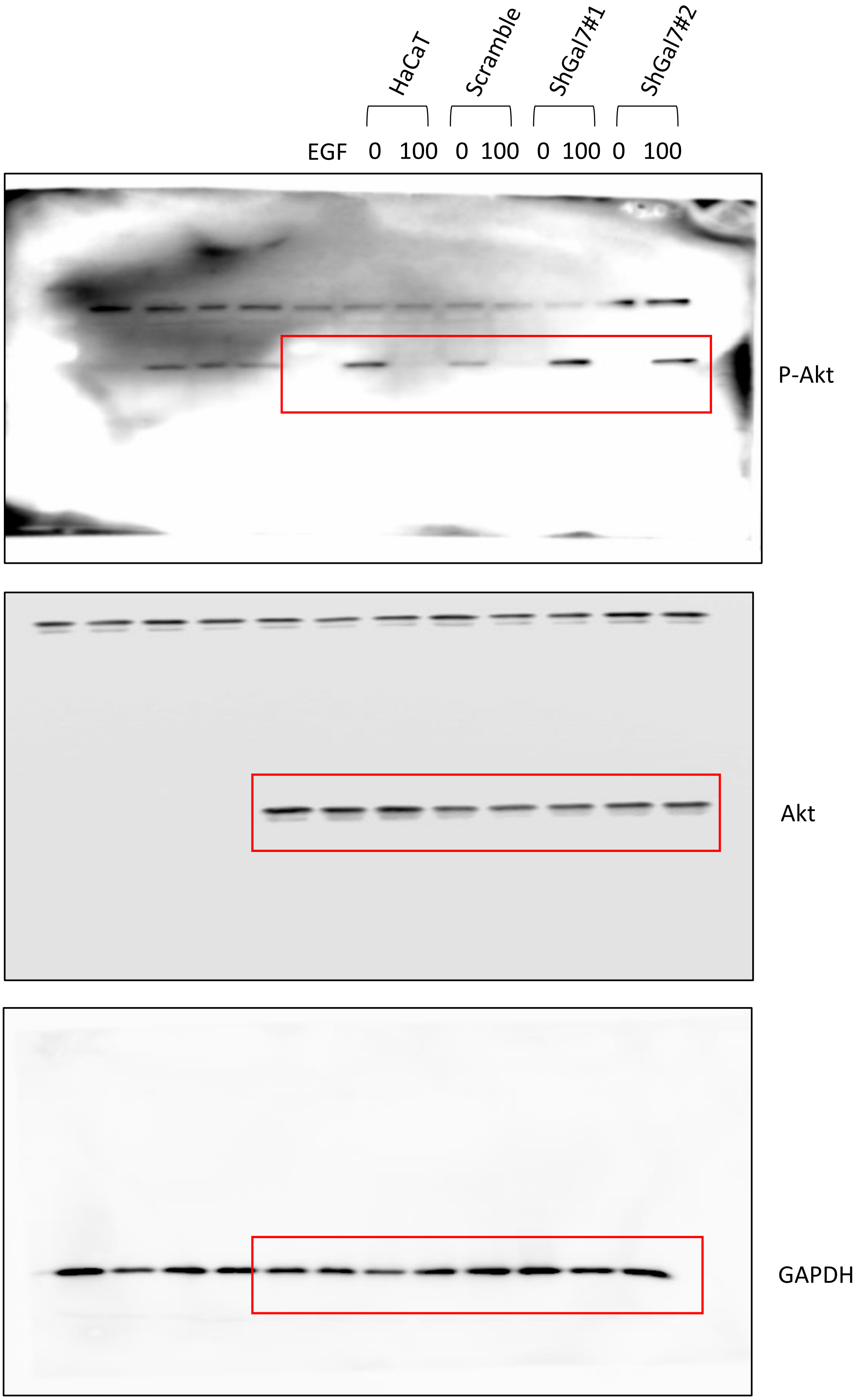
HaCaT
Scramble
ShGal7#1
ShGal7#2
EGF 0 100 0 100 0 100 0 100
P-Akt
Akt
GAPDH

Figure S4D

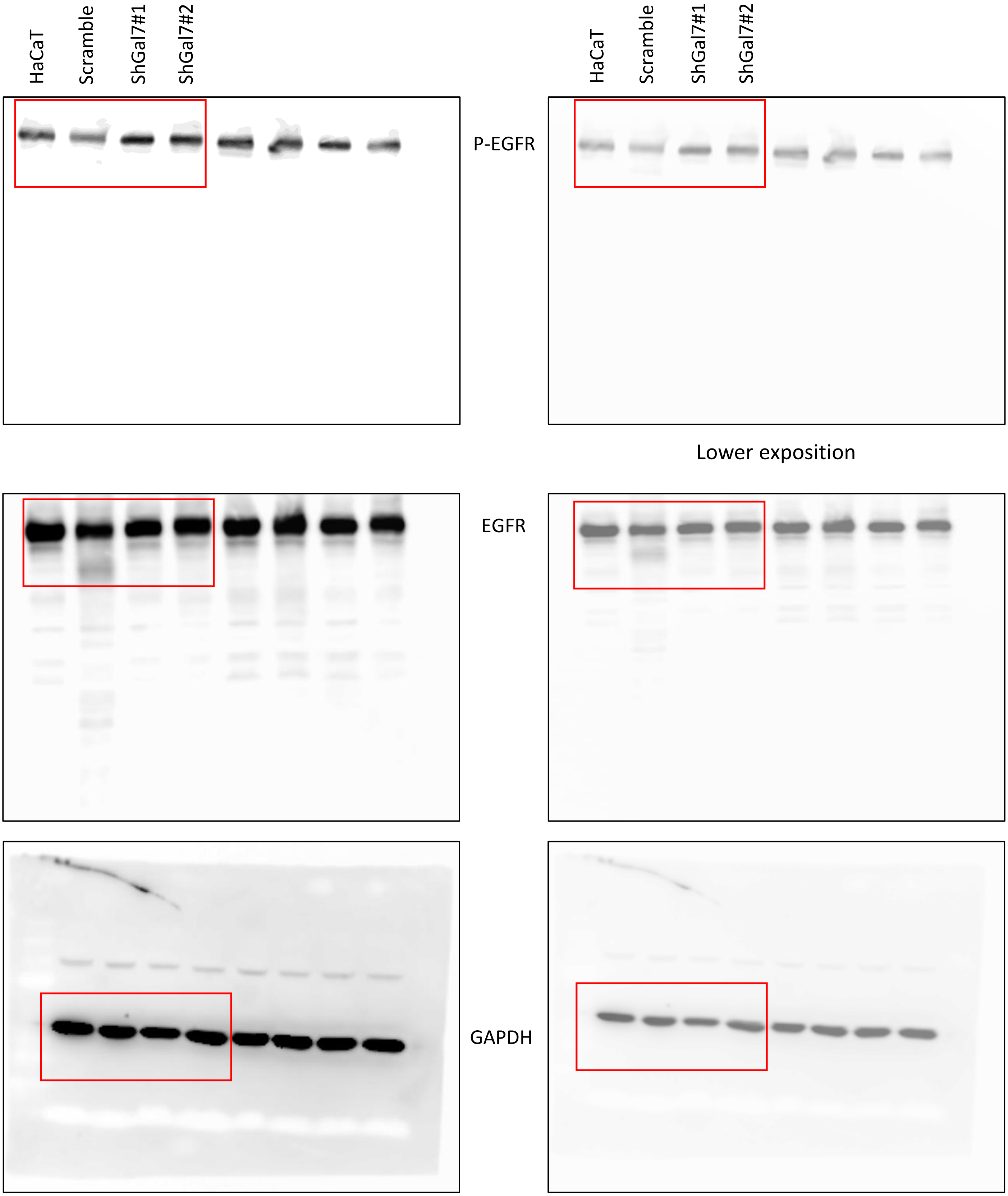
HaCaT
Scramble
ShGal7#1
ShGal7#2
P-EGFR
HaCaT
Scramble
ShGal7#1
ShGal7#2
Lower exposition
EGFR
GAPDH

Figure S4D

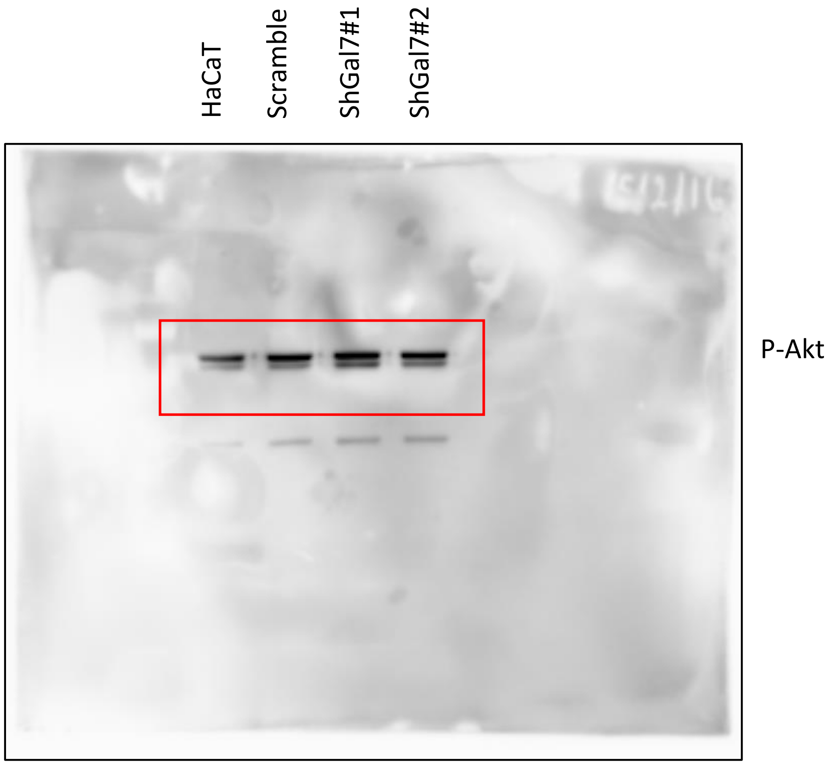
HaCaT
Scramble
ShGal7#1
ShGal7#2
P-Akt

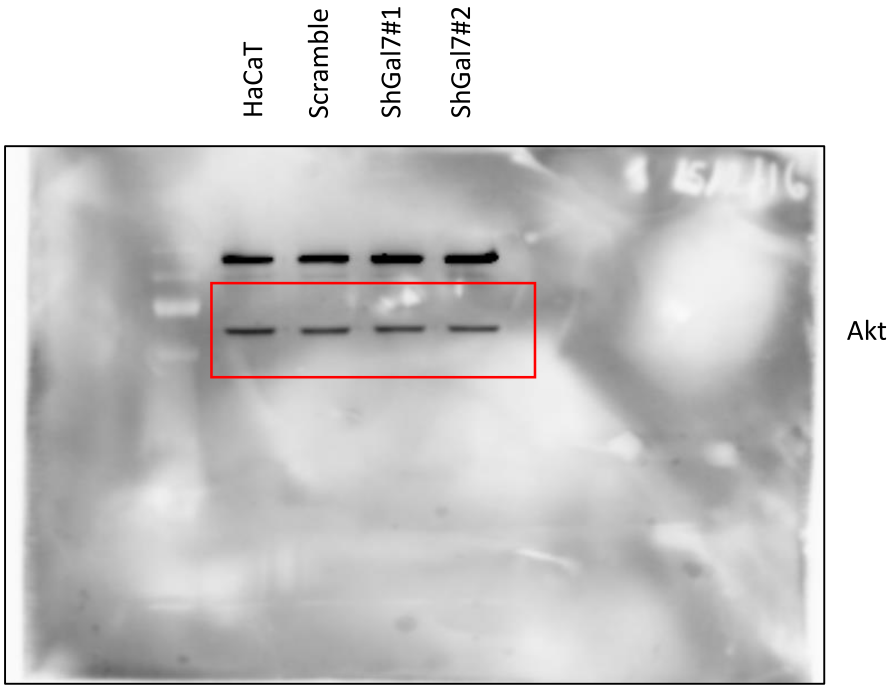
HaCaT
Scramble
ShGal7#1
ShGal7#2
Akt

Figure S4D

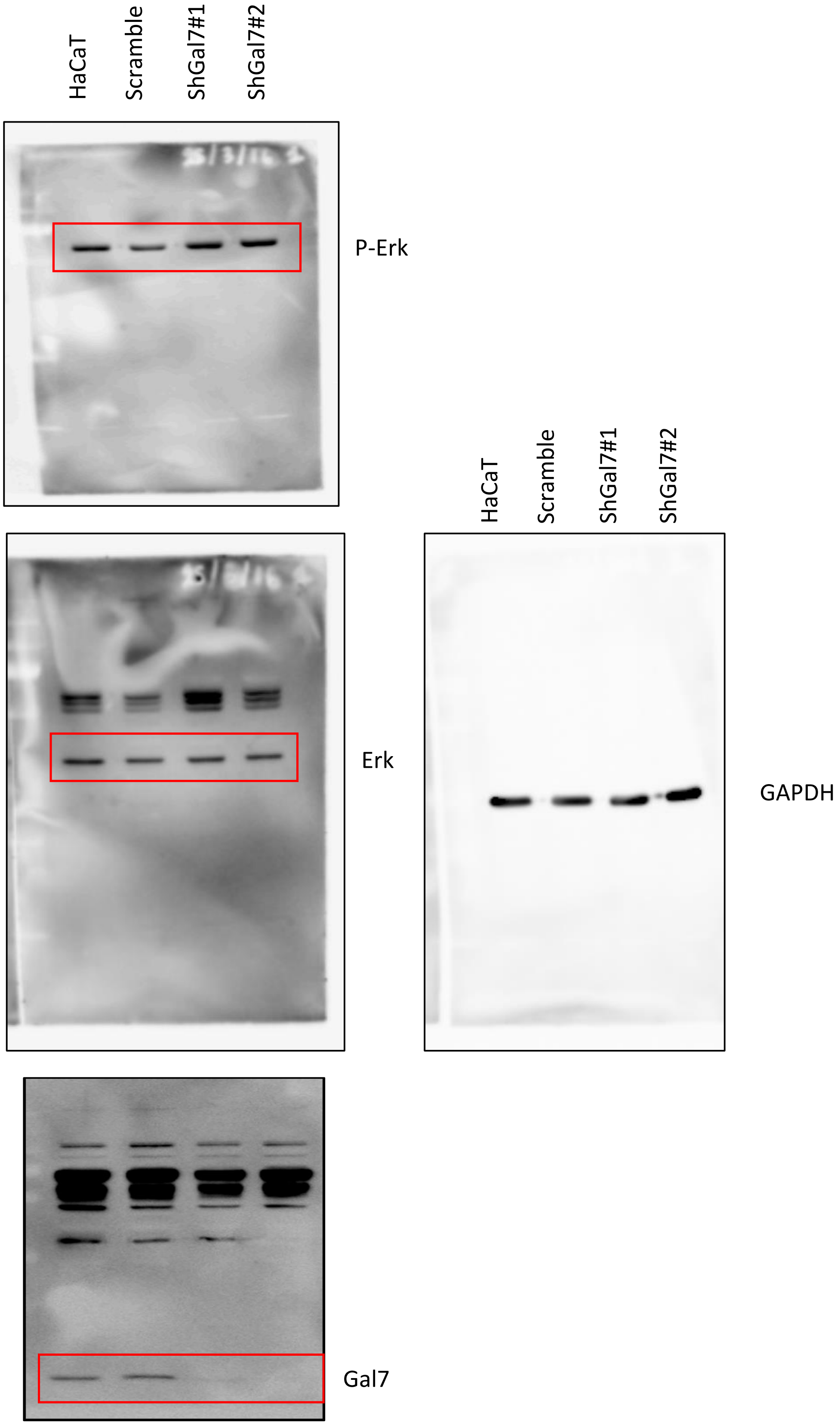

Figure S4E

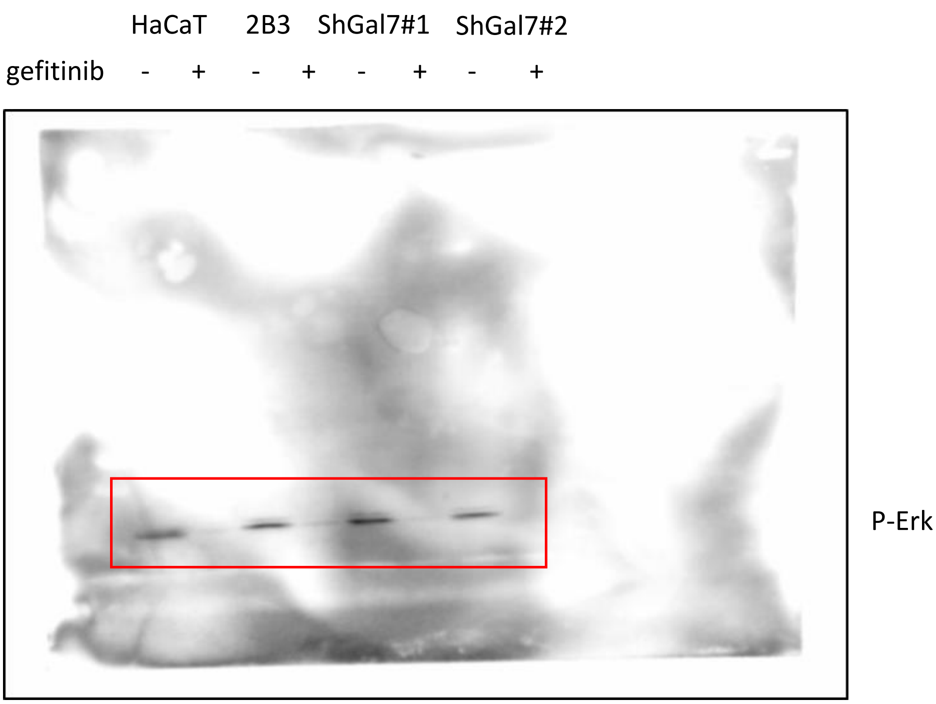

Figure S4E

HaCaT 2B3 ShGal7#1 ShGal7#2

gefitinib - + - + - + - +

55kDa →

40kDa →

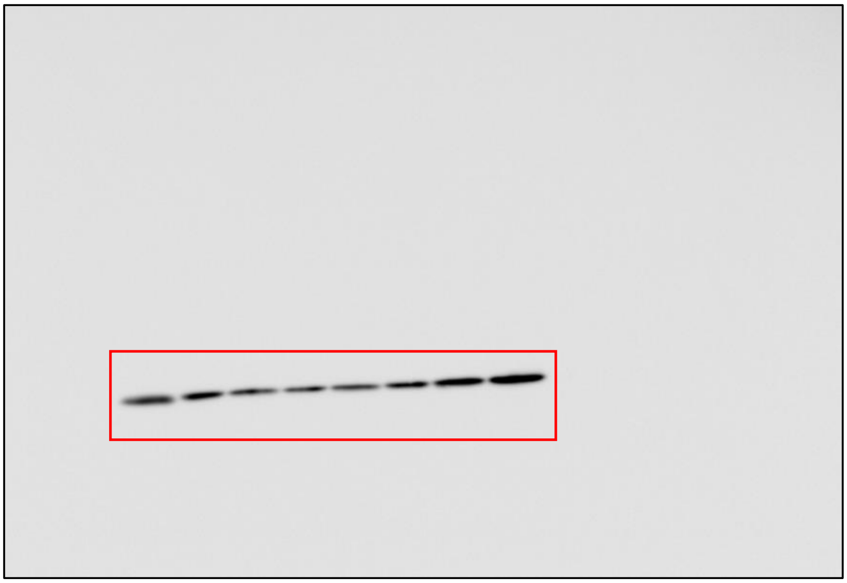

Erk

55kDa →

40kDa →

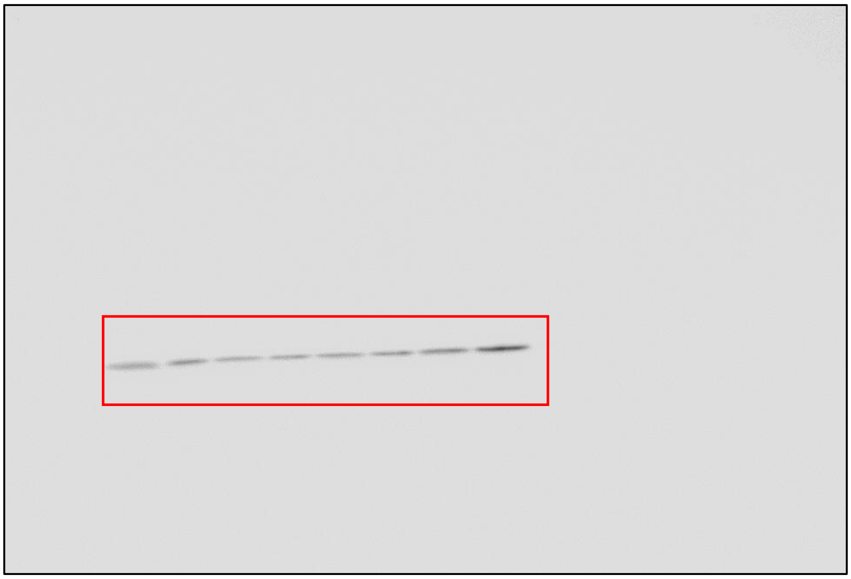

Erk
Lower exposition

55kDa →

40kDa →

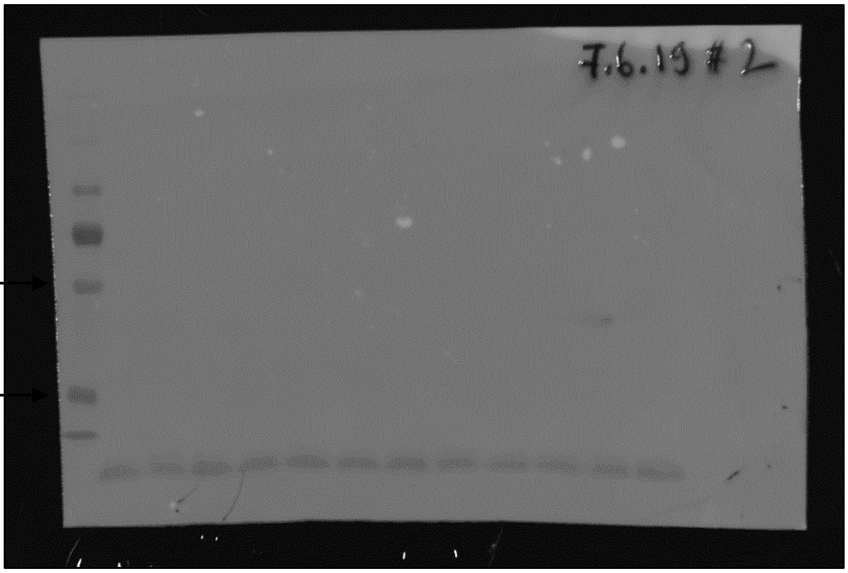

visible light
membrane with
molecular weight

Figure S4E

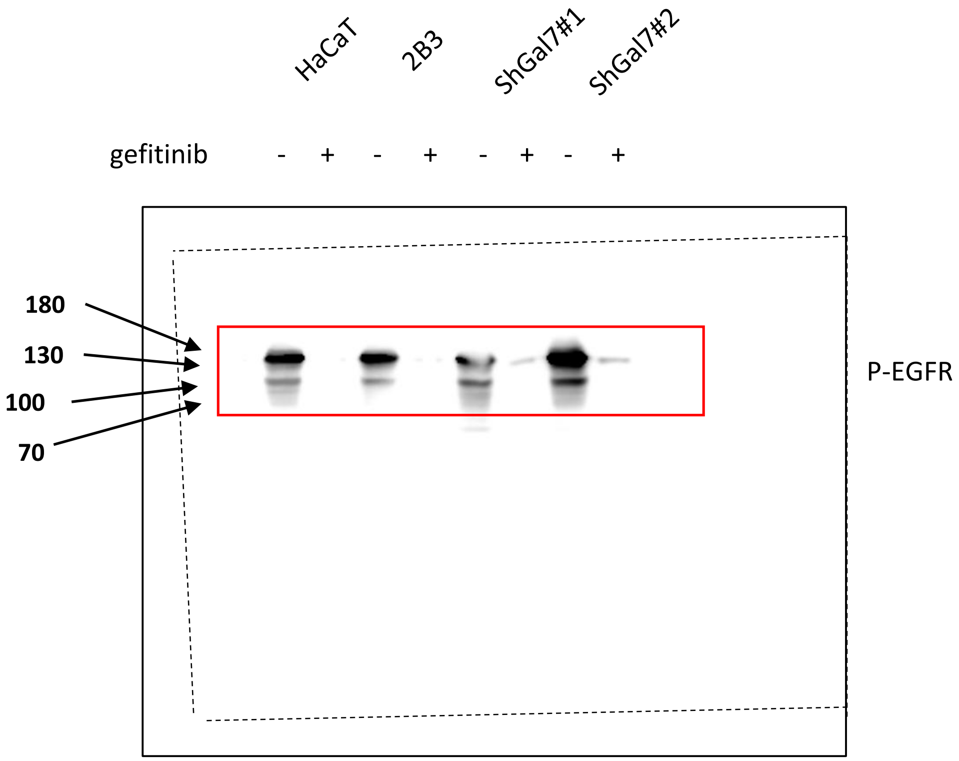

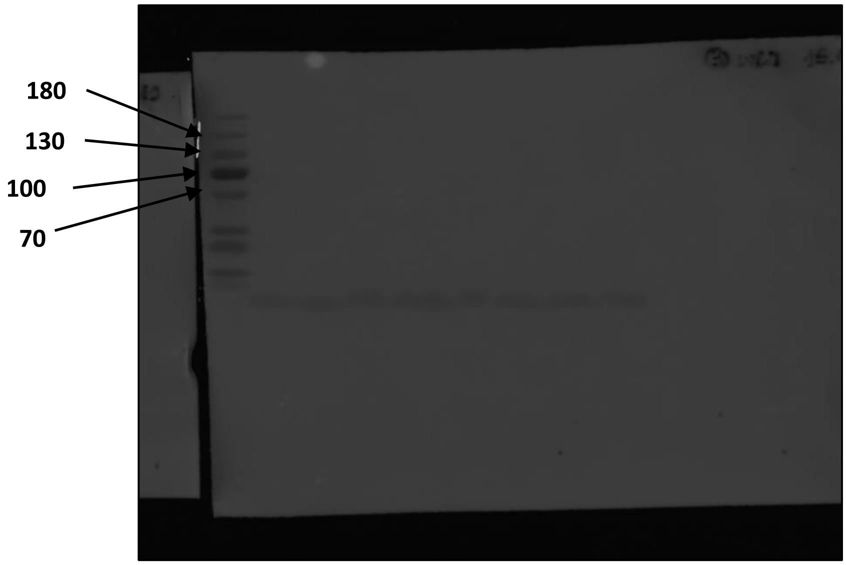

visible light membrane with molecular weight

Figure S4E

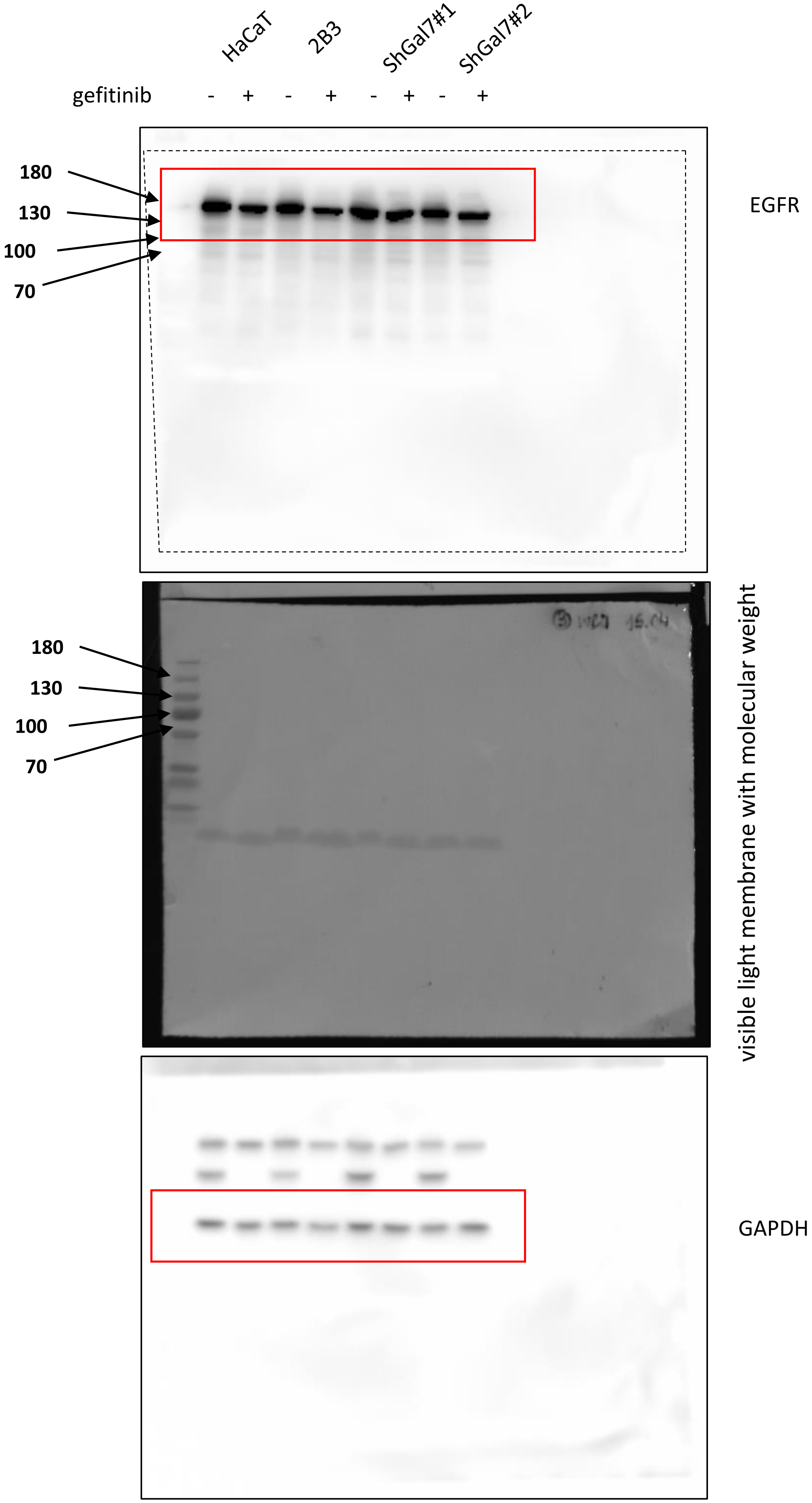
HaCaT
2B3
ShGal7#1
ShGal7#2
gefitinib
- + - + - + - +
180
130
100
70
EGFR
180
130
100
70
visible light membrane with molecular weight
GAPDH

Figure S4E

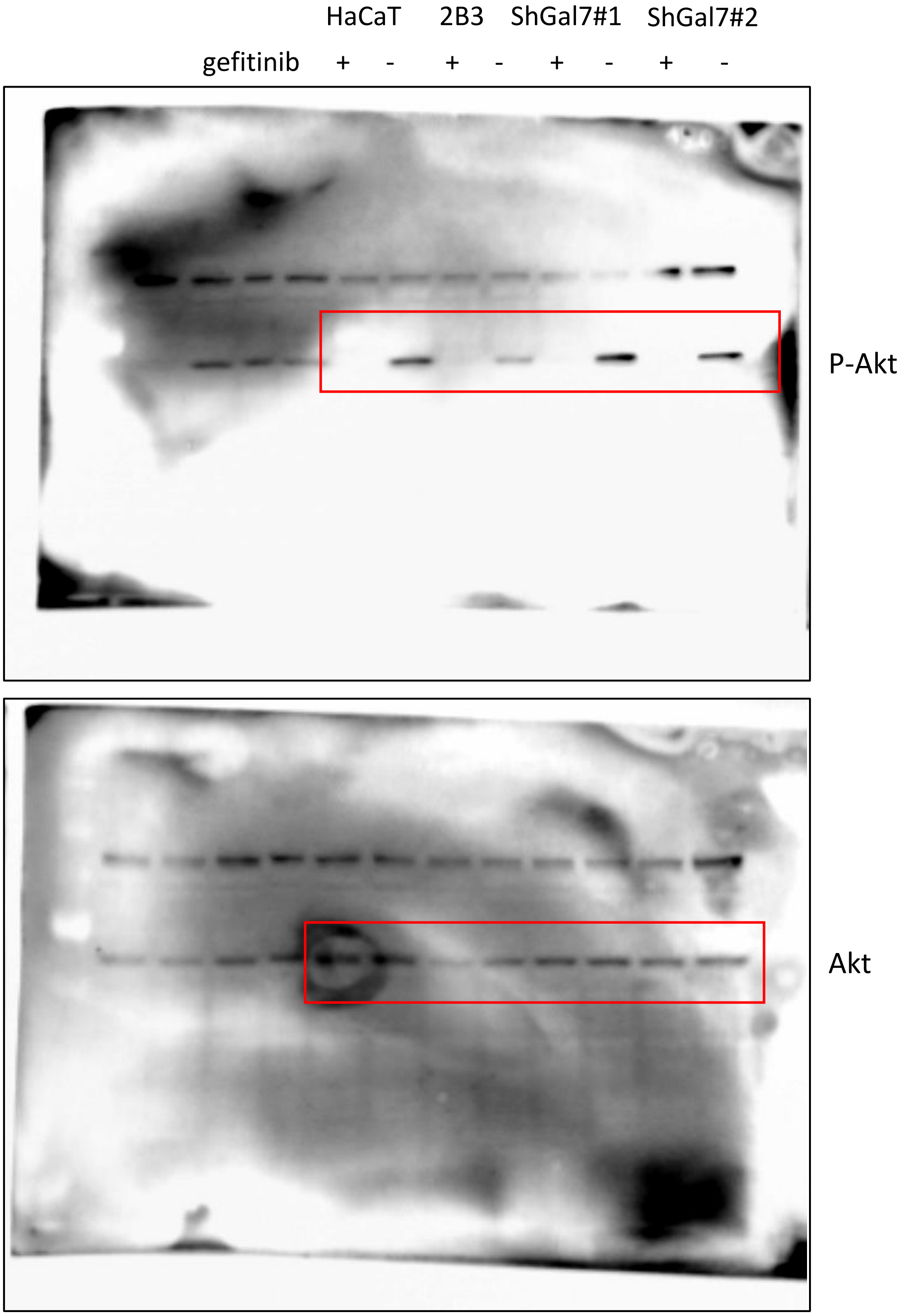

Figure S4: Alteration of EGFR downstream pathways by galectin-7 deficient cells. A/ Immunoblots were probed for Phospho Src (Y416), Total Src in presence or in absence of EGF. Gefitinib has been added at 15µM. Quantification of the blots are representative of at least 3 independant experiments. * p<0,1 **p<0,05 ***p<0,001 B/HaCaT cells were cultured in absence or in presence of 100ng/mL EGF and immunoblots were realized and probed for P-EGFR or EGFR. Quantification of the blots are representative of at least 3 independant expériments. * p<0,1 **p<0,05 ***p<0,001 C/ Immunoblots were probed for Phospho STAT3 and total STAT3 in presence or in absence of EGF. Gefitinib has been added at 15µM. Quantification of the blots are representative of at least 3 independant expériments. * p<0,1 **p<0,05 ***p<0,001 D/ Full-length blots presented in Fig. 4B. E/ Full-length blots presented in Fig. 4C.

Figure S5A

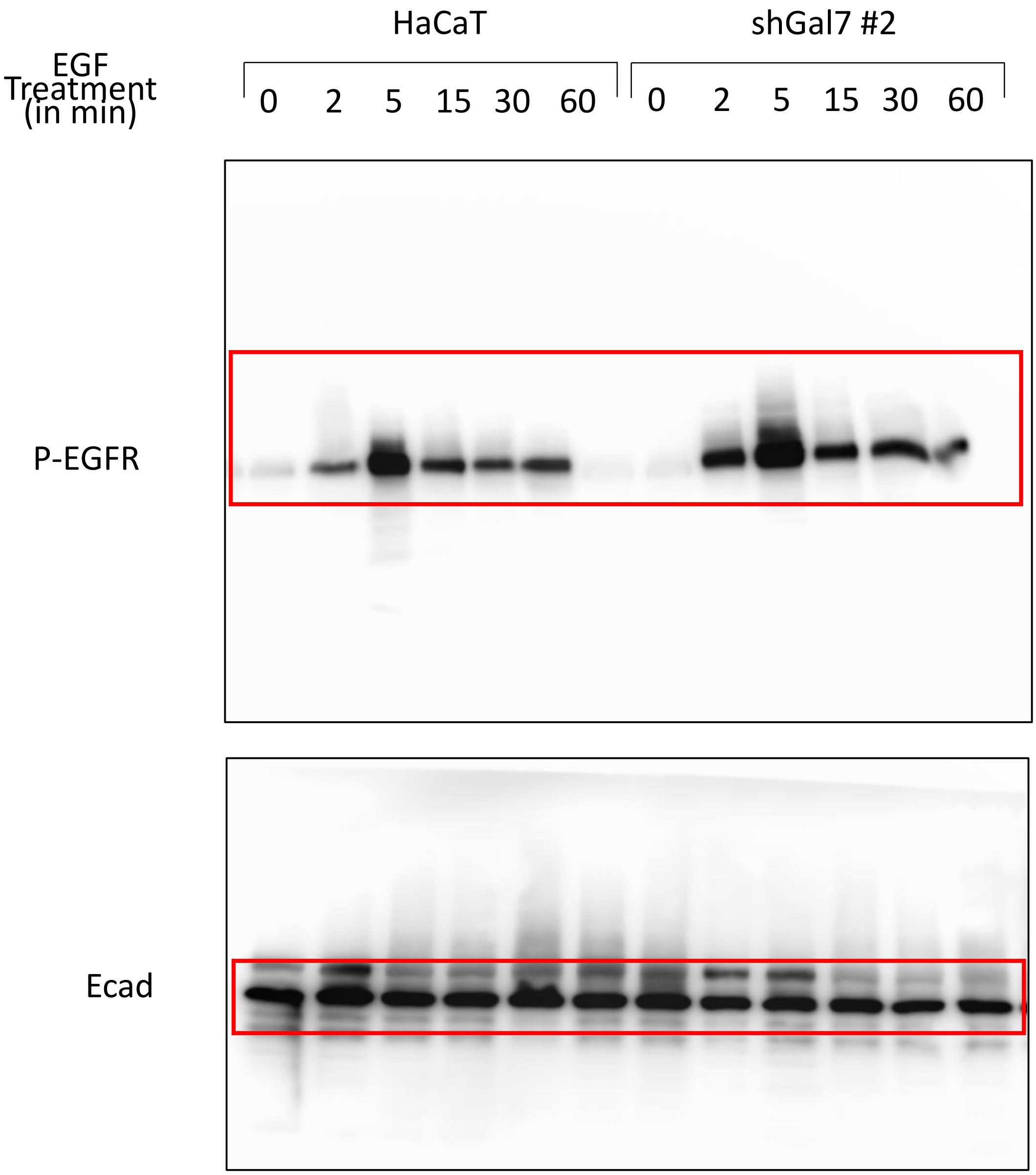
HaCaT
shGal7 #2
EGF Treatment (in min)
0 2 5 15 30 60
0 2 5 15 30 60
P-EGFR
Ecad

Figure S5A

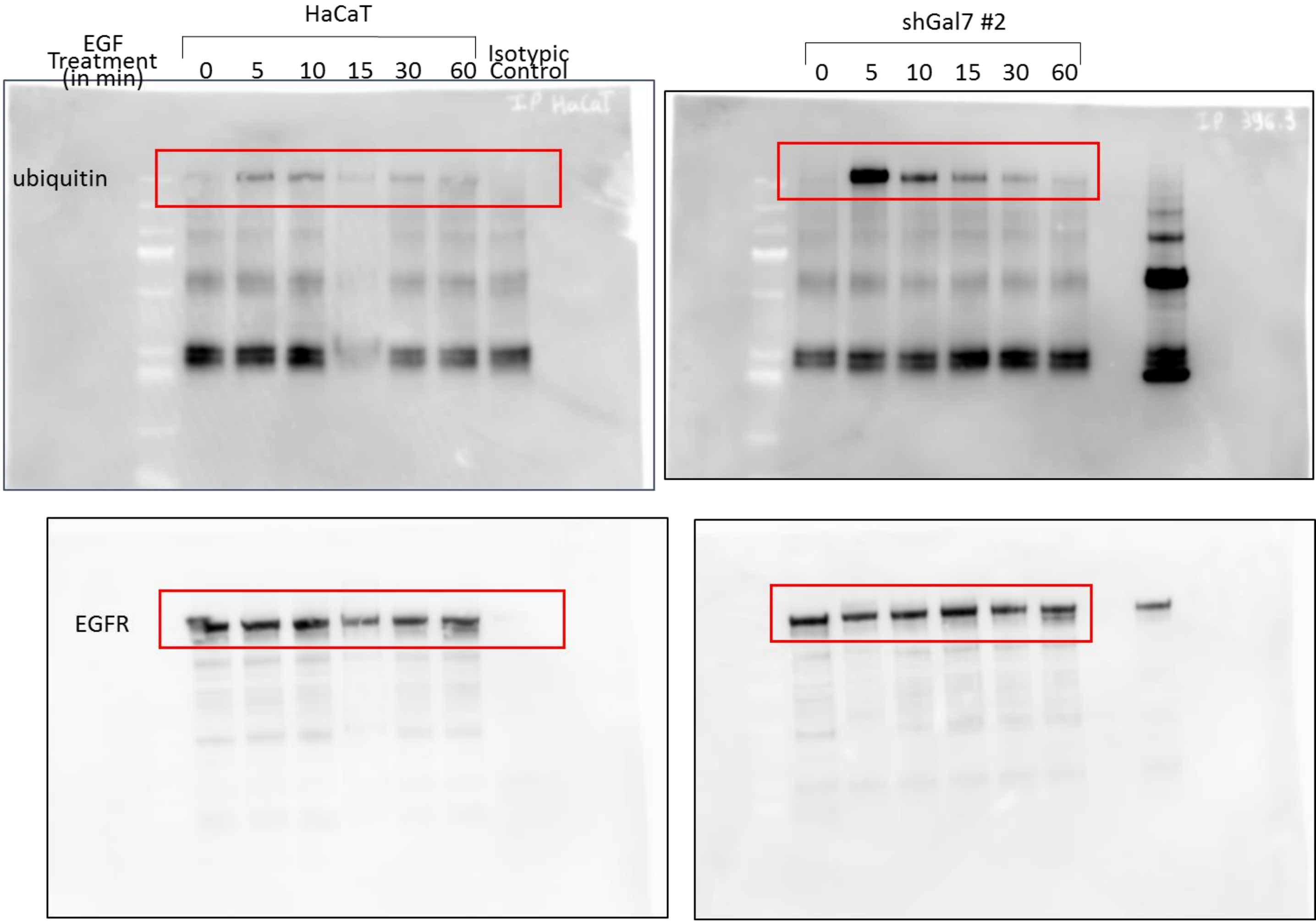
HaCaT
EGF Treatment (in min)
0
5
10
15
30
60
Isotypic Control
shGal7 #2
0
5
10
15
30
60
ubiquitin
EGFR
IP HaCaT
IP 396.3

Figure S5B

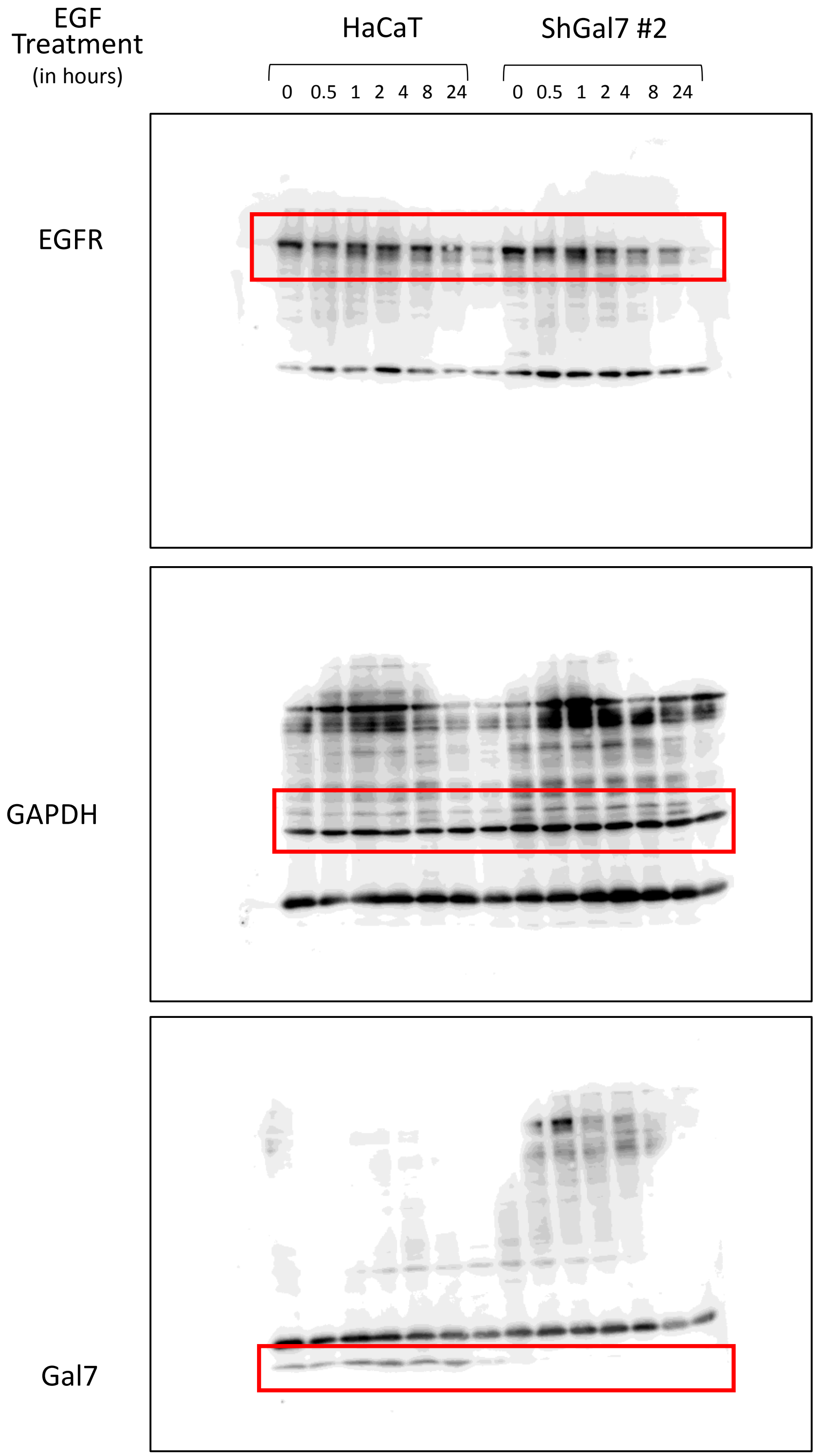

Figure S5 : A/ Full-length blots presented in Fig. 5A. B/ Full-length blots presented in Fig. 5B.

Figure S6A

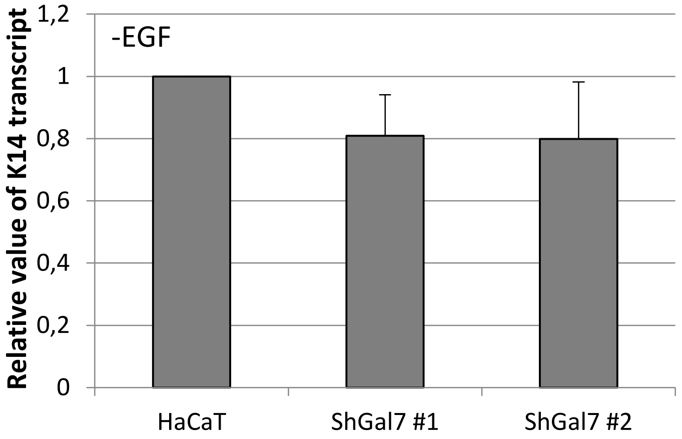

-EGF
Relative value of K14 transcript
0
0,2
0,4
0,6
0,8
1
1,2
HaCaT
ShGal7 #1
ShGal7 #2

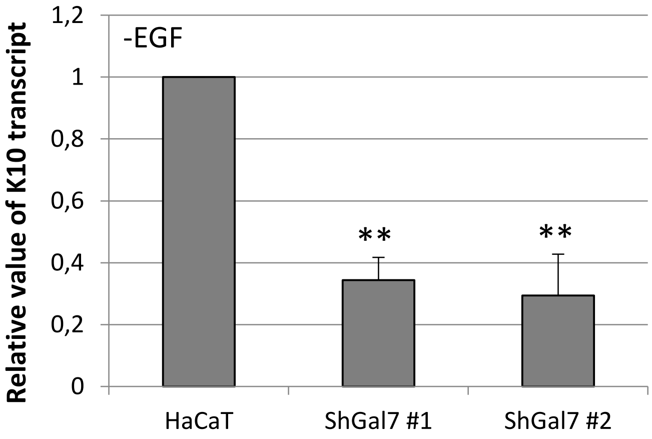

-EGF
Relative value of K10 transcript
0
0,2
0,4
0,6
0,8
1
1,2
**
**
HaCaT
ShGal7 #1
ShGal7 #2

Figure S6B

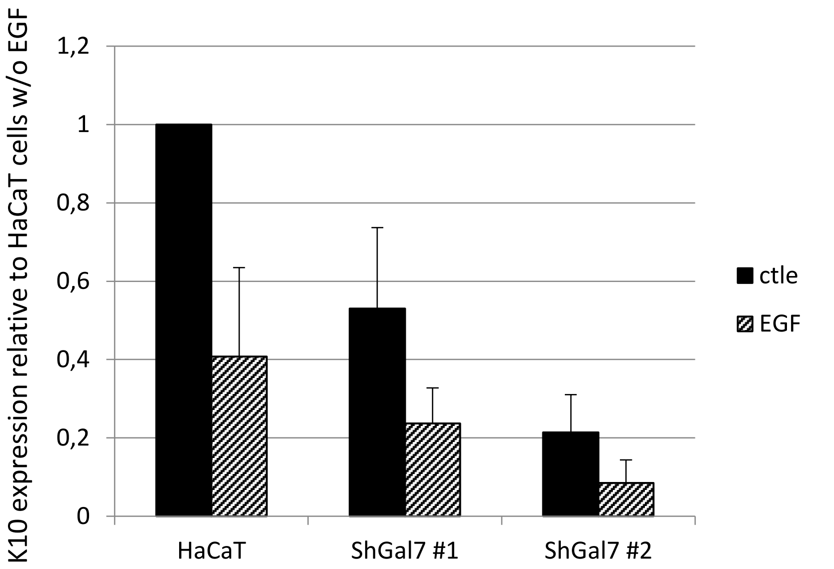

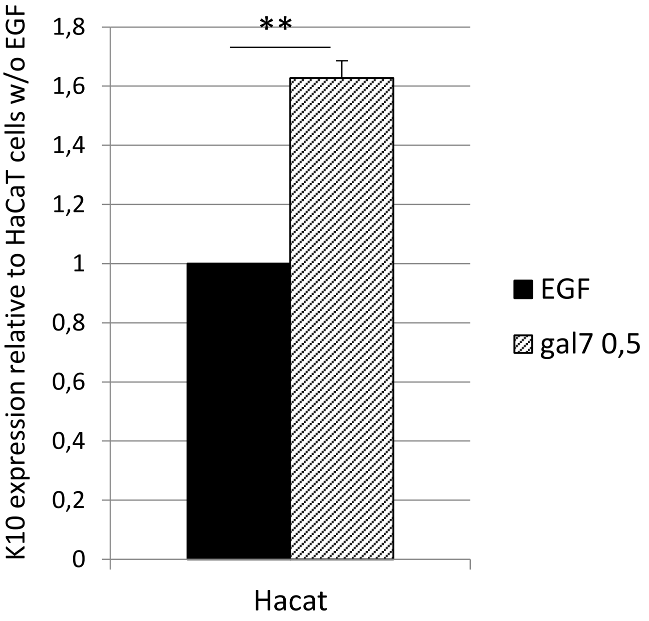

Figure S6 : Absence of galectin-7 induces a downregulation of K10. A/ Levels of K14 and K10 transcripts have been compared in HaCaT cell and in ShGal7 clones after cell culture reached confluence. The transcript levels have been quantified from three independent quantitative PCRs. The p-value has been calculated by an ANOVA statistical test. B/ Level of K10 transcript has been compared in HaCaT cell and in ShGal7 clones in presence or in absence of EGF treatment at 100ng/mL for 16 hrs in HaCaT cells 0.5mM rGal7 has been added for 16hrs for one condition. Transcript levels have been quantified from three independent quantitative PCRs. The p-value has been calculated by an ANOVA statistical test.
