# Supplementary material for: Identification of a new regulation pathway of EGFR and E-cadherin dynamics
Source: Sci Rep. 2021 Nov 22;11:22705. doi: 10.1038/s41598-021-02042-3 (PMC8609017; doi:10.1038/s41598-021-02042-3)
Supplement: Supplementary file 2 — Supplementary Information 2. [file 41598_2021_2042_MOESM2_ESM.docx]

Supplementary materials:

| **Antibody** | **provider** | **reference** |  |
| --- | --- | --- | --- |
| Galectin-7 | Abcam | ab10482 |  |
| EGFR | Abcam | ab52894 |  |
| P-EGFR | Abcam | ab40815 |  |
| P-Akt | Santa-Cruz | sc-7985 |  |
| Akt | Cell signalling | 4691 |  |
| Erk | Santa-Cruz | sc-154 |  |
| P-ERk | Santa-Cruz | sc-7383 |  |
| P-Src | Cell signalling | 6943 |  |
| Src | Cell signalling | 2109 |  |
| STAT3 | Santa Cruz | sc-293151 |  |
| P-STAT3 | Santa Cruz | sc-8059 |  |
| Caveolin-1 | Cell signalling | 3267 |  |
| CD63 | Ancell | 215-020 |  |
| Lamp-1 | Santa-Cruz | sc-20011 |  |
| E-cadherin | BD Biosciences | 610181 |  |
| GAPDH | Thermo Scientific | MA5-15738 |  |
| Anti-rabbit HRP | GE Healthcare | NA934V |  |
| Anti-mouse HRP | Sigma | A9044 |  |
| Anti-rabbit Alexa 488 | Invitrogen | A11034 |  |
| Anti-mouse Alexa 488 | Invitrogen | A11001 |  |
| Anti-rabbit Alexa 568 | Invitrogen | A11011 |  |
| Anti-mouse Alexa 568 | Invitrogen | A11004 |  |
| Keratin-10 | Covance | MMS-159S |  |
| Keratin-14 | Covance | PRB-155P |  |
| Transferrin fluorescein | Rockland | 00090234 |  |
